# Developing a Sustainable Cardiovascular Disease Research Strategy in Tanzania Through Training: Leveraging From the East African Centre of Excellence in Cardiovascular Sciences Project

**DOI:** 10.3389/fcvm.2022.849007

**Published:** 2022-03-25

**Authors:** Pilly Chillo, Fredirick Mashili, Gideon Kwesigabo, Paschal Ruggajo, Appolinary Kamuhabwa

**Affiliations:** ^1^Department of Internal Medicine, Muhimbili University of Health and Allied Sciences, Dar es Salaam, Tanzania; ^2^East African Centre of Excellence in Cardiovascular Sciences, Muhimbili University of Health and Allied Sciences, Dar es Salaam, Tanzania; ^3^Department of Physiology, Muhimbili University of Health and Allied Sciences, Dar es Salaam, Tanzania; ^4^Department of Epidemiology and Biostatistics, Muhimbili University of Health and Allied Sciences, Dar es Salaam, Tanzania; ^5^Department of Clinical Pharmacy and Pharmacology, Muhimbili University of Health and Allied Sciences, Dar es Salaam, Tanzania

**Keywords:** cardiovascular disease, cardiovascular research, Tanzania, research training, research strategy, sub-Saharan Africa, center of excellence

## Abstract

**Introduction:**

Cardiovascular disease (CVD) contribute the largest mortality burden globally, with most of the deaths (80% of all deaths) occurring in low and middle-income countries (LMICs), including Tanzania. Despite the increasing burden, to date, CVD research output is still limited in Tanzania, as it is for many sub-Saharan Africa (SSA) countries. This trend hinders the establishment of locally informed CVD management and policy changes. Here, we aim to review the existing gaps while highlighting the available opportunities for a sustainable CVD research strategy in Tanzania.

**Methods:**

A rapid review of available literature on CVD research in SSA was conducted, with emphasis on the contribution of Tanzania in the world literature of CVD. Through available literature, we identify strategic CVD research priorities in Tanzania and highlight challenges and opportunities for sustainable CVD research output.

**Findings:**

Shortage of skilled researchers, inadequate research infrastructure, limited funding, and lack of organized research strategies at different levels (regional, country, and institutional) are among the existing key bottlenecks contributing to the low output of CVD research in Tanzania. There is generally strong global, regional and local political will to address the CVD epidemic. The establishment of the East African Centre of Excellence in Cardiovascular Sciences (EACoECVS) offers a unique opportunity for setting strategies and coordinating CVD research and training for Tanzania and the East African region.

**Conclusion:**

There is a light of hope for long-term sustainable CVD research output from Tanzania, taking advantage of the ongoing activities and plans for the evolving EACoECVS. The Tanzanian experience can be taken as a lesson for other SSA countries.

## Introduction

Cardiovascular disease (CVD) account for most non-communicable diseases (NCDs) deaths globally, an estimated 17 million people each year with 80% of these deaths occurring in low and middle-income countries (LMICs) (1). In sub-Saharan Africa (SSA) countries including Tanzania, cardiac diseases, chronic kidney disease, and stroke have been increasing rapidly (2) and now are important causes of morbidity, mortality and disability-adjusted life years (DALYs) lost among adults (1, 3). These three major CVD are driven by common risk factors –mainly hypertension, which occurs in one out of every four adults in Tanzania (4). Other important risk factors include diabetes mellitus, obesity, sedentary lifestyle, and cigarette smoking, all a result of an epidemiological transition that is currently taking place in the country (4, 5). Furthermore, in SSA there are still some CVD related to malnutrition, poverty and infections such as rheumatic heart disease, cardiomyopathies, untreated congenital heart diseases, as well as human immunodeficiency virus (HIV) and tuberculosis-related CVD (6). This results in a double burden of “old” and “new” lifestyle-related CVD. Africa has additional risks for CVD caused by inherently genetic diseases that are commonly found in this part of the world, like sickle cell anemia which causes systemic complications, including heart failure, kidney failure, pulmonary complications, and stroke (7).

When compared to Caucasians, CVD occur early among people of African origin, and with fast progression to end stage organ damage (6, 8). This causes major economic development challenges as well as direct or indirect hindrance to achieving sustainable millennium development goals one (no poverty), two (zero hunger), three (good health and well-being), four (quality education), eight (descent work and economic growth), and ten (reduced inequalities) (9). This is due to the economic drain brought about by loss of a productive work force, but also through high costs of treating the chronic conditions at individual, communities and national level.

**FIGURE 1:**
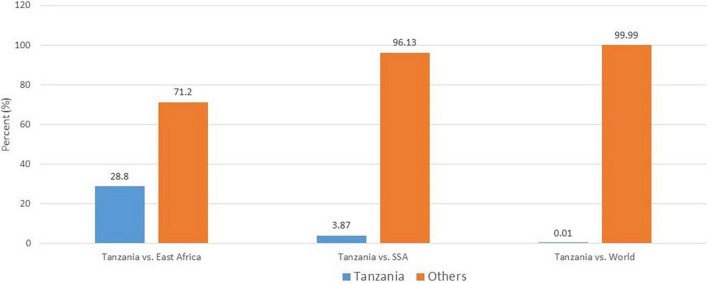
Cardiovascular disease research output from Tanzania compared to East Africa, sub-Saharan Africa, and World (PubMed search 2000–2021). Number of articles extracted: Tanzania, 196; East Africa, 681; sub-Saharan Africa, 142,395; World, 1,355,322.

Although not yet fully implemented, there is already some level of commitment to combat the increasing challenge of CVD both regionally and in Tanzania. NCDs including CVD have been recognized as one of the biggest threats to economic development that Africa is striving to achieve. At the continent level, African Ministers of Health have pledged their commitment to work together in fighting the diseases (10). In Tanzania, an NCDs special unit has been created at the Ministry of Health (11). This unit is dedicated to creating awareness and to oversee efforts to prevent NCDs and their associated risk factors. At the level of the East African Community, several initiatives including establishment of East African Centres of Excellences for NCDs including CVD, cancer and renal diseases are being implemented in different countries within the community (12). In this initiative, Tanzania has been given a task to develop an East African Centre of Excellence in Cardiovascular Sciences (EACoECVS). Fighting against CVD needs collective efforts. The Tanzanian Ministry of Health needs evidence-based research to inform the magnitude of the problem as well as findings from local communities in order to effectively implement preventive and control measures against CVD. However, until now there is limited research output from Tanzania addressing CVD, evidenced by the small contribution of scientific research articles emanating from Tanzania in the world literature (Figure 1). Additionally, even with the available research, there is lack of streamlined and focused research that addresses a common goal. Being the foremost health university in Tanzania and the host of the evolving EACoECVS, Muhimbili University of Health and Allied Sciences (MUHAS) is positioned to play a central role in conducting CVD research as well as train a critical mass of researchers to conduct CVD research. This will help to address two important gaps in CVD research in Tanzania, namely limited research output and shortage of researchers in CVD.

In this review, we put forward the centre’s CVD research agenda and we describe how to utilize the existing opportunities for sustainable research output, while addressing the challenges in terms of academia, research training, research infrastructure and funding faced by many SSA countries, taking Tanzania as an example.

## Methods

A rapid review of literature was conducted on the topic of CVD research in SSA. Search databases used were PubMed, African Journals Online and Google Scholar. Firstly, the PubMed database was used to assess the contribution of Tanzania in CVD research output in comparison to East Africa, SSA and globally. The PubMed search terms used were “cardiovascular diseases [MeSH Terms],” “Africa, sub Saharan [MeSH Terms],” “((((Kenya [MeSH Terms]) OR (Rwanda [MeSH Terms])) OR (Burundi [MeSH Terms])) OR (Uganda [MeSH Terms])) OR (Tanzania [MeSH Terms]),” and “united republic of Tanzania [MeSH Terms].” Filters were added to confine the PubMed search from 2000 to 2021 (Supplementary File 1). Search terms in African Journals Online and Google Scholar were: “cardiovascular diseases,” “cardiovascular research” “sub Saharan Africa,” “Tanzania,” “research training,” and “research strategy.” The literature obtained from PubMed and other databases searched were then critically examined to summarize information on CVD research priorities in Tanzania, as well as challenges and opportunities for CVD research in SSA, including Tanzania. All literature including original papers, perspectives, topic reviews, and conferences proceedings that focused on SSA CVD research were examined.

As part of the activities of the evolving EACoECVS, information on research priorities, action points for implementation, available opportunities as well as current challenges and their possible mitigations was obtained from several internal and external meetings/workshops with stakeholders of CVD in Tanzania and East Africa, including meetings of the East African Community Sectoral Council of Ministers of Health.

## Defining the Research Agenda for the East African Centre of Excellence in Cardiovascular Sciences

### Goal and Objectives

The overall goal of the centre’s research agenda is to contribute toward prevention and control of CVD in Tanzania and the East African region and eventually decrease morbidity and mortality resulting from these diseases. The immediate main objective is to train a critical mass of researchers to lead research in key strategic areas in order to identify and propose solutions to reduce the CVD burden in Tanzania and the East African region.

### Research Priorities

Cardiovascular disease cover a broad range of conditions affecting the heart and blood vessels. In Tanzania, hospital data show that highly prevalent CVD include hypertensive heart disease leading to heart failure, hypertensive kidney disease leading to end stage renal failure as well as cerebrovascular diseases presenting as stroke (13–15). Other important CVD include rheumatic heart disease, cardiomyopathies – particularly dilated cardiomyopathy, untreated congenital heart diseases, endomyocardial fibrosis, and cardiovascular complications of sickle cell anemia, tuberculosis and HIV disease (7, 16–18). Although not yet ranking highest in Tanzania, ischemic heart disease is on the increase (19). With time, ischemic heart disease is likely to consume a significant amount of the already inadequate budget on health (20).

Here we define specific CVD research areas that despite existing efforts are still facing significant unmet needs. Epidemiological and scientific knowledge coupled with translation into policy and clinical implementation will have major impact on the society and individual well-being of many Tanzanians. The research priority areas are explained below:

#### Research Priority 1: Determine Cardiovascular Disease Burden Data

One of the major problems with research in SSA has been lack of even basic data on disease burden (21, 22). This reality has led to most of estimated global burden of disease (GBD) data to utilize structural modeled estimates as a result of extremely limited information from some regions of the world, including SSA (22, 23). In Tanzania, the prevalence of most risk factors for CVD are known; namely hypertension (25.9%), diabetes (9.1%), elevated cholesterol (26%) as well as lifestyle conditions like overweight and obesity (26%) and cigarette smoking (15.9%) (4). However, there is still limited data on the community disease burden of specific CVD like heart failure, stroke, renal diseases, rheumatic heart disease, atrial fibrillation, cardiomyopathies, and others. Furthermore, disease trends including incidence, survival, and long-term outcome of CVD are unknown. Community burden of disease is important in order to plan for prioritization as well as targeted community interventions. Implementing this agenda will also facilitate reliable data to be used in the GBD studies related to CVD, which is important to place the country’s CVD burden into the global perspective. The plan is to prioritize community surveys of specific CVD in order to fill in the missing numbers of disease burden.

##### Action Points

• List and prioritize unmet needs for community CVD burden, especially those included in the GBD study
• Liaise with different stakeholders, including global funding agencies for collaboration and funding of research
• Create new, and organize existing population-based community cohorts on CVD to capture disease incidence and trends
• Conduct community surveys on CVD, prevalence, incidence and outcomes.

#### Research Priority 2: Conduct Cardiovascular Disease Prevention and Public Health Promotion Research

For most CVD, many important biological risk factors such as age, blood pressure, cholesterol levels and the significant influence of lifestyle and other environmental factors are well known, due to the seminal work from large follow-up cohort studies such as the Framingham Heart Study (24). However, efforts to reduce CVD risk by improving lifestyle are not working well enough in many countries (25) including Tanzania (4). Furthermore, even the knowledge of common risk factors is not universally understood among people in our communities (26, 27). There is therefore a knowledge gap in Tanzania in this area. This calls for rigorous multi-disciplinary CVD prevention research, involving social scientists, policy makers, and importantly involvement of the general Tanzanian population from the outset. As a starting point, there is a need for up-to-date and detailed data on population knowledge, current practice and health seeking behaviors toward CVD, as well as understanding of the best strategies (i.e., what works and what does not) to be used for an effective behavioral change toward CVD.

##### Action Points

• Define areas of community awareness needs
• Involve multi-stakeholders’ team on prevention research
• Conduct Knowledge, Attitude and Practice (KAP) studies on CVD
• Conduct implementation research on best strategies to attain behavior change toward CVD prevention.

#### Research Priority 3: Research on Early Detection and Management of Cardiovascular Disease

Most lifestyle-related CVD take long before they manifest with symptoms, making it possible for effective early interventions (28). However, early detection of CVD has been a challenge in most SSA countries (6). In Tanzania, most patients with CVD present late, often with acute events (29) or end organ damage when they first show up at healthcare facilities (30, 31). Tanzania, and other SSA countries can learn from developed countries, where improvement on early detection of CVD and their risk factors have made possible for many people to be on treatment or on the watch-list to prevent future cardiovascular events (32). This has been found to be more cost-effective in dealing with CVD as opposed to treating acute events and their complications (33, 34). A significant area of unmet need in the Tanzanian context is therefore to detect signs of CVD risk earlier enough, and to adapt the health system to be able to manage the populations at risk more effectively.

Research in this agenda will focus on investigating the applicability and cost-effectiveness of active early detection of CVD and their risk factors as well as studies on unique approaches to treatment of early disease including use of primary health facilities to treat CVD risk factors like hypertension, diabetes, and obesity. Also important in Tanzania, is early detection of acute rheumatic fever and rheumatic heart disease, as well as recognition of cardiac manifestations of tuberculosis, HIV and sickle cell anemia, among others.

##### Action Points

• Define list of important signs of early CVD
• Liaise with policy makers on referral of patients with early CVD
• Involve multi-disciplinary team of researchers to conduct research on early CVD
• Conduct implementation and cost-effectiveness research on early detection of CVD.

#### Research Priority 4: Establishing Disease Specific Registries for Cardiovascular Disease

Disease registries are a collection of patients’ secondary data, usually for a specific disease. The importance of disease registries cannot be overstated. In the GBD outcome data on CVD, SSA did not perform well and mortality projections using structural models were used instead of real mortality data due to limited or unreliable mortality data from the region (1). In developed countries, disease registries have brought in a wealth of information of specific disease trajectory, risk factors for complications including areas for individualized treatment (35). Furthermore, many research questions can be answered through accumulated data, answering questions on the course of disease, understanding variations in treatment and outcomes, examining factors that influence prognosis, as well as assessing the quality of life and care patterns among patients (36). Through functionalities such as feedback of data, registries are also increasingly been used to study quality improvement (37).

In SSA, disease registries have recently been developed and are increasing in number. They include single-country (38, 39) as well as an increasing number of regional registries (40, 41). However, funding to maintain and produce the required reliable data may be an issue, especially if it is not constant, as previously reported (42). In Tanzania, there are currently no formal systematic specific disease registries for CVD, although small short registries have been conducted mainly as single-center research (13). This research agenda will align existing CVD registries (in-country, regional), but more important will seek to create sustainable systems and infrastructure to create CVD registries of importance in Tanzania.

##### Action Points

• Define priority diseases for registry
• Obtain legal framework for continuous data collection and use
• Obtain patient groups involvement in registry keeping
• Obtain government support to initiate and maintain disease registries
• Strengthen Information Technology (IT) infrastructure for continuous data capturing and storage.

#### Research Priority 5: Harnessing the Power of Big Data in Hospital Day to Day Records

Currently, the amount of electronic data generated in healthcare worldwide is immense. Already, in developed countries the concepts of Big Data and a learning health care system have been a fast-growing area of medicine (43). Big Data refers to large and diverse sets of patients’ clinical information that is dynamic, and encompasses volumes of information, velocity or speed at which they are generated and collected, as well as the variety or scope of the data collected (43). In developed countries, Big Data is already used to produce useful information including translational and precision medicine research (44). Due to its diverse nature, Big Data analyses could inform government policy and therefore bringing about efficient patient care.

In many SSA countries, the introduction of computer-based patient records is new, but has taken effect and more countries have started to use patient electronic health management information systems (HIMS) (45, 46). This creates a unique opportunity for continuous data capturing, which can be translated into research. In Tanzania, Information Communication and Technology (ICT) plays a major role in healthcare management services, and since 2003 the government of Tanzania introduced the use of HIMS in all tertiary healthcare facilities and plans are underway to make most facilities in Tanzania use HIMS (47, 48). Currently, the amount of data generated in healthcare in Tanzania is therefore already immense, but the data is still fragmented, unstructured, and difficult to access for analytic purposes (49). This results in a data-to-innovation gap in health research. This agenda plans to research on best strategies that will result in adaptation of the FAIR (Findable, Accessible, Interoperable, and Re-usable) CVD data collection principles in tertiary university teaching health facilities in Tanzania, which can in the future be rolled-out to other health facilities in the country.

##### Action Points

• Improve data entry by health personnel through education and implementation research
• Obtain legal framework for continuous data collection and use
• Obtain patient groups involvement in continuous data collection
• Obtain government support to strengthen IT infrastructure
• Conduct implementation research to demonstrate the feasibility and evaluating impact of Big Data collection including cost effectiveness studies.

#### Research Priority 6: Research on Genetics of Cardiovascular Disease

Knowledge regarding genetic contribution on disease has gained significant attention in the past decades, and is an area of active research (50). In CVD medicine, major advances including the complex interplay between genetics and environmental factors in CVD causation have been appreciated. Large scale genome wide studies (GWAS) have been successful in uncovering novel susceptibility loci for a wide range of complex human diseases including hypertension, diabetes, cardiomyopathies, coronary artery disease, congenital heart disease, dyslipidaemia, arrhythmias, and others (51, 52). These advances are paving way for future management, including the possibility of precision medicine and new treatment options. Much of research in genetics of CVD has however been done in High Income Countries (HICs) of North America and Europe (51, 52), although other emerging economies like China are increasing the output of genetic research in CVD (53).

For SSA, genetic research is still in its infancy, but efforts to contribute to the world genetic research are rising (54). Availability of genetic screening tools and application of precision medicine for people of SSA will need genetic studies from SSA. There is therefore a need for active participation in genetic research; as genetic findings from one ethnic group do not wholly translate to another ethnic group. In South Africa, for example, it was recently shown that a gene panel with a yield of >60% in a European population has a yield of only 18% in South Africans with hypertrophic cardiomyopathy (55). In Tanzania, much of the reports on genetic studies have been on sickle cell anemia and malaria (56, 57), as well as on-going research on rare disease conditions. This agenda will focus to put in place infrastructure in terms of resources and human capacity build-up to be able to host a wide range of genetic research on CVD.

##### Action Points

• Prepare legal framework for genetic research and data collection
• Prepare patients’ involvement groups on genetic research
• Sensitize on volunteer programs for research
• Link–up with national, regional and international collaborators on CVD genetic research.

## Strategies for Sustainable Cardiovascular Disease Research Output: Challenges and Opportunities

### Strategy 1: Academia

Research undertaking requires human resources, in terms of number, expertise and dedication to lifelong research. In SSA, shortage of human resources for research is a well-recognized barrier to health research output from the region (58–61). The scarcity is in both the number of researchers, as well as the expertise (58, 59). Brain-drain, where young professionals emigrate to HICs for greener pastures (62) compounds the already scarce workforce in the region (63). Furthermore, while the culture of research is strongly embedded (64) and continuously been enhanced (65) in many developed countries, the same is not the case in SSA, where even the career path for a researcher is not well-defined (63). Competing interest with clinical work-load, engagement in private practice for economic reasons and un-interesting research questions (mainly conceived in Western countries), are some of the factors found to influence less interests in research among academic and non-academic health personnel in SSA (63, 66). However, with an increasing number of health care professionals reaching tertiary level education in the region (67), and increasing evidence that Africa-led research undertaking is possible (61) and an effective way to address local health challenges (68), this trend can be changed.

**FIGURE 2:**
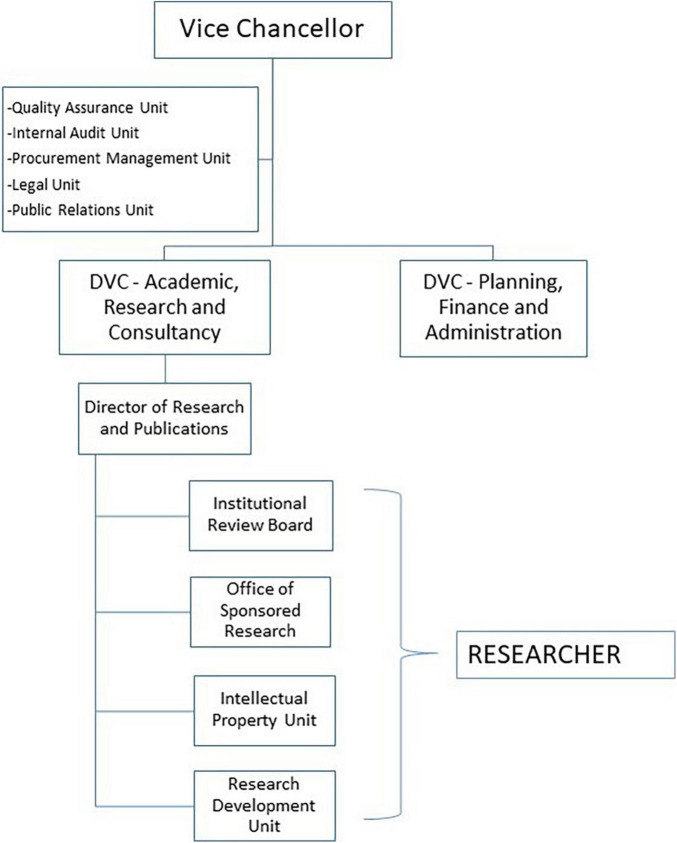
Research management structures within MUHAS. DVC, deputy vice-chancellor.

**FIGURE 3:**
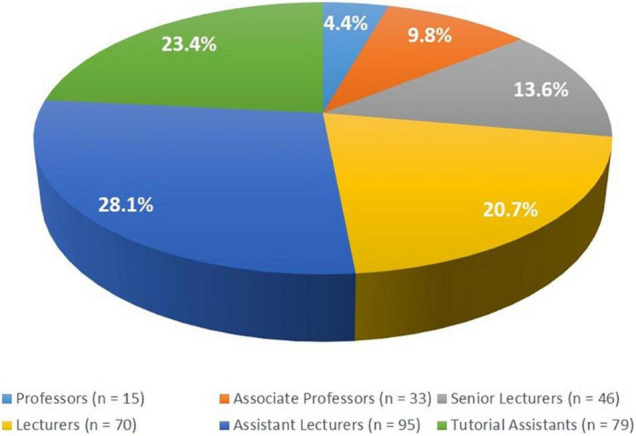
Distribution of MUHAS faculty by academic ranks.

This research agenda will strategically be hosted, implemented, and managed through the existing MUHAS research management systems (Figure 2) and through MUHAS academic programs, its faculty and post-graduate trainees (69). Academic institutions worldwide have traditionally been in the forefront for research, utilizing the dynamic learning environment within them, and the prospect for continuity. The current MUHAS academic profile provides a strong base for current and future research undertakings, taking advantage of relatively many young faculty at the institution (Figure 3).

Furthermore, the scope of researchers for this agenda is not limited to MUHAS as more researchers through collaboration with MUHAS faculty are expected to form part. Here the strategy will be to utilize the existing network of regional and global collaborators in research and faculty/students exchange.

#### Opportunities

• Higher proportion of young faculty at MUHAS
• Existing programs for CVD specialties that require research (dissertation) in CVD
• Existing researchers in CVD
• Increased popularity of CVD research within MUHAS
• Increased inter-disciplinary research collaboration within MUHAS faculty
• Large network of regional and global collaborators.

#### Challenges

• There are no specific CVD research training programs at MUHAS
• Very few existing faculty with skills for some areas of CVD research like basic sciences and genetic studies.

#### Mitigations

• Through the EACoECVS, CVD research training programs will be developed in order to specifically train and create a cadre of researchers in cardiovascular sciences in Tanzania and the East African region.
• Purposely, young faculty will be mentored and trained to develop interest and skills for genetic studies, while taking advantage of knowledge and skills from external collaborators.

### Strategy 2: Training

The low level of CVD research output from SSA is partly due to the low number of CVD researchers in the region (58, 59, 61, 63, 70). Furthermore, even with the available numbers, without proper research training, researchers from SSA lack expertise to conduct high caliber research. Proper research training is also a way of creating a culture of research, which has not been a well invested area in most SSA countries (61, 63, 71). Therefore, in order to increase the quantity and quality of research into CVD, there is a need in SSA, to urgently address two issues, i.e., increasing the number of researchers as well as improving their quality in order to produce researchers that will be professionals with in-depth scientific expertise and complementary leadership skills to enable them conduct independent, and internationally recognized CVD research.

Until now MUHAS, through the EACoECVS has made significant progress in increasing CVD specialties’ training (Table 1). Most of the initial training took place abroad including India, South Africa, Germany, United Kingdom, and Ireland as well as locally in Tanzania. This group of specialists in different CVD fields forms a base for current and future CVD researchers for the center. The strategy is to align these future researchers with the planned research agenda for the center.

**TABLE 1:** Trainees of the EACoECVS.

S/N	Specialty	Number trained
1	Public health specialist (MPH)	1
2	Public health specialist (Ph.D.)	2
3	Adult cardiology	3
4	Pediatrics cardiology	2
5	Cardiothoracic surgery	2
6	Clinical pharmacist	3
7	Cardiovascular intensivist	2
8	Cardiac catheterization laboratory nurse	2
9	Cardiac theater nurse	2
10	Cardiovascular intensive care nurse	2
11	Cardiovascular perfusionist	2
12	Biomedical engineering (in CV engineering)	3
13	Cardiovascular radiologist	3
14	Cardiovascular anesthesiologist	5
15	Cardiovascular technician	2
16	Cardiovascular physiologist	1
17	MSc data scientist	1

As part of research training at advanced levels, the EACoECVS has entered into memorandum of understanding with the University Medical Center Utrecht (UMCU) for tertiary CVD research training at the UMCU, through joint Ph.D. supervisions between UMCU and MUHAS. In this initiative, currently five Ph.D. fellows from MUHAS have already been registered with the Julius Global Health Ph.D. program and are doing their research in CVD (Table 2). Participants for this program receive all their formal research training in the Netherlands (both physically and through e-learning platforms), including research methods, epidemiology as well as scientific research writing. All research is done in Tanzania, following the research agenda highlighted.

**TABLE 2:** On-going joint Ph.D. supervision between MUHAS and UMCU.

S/N	Area of research	On-going Ph.d. (topic)
1.	Cardiomyopathy/genetics	Phenotypic, genotypic, and familial characterization of idiopathic dilated cardiomyopathy in a native Tanzanian cohort
2.	Rheumatic heart disease	Histopathomorphological and clinical findings of rheumatic mitral valve stenosis patients in Tanzania
3.	Congenital heart disease/registry	New-born screening for congenital heart diseases; profile and risk factors assessment in a tertiary care center; Tanzania
4.	Preventive cardiology/public health	Metabolic syndrome: the role of diet, lifestyle, and gut microbiota in rural and urban residents in Tanzania
5.	Cardiovascular pharmacology	Factors influencing warfarin dose response variability among Tanzanian patients with cardiovascular diseases on long term warfarin therapy

#### Opportunities

• Presence of the EACoECVS
• Existing programs for CVD training at MUHAS
• Existing collaborators in CVD research
• Already there is a base of young researchers in CVD at MUHAS.

#### Challenges

• There are still few faculty to conduct CVD research training at MUHAS.

#### Mitigations

• Continue to work with outside collaborators in CVD research training.

### Strategy 3: Infrastructure

Research infrastructure refers to facilities that provide resources, spaces and services for conducting research and foster innovation. In medicine these facilities include dedicated research laboratories, space for clinical trials, major scientific equipment, data repositories and archives, computing systems, and communication networks. Systems for supporting research management including quality assurance, monitoring and evaluation systems, financial systems, and physical space for researchers like faculty and scholars’ offices and lecture rooms are also part and parcel of a viable research environment. In SSA, both physical space and dedicated equipment for research have been in chronic shortage, and have been cited as major stumbling blocks for research undertaking in the region (63, 66, 68). This is further compounded by limited research organization at institutional, country or regional level (59). Over-reliance on infrastructure donation often brings lack of continuity when a certain project or research seizes to operate, leaving expensive machines unused.

**FIGURE 4:**
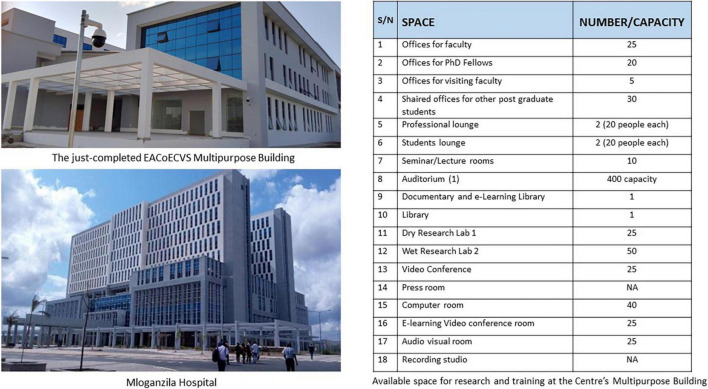
Available training and research infrastructure within the EACoECVS.

For this research agenda, most of the physical space will be through the deliberately built and strategically placed multipurpose building for the EACoECVS (Figure 4). This building is located at the new MUHAS Campus (approximately 3,800 acres about 25 km on the outskirts of Dar es Salaam city), and is close to the Mloganzila Hospital, a state-of-the art University teaching hospital for MUHAS. The construction of the multipurpose building for the EACoECVS was completed on 31 December 2021, and is expected to be fully functional in June 2022.

#### Opportunities

• Availability of adequate physical space within the newly built EACoECVS multipurpose building
• Huge space for expansion to include other research facilities
• Existing and functional infrastructure for research within MUHAS
• Existence of functioning/running research laboratories at MUHAS
• Existence of memoranda of understanding for research and clinical services with major public referral hospitals in Tanzania, including the Jakaya Kikwete Cardiac Institute, Muhimbili National Hospital, and Muhimbili – Mloganzila Hospital.

#### Challenges

• Still, there is insufficient collaboration in CVD research within universities/research organizations within Tanzania and East Africa.

#### Mitigations

• As custodian for CVD research and training for East Africa, the EACoECVS will organize collaborations between all existing CVD research centers within East Africa as mandated by the wider East African Centres of Excellences for Biomedical Sciences Project.

### Strategy 4: Funding

Funding is the backbone of any research endeavor. In developed countries, funding for research is a common practice and has been the driver for major breakthrough in health sciences (72). However, for many SSA countries the culture to fund research is lacking and brings about major challenges (63, 66, 71). Low social economic status of many SSA with competing budget priorities is undeniably the most important factor for poor research funding. However, other factors cited include lack of good governance, corruption and mismanagement of public funds at the higher levels (71). A review of research funding shows many researchers in SSA either self-fund their research or utilize post graduate small-scale dissertation funds to conduct research (63). Recently however, different initiatives have been implemented to facilitate SSA to access global health research funds, mainly by partnering with researchers from developed countries (73). Furthermore, access to other international funders like Bill and Melinda Gates Foundation, the Wellcome Trust, HIV Research Trust, The U.S. President’s Emergency Plan for AIDS Relief (PEPFAR), and others have been instrumental to funding research in SSA, mostly in infectious diseases (74). Only recently, funding for NCDs have emerged, although still very insufficient and with stiff competition (75).

This research agenda will not be practical without funding. As custodian of cardiovascular research in Tanzania, efforts to access cardiovascular research funds will be upgraded by equipping current and future generation researchers with skills to write competitive research grants. In publishing this agenda, potential collaborators for research from around the globe are being reached out, for the purpose of forming collaborations regionally and worldwide in order to attain sharing of research resources for a common goal. Locally, efforts to solicit research funding and support from government and non-governmental organizations will continue. This strategy will also involve reaching out to private partners, local philanthropists, businessmen and women as well as industries interested in cardiovascular research.

#### Opportunities

• Availability of competitive international funding organs for CVD research
• Increased global awareness of CVD as important developmental challenge.

#### Challenges

• Lack of research funding from national budget
• Less competitive advantages in proposal writing due to limited skills/expertise
• Expensive research undertakings
• Normally, donor-driven research questions
• Post-donor funded project discontinuation.

#### Mitigations

• Enforce efforts to solicit research funds through training, lobbying and involvement of the private sector.

## Conclusion

Despite many challenges, there are opportunities that can be taken advantage of, for a sustainable CVD research in Tanzania; the greatest opportunity being the evolving EACoECVS. Training of current and future faculty to take lead in CVD research will have a lasting legacy in this area, and will help to address the existing gap in establishment of locally informed CVD management and policy changes.

The high-level commitment in addressing the CVD burden shown by the Tanzanian Government can be taken as a lesson to other SSA countries that are yet to take decisive actions. In setting this research agenda we believe the research community from other SSA countries will learn and benchmark from it in prioritizing research areas, mobilizing resources and training of CVD researchers in the region.

## Author Contributions

PC, FM, and GK conceptualized the idea. PC performed literature search and wrote the first draft of the manuscript. FM, GK, PR, and AK critically reviewed the manuscript. All authors contributed to the manuscript and approved the final version.

## Conflict of Interest

The authors declare that the research was conducted in the absence of any commercial or financial relationships that could be construed as a potential conflict of interest.

## Publisher’s Note

All claims expressed in this article are solely those of the authors and do not necessarily represent those of their affiliated organizations, or those of the publisher, the editors and the reviewers. Any product that may be evaluated in this article, or claim that may be made by its manufacturer, is not guaranteed or endorsed by the publisher.
