# Supplementary material for: Developing a Sustainable Cardiovascular Disease Research Strategy in Tanzania Through Training: Leveraging From the East African Centre of Excellence in Cardiovascular Sciences Project
Source: Front Cardiovasc Med. 2022 Mar 25;9:849007. doi: 10.3389/fcvm.2022.849007 (PMC8990919; doi:10.3389/fcvm.2022.849007)
Supplement: Supplementary file 1 [file Data_Sheet_1.DOCX]

**History and Search Details**

Top of Form

Download

Bottom of Form

Delete

| **Search** | **Actions** | **Details** | **Query** | **Results** | **Time** |
| --- | --- | --- | --- | --- | --- |
| #13 |  |  | Search: **(#5) AND (#12)** | [196](https://pubmed.ncbi.nlm.nih.gov/?term=%28%235%29+AND+%28%2312%29&sort=relevance) | 14:16:59 |
| #12 |  |  | Search: **united republic of tanzania[MeSH Terms]** | [11,467](https://pubmed.ncbi.nlm.nih.gov/?term=united+republic+of+tanzania%5BMeSH+Terms%5D&sort=relevance) | 14:15:45 |
| #11 |  |  | Search: **(#5) AND (#9)** | [5,064](https://pubmed.ncbi.nlm.nih.gov/?term=%28%235%29+AND+%28%239%29&sort=relevance) | 14:14:38 |
| #10 |  |  | Search: **(# 5) AND (#9)** | [0](https://pubmed.ncbi.nlm.nih.gov/?term=%28%23+5%29+AND+%28%239%29&sort=relevance) | 14:13:38 |
| #9 |  |  | Search: **africa, sub saharan[MeSH Terms]** Filters: **from 2000 - 2021** | [142,395](https://pubmed.ncbi.nlm.nih.gov/?term=africa%2C+sub+saharan%5BMeSH+Terms%5D&filter=years.2000-2021&sort=relevance) | 14:12:23 |
| #8 |  |  | Search: **africa, sub saharan[MeSH Terms]** Filters: **from 1993 - 2021** | [162,505](https://pubmed.ncbi.nlm.nih.gov/?term=africa%2C+sub+saharan%5BMeSH+Terms%5D&filter=years.1993-2021&sort=relevance) | 14:12:12 |
| #7 |  |  | Search: **africa, sub saharan[MeSH Terms]** Filters: **from 1973 - 2021** | [198,286](https://pubmed.ncbi.nlm.nih.gov/?term=africa%2C+sub+saharan%5BMeSH+Terms%5D&filter=years.1973-2021&sort=relevance) | 14:12:02 |
| #6 |  |  | Search: **africa, sub saharan[MeSH Terms]** | [212,032](https://pubmed.ncbi.nlm.nih.gov/?term=africa%2C+sub+saharan%5BMeSH+Terms%5D&sort=relevance) | 14:11:50 |
| #5 |  |  | Search: **cardiovascular diseases[MeSH Terms]** Filters: **from 2000 - 2021** | [1,355,322](https://pubmed.ncbi.nlm.nih.gov/?term=cardiovascular+diseases%5BMeSH+Terms%5D&filter=years.2000-2021&sort=relevance) | 14:10:18 |
| #4 |  |  | Search: **cardiovascular diseases[MeSH Terms]** Filters: **from 1997 - 2021** | [1,475,409](https://pubmed.ncbi.nlm.nih.gov/?term=cardiovascular+diseases%5BMeSH+Terms%5D&filter=years.1997-2021&sort=relevance) | 14:10:12 |
| #3 |  |  | Search: **cardiovascular diseases[MeSH Terms]** Filters: **from 1984 - 2021** | [1,892,650](https://pubmed.ncbi.nlm.nih.gov/?term=cardiovascular+diseases%5BMeSH+Terms%5D&filter=years.1984-2021&sort=relevance) | 14:10:05 |
| #2 |  |  | Search: **cardiovascular diseases[MeSH Terms]** Filters: **from 1969 - 2021** | [2,207,722](https://pubmed.ncbi.nlm.nih.gov/?term=cardiovascular+diseases%5BMeSH+Terms%5D&filter=years.1969-2021&sort=relevance) | 14:09:55 |
| #1 |  |  | Search: **cardiovascular diseases[MeSH Terms]** | [2,405,346](https://pubmed.ncbi.nlm.nih.gov/?term=cardiovascular+diseases%5BMeSH+Terms%5D&sort=relevance) | 14:09:40 |

**History and Search Details**

Top of Form

Download

Bottom of Form

Delete

| **Search** | **Actions** | **Details** | **Query** | **Results** | **Time** |
| --- | --- | --- | --- | --- | --- |
| #5 |  |  | Search: **(#2) AND (#4)** | [681](https://pubmed.ncbi.nlm.nih.gov/?term=%28%232%29+AND+%28%234%29&sort=relevance) | 07:02:29 |
| #4 |  |  | Search: **((((kenya[MeSH Terms]) OR (rwanda[MeSH Terms])) OR (burundi[MeSH Terms])) OR (uganda[MeSH Terms])) OR (tanzania[MeSH Terms])** Filters: **from 2000 - 2021** | [30,014](https://pubmed.ncbi.nlm.nih.gov/?term=%28%28%28%28kenya%5BMeSH+Terms%5D%29+OR+%28rwanda%5BMeSH+Terms%5D%29%29+OR+%28burundi%5BMeSH+Terms%5D%29%29+OR+%28uganda%5BMeSH+Terms%5D%29%29+OR+%28tanzania%5BMeSH+Terms%5D%29&filter=years.2000-2021&sort=relevance) | 07:01:36 |
| #3 |  |  | Search: **((((kenya[MeSH Terms]) OR (rwanda[MeSH Terms])) OR (burundi[MeSH Terms])) OR (uganda[MeSH Terms])) OR (tanzania[MeSH Terms])** | [41,773](https://pubmed.ncbi.nlm.nih.gov/?term=%28%28%28%28kenya%5BMeSH+Terms%5D%29+OR+%28rwanda%5BMeSH+Terms%5D%29%29+OR+%28burundi%5BMeSH+Terms%5D%29%29+OR+%28uganda%5BMeSH+Terms%5D%29%29+OR+%28tanzania%5BMeSH+Terms%5D%29&sort=relevance) | 07:01:22 |
| #2 |  |  | Search: **cardiovascular diseases[MeSH Terms]** Filters: **from 2000 - 2021** | [1,363,279](https://pubmed.ncbi.nlm.nih.gov/?term=cardiovascular+diseases%5BMeSH+Terms%5D&filter=years.2000-2021&sort=relevance) | 06:59:51 |
| #1 |  |  | Search: **cardiovascular diseases[MeSH Terms]** | [2,413,303](https://pubmed.ncbi.nlm.nih.gov/?term=cardiovascular+diseases%5BMeSH+Terms%5D&sort=relevance) | 06:59:37 |

Showing 1 to 5 of 5 entries

**Cardiovascular Research East Africa: Total 681 articles (PubMed)**

1: Kavishe B, Vanobberghen F, Katende D, Kapiga S, Munderi P, Baisley K, Biraro

S, Mosha N, Mutungi G, Mghamba J, Hughes P, Smeeth L, Grosskurth H, Peck R.

Dyslipidemias and cardiovascular risk scores in urban and rural populations in

north-western Tanzania and southern Uganda. PLoS One. 2019 Dec

6;14(12):e0223189. doi: 10.1371/journal.pone.0223189. PMID: 31809516; PMCID:

PMC6897412.

2: WHO CVD Risk Chart Working Group. World Health Organization cardiovascular

disease risk charts: revised models to estimate risk in 21 global regions.

Lancet Glob Health. 2019 Oct;7(10):e1332-e1345. doi:

10.1016/S2214-109X(19)30318-3. Epub 2019 Sep 2. PMID: 31488387; PMCID:

PMC7025029.

3: Hertz JT, Sakita FM, Manavalan P, Madut DB, Thielman NM, Mmbaga BT, Staton

CA, Galson SW. The Burden of Hypertension and Diabetes in an Emergency

Department in Northern Tanzania. Ethn Dis. 2019 Oct 17;29(4):559-566. doi:

10.18865/ed.29.4.559. PMID: 31641323; PMCID: PMC6802168.

4: Musinguzi G, Wanyenze RK, Ndejjo R, Ssinabulya I, van Marwijk H, Ddumba I,

Bastiaens H, Nuwaha F. An implementation science study to enhance cardiovascular

disease prevention in Mukono and Buikwe districts in Uganda: a stepped-wedge

design. BMC Health Serv Res. 2019 Apr 25;19(1):253. doi:

10.1186/s12913-019-4095-0. PMID: 31023311; PMCID: PMC6482572.

5: Asiki G, Newton R, Marions L, Kamali A, Smedman L. The effect of childhood

stunting and wasting on adolescent cardiovascular diseases risk and educational

achievement in rural Uganda: a retrospective cohort study. Glob Health Action.

2019;12(1):1626184. doi: 10.1080/16549716.2019.1626184. PMID: 31232215; PMCID:

PMC6598535.

6: Chang H, Hawley NL, Kalyesubula R, Siddharthan T, Checkley W, Knauf F, Rabin

TL. Challenges to hypertension and diabetes management in rural Uganda: a

qualitative study with patients, village health team members, and health care

professionals. Int J Equity Health. 2019 Feb 28;18(1):38. doi:

10.1186/s12939-019-0934-1. PMID: 30819193; PMCID: PMC6394065.

7: Osoti AO, Page ST, Richardson BA, Guthrie BL, Kinuthia J, Polyak SJ, Farquhar

C. Postpartum metabolic syndrome after gestational hypertension and

preeclampsia, a prospective cohort study. Pregnancy Hypertens. 2019

Oct;18:35-41. doi: 10.1016/j.preghy.2019.08.088. Epub 2019 Aug 19. PMID:

31493627; PMCID: PMC6884686.

8: Msoka TF, Van Guilder GP, Smulders YM, van Furth M, Bartlett JA, van Agtmael

MA. Association of HIV-infection, antiretroviral treatment and metabolic

syndrome with large artery stiffness: a cross-sectional study. BMC Infect Dis.

2018 Dec 29;18(1):708. doi: 10.1186/s12879-018-3637-0. Erratum in: BMC Infect

Dis. 2019 Feb 22;19(1):187. PMID: 30594160; PMCID: PMC6311066.

9: Muiru AN, Bibangambah P, Hemphill L, Sentongo R, Kim JH, Triant VA, Bangsberg

DR, Tsai AC, Martin JN, Haberer JE, Boum Y 2nd, Plutzky J, Hunt PW, Okello S,

Siedner MJ. Distribution and Performance of Cardiovascular Risk Scores in a

Mixed Population of HIV-Infected and Community-Based HIV-Uninfected Individuals

in Uganda. J Acquir Immune Defic Syndr. 2018 Aug 1;78(4):458-464. doi:

10.1097/QAI.0000000000001696. PMID: 29652762; PMCID: PMC6019157.

10: Onyango EM, Onyango BM. The Rise of Noncommunicable Diseases in Kenya: An

Examination of the Time Trends and Contribution of the Changes in Diet and

Physical Inactivity. J Epidemiol Glob Health. 2018 Dec;8(1-2):1-7. doi:

10.2991/j.jegh.2017.11.004. PMID: 30859780; PMCID: PMC7325816.

11: Chiwanga FS, Njelekela MA, Diamond MB, Bajunirwe F, Guwatudde D, Nankya-

Mutyoba J, Kalyesubula R, Adebamowo C, Ajayi I, Reid TG, Volmink J, Laurence C,

Adami HO, Holmes MD, Dalal S. Urban and rural prevalence of diabetes and pre-

diabetes and risk factors associated with diabetes in Tanzania and Uganda. Glob

Health Action. 2016 May 23;9:31440. doi: 10.3402/gha.v9.31440. PMID: 27221531;

PMCID: PMC4879179.

12: Kwarisiima D, Atukunda M, Owaraganise A, Chamie G, Clark T, Kabami J, Jain

V, Byonanebye D, Mwangwa F, Balzer LB, Charlebois E, Kamya MR, Petersen M,

Havlir DV, Brown LB. Hypertension control in integrated HIV and chronic disease

clinics in Uganda in the SEARCH study. BMC Public Health. 2019 May 6;19(1):511.

doi: 10.1186/s12889-019-6838-6. PMID: 31060545; PMCID: PMC6501396.

13: Nsanya MK, Kavishe BB, Katende D, Mosha N, Hansen C, Nsubuga RN, Munderi P,

Grosskurth H, Kapiga S. Prevalence of high blood pressure and associated factors

among adolescents and young people in Tanzania and Uganda. J Clin Hypertens

(Greenwich). 2019 Apr;21(4):470-478. doi: 10.1111/jch.13502. Epub 2019 Feb 27.

PMID: 30811099.

14: Wagura P, Wasunna A, Laving A, Wamalwa D, Ng'ang'a P. Prevalence and factors

associated with preterm birth at kenyatta national hospital. BMC Pregnancy

Childbirth. 2018 Apr 19;18(1):107. doi: 10.1186/s12884-018-1740-2. PMID:

29673331; PMCID: PMC5909235.

15: Raphael DM, Roos L, Myovela V, Mchomvu E, Namamba J, Kilindimo S, Gingo W,

Hatz C, Paris DH, Weisser M, Kobza R, Rohacek M. Heart diseases and

echocardiography in rural Tanzania: Occurrence, characteristics, and etiologies

of underappreciated cardiac pathologies. PLoS One. 2018 Dec 26;13(12):e0208931.

doi: 10.1371/journal.pone.0208931. PMID: 30586432; PMCID: PMC6306243.

16: Kingery JR, Alfred Y, Smart LR, Nash E, Todd J, Naguib MR, Downs JA,

Kalluvya S, Kataraihya JB, Peck RN. Short-term and long-term cardiovascular

risk, metabolic syndrome and HIV in Tanzania. Heart. 2016 Aug 1;102(15):1200-5.

doi: 10.1136/heartjnl-2015-309026. Epub 2016 Apr 22. PMID: 27105648; PMCID:

PMC4945369.

17: Kaduka L, Korir A, Oduor CO, Kwasa J, Mbui J, Wabwire S, Gakunga R, Okerosi

N, Opanga Y, Kisiang'ani I, Chepkurui MR, Muniu E, Remick SC. Stroke

distribution patterns and characteristics in Kenya's leading public health

tertiary institutions: Kenyatta National Hospital and Moi Teaching and Referral

Hospital. Cardiovasc J Afr. 2018 Mar/Apr;29(2):68-72. doi:

10.5830/CVJA-2017-046. PMID: 29745965; PMCID: PMC6008906.

18: Asiki G, Shao S, Wainana C, Khayeka-Wandabwa C, Haregu TN, Juma PA, Mohammed

S, Wambui D, Gong E, Yan LL, Kyobutungi C. Policy environment for prevention,

control and management of cardiovascular diseases in primary health care in

Kenya. BMC Health Serv Res. 2018 May 9;18(1):344. doi:

10.1186/s12913-018-3152-4. PMID: 29743083; PMCID: PMC5944159.

19: Vedanthan R, Kamano JH, DeLong AK, Naanyu V, Binanay CA, Bloomfield GS,

Chrysanthopoulou SA, Finkelstein EA, Hogan JW, Horowitz CR, Inui TS, Menya D,

Orango V, Velazquez EJ, Were MC, Kimaiyo S, Fuster V. Community Health Workers

Improve Linkage to Hypertension Care in Western Kenya. J Am Coll Cardiol. 2019

Oct 15;74(15):1897-1906. doi: 10.1016/j.jacc.2019.08.003. Epub 2019 Sep 2. PMID:

31487546; PMCID: PMC6788970.

20: Hodel NC, Hamad A, Praehauser C, Mwangoka G, Kasella IM, Reither K, Abdulla

S, Hatz CFR, Mayr M. The epidemiology of chronic kidney disease and the

association with non-communicable and communicable disorders in a population of

sub-Saharan Africa. PLoS One. 2018 Oct 31;13(10):e0205326. doi:

10.1371/journal.pone.0205326. PMID: 30379902; PMCID: PMC6209178.

21: Ondimu DO, Kikuvi GM, Otieno WN. Risk factors for hypertension among young

adults (18-35) years attending in Tenwek Mission Hospital, Bomet County, Kenya

in 2018. Pan Afr Med J. 2019 Jul 16;33:210. doi:

10.11604/pamj.2019.33.210.18407. PMID: 31692887; PMCID: PMC6814951.

22: Osetinsky B, Hontelez JAC, Lurie MN, McGarvey ST, Bloomfield GS, Pastakia

SD, Wamai R, Bärnighausen T, de Vlas SJ, Galárraga O. Epidemiological And Health

Systems Implications Of Evolving HIV And Hypertension In South Africa And Kenya.

Health Aff (Millwood). 2019 Jul;38(7):1173-1181. doi:

10.1377/hlthaff.2018.05287. PMID: 31260360.

23: DeWyer A, Scheel A, Otim IO, Longenecker CT, Okello E, Ssinabulya I, Morris

S, Okwir M, Oyang W, Joyce E, Nabongo B, Sable C, Alencherry B, Tompsett A,

Aliku T, Beaton A. Improving the accuracy of heart failure diagnosis in low-

resource settings through task sharing and decentralization. Glob Health Action.

2019;12(1):1684070. doi: 10.1080/16549716.2019.1684070. PMID: 31694487; PMCID:

PMC6844369.

24: Mboera LEG, Rumisha SF, Lyimo EP, Chiduo MG, Mangu CD, Mremi IR, Kumalija

CJ, Joachim C, Kishamawe C, Massawe IS, Matemba LE, Kimario E, Bwana VM,

Mkwashapi DM. Cause-specific mortality patterns among hospital deaths in

Tanzania, 2006-2015. PLoS One. 2018 Oct 31;13(10):e0205833. doi:

10.1371/journal.pone.0205833. PMID: 30379899; PMCID: PMC6209209.

25: Muhihi AJ, Urassa DP, Mpembeni RNM, Leyna GH, Sunguya BF, Kakoko D, Kessy

AT, Njelekela MA. Effect of training community health workers and their

interventions on cardiovascular disease risk factors among adults in Morogoro,

Tanzania: study protocol for a cluster randomized controlled trial. Trials. 2018

Oct 11;19(1):552. doi: 10.1186/s13063-018-2924-9. PMID: 30314511; PMCID:

PMC6186034.

26: Kija EN, Saunders DE, Munubhi E, Darekar A, Barker S, Cox TCS, Mango M, Soka

D, Komba J, Nkya DA, Cox SE, Kirkham FJ, Newton CRJC. Transcranial Doppler and

Magnetic Resonance in Tanzanian Children With Sickle Cell Disease. Stroke. 2019

Jul;50(7):1719-1726. doi: 10.1161/STROKEAHA.118.018920. Epub 2019 Jun 14. PMID:

31195937; PMCID: PMC6594727.

27: Babua C, Kalyesubula R, Okello E, Kakande B, Sebatta E, Mungoma M, Mondo C.

Pattern and presentation of cardiac diseases among patients with chronic kidney

disease attending a national referral hospital in Uganda: a cross sectional

study. BMC Nephrol. 2015 Aug 4;16:126. doi: 10.1186/s12882-015-0128-z. PMID:

26238594; PMCID: PMC4522958.

28: Mohamed SF, Mutua MK, Wamai R, Wekesah F, Haregu T, Juma P, Nyanjau L,

Kyobutungi C, Ogola E. Prevalence, awareness, treatment and control of

hypertension and their determinants: results from a national survey in Kenya.

BMC Public Health. 2018 Nov 7;18(Suppl 3):1219. doi: 10.1186/s12889-018-6052-y.

PMID: 30400858; PMCID: PMC6219055.

29: Nnko S, Bukenya D, Kavishe BB, Biraro S, Peck R, Kapiga S, Grosskurth H,

Seeley J. Chronic Diseases in North-West Tanzania and Southern Uganda. Public

Perceptions of Terminologies, Aetiologies, Symptoms and Preferred Management.

PLoS One. 2015 Nov 10;10(11):e0142194. doi: 10.1371/journal.pone.0142194. PMID:

26555896; PMCID: PMC4640879.

30: Kibirige D, Atuhe D, Kampiire L, Kiggundu DS, Donggo P, Nabbaale J, Mwebaze

RM, Kalyesubula R, Lumu W. Access to medicines and diagnostic tests integral in

the management of diabetes mellitus and cardiovascular diseases in Uganda:

insights from the ACCODAD study. Int J Equity Health. 2017 Aug 24;16(1):154.

doi: 10.1186/s12939-017-0651-6. PMID: 28836972; PMCID: PMC5571570.

31: Makubi A, Hage C, Sartipy U, Lwakatare J, Janabi M, Kisenge P, Dahlström U,

Rydén L, Makani J, Lund LH. Heart failure in Tanzania and Sweden: Comparative

characterization and prognosis in the Tanzania Heart Failure (TaHeF) study and

the Swedish Heart Failure Registry (SwedeHF). Int J Cardiol. 2016 Oct

1;220:750-8. doi: 10.1016/j.ijcard.2016.06.239. Epub 2016 Jun 29. PMID:

27393861; PMCID: PMC5553107.

32: Stockdale L, Nash S, Nalwoga A, Painter H, Asiki G, Fletcher H, Newton R.

Human cytomegalovirus epidemiology and relationship to tuberculosis and

cardiovascular disease risk factors in a rural Ugandan cohort. PLoS One. 2018

Feb 6;13(2):e0192086. doi: 10.1371/journal.pone.0192086. PMID: 29408860; PMCID:

PMC5800673.

33: Engelgau MM. Tackling High Blood Pressure in Kenya and Other Low- and

Middle-Income Countries: Why Now and What Can We Do? Glob Heart. 2019

Mar;14(1):71-73. doi: 10.1016/j.gheart.2019.03.002. PMID: 31036304.

34: Juma K, Nyabera R, Mbugua S, Odinya G, Jowi J, Ngunga M, Zakus D, Yonga G.

Cardiovascular risk factors among people living with HIV in rural Kenya: a

clinic-based study. Cardiovasc J Afr. 2019 Jan/Feb 23;30(1):52-56. doi:

10.5830/CVJA-2018-064. Epub 2019 Jan 24. PMID: 30720846.

35: Magodoro IM, Feng M, North CM, Vořechovská D, Kraemer JD, Kakuhikire B,

Bangsberg D, Tsai AC, Siedner MJ. Female sex and cardiovascular disease risk in

rural Uganda: a cross-sectional, population-based study. BMC Cardiovasc Disord.

2019 Apr 25;19(1):96. doi: 10.1186/s12872-019-1072-9. PMID: 31023227; PMCID:

PMC6485175.

36: Mondo CK, Otim MA, Akol G, Musoke R, Orem J. The prevalence and distribution

of non-communicable diseases and their risk factors in Kasese district, Uganda.

Cardiovasc J Afr. 2013 Apr;24(3):52-7. doi: 10.5830/CVJA-2012-081. PMID:

23736126; PMCID: PMC3721879.

37: Longenecker CT, Kalra A, Okello E, Lwabi P, Omagino JO, Kityo C, Kamya MR,

Webel AR, Simon DI, Salata RA, Costa MA. A Human-Centered Approach to CV Care:

Infrastructure Development in Uganda. Glob Heart. 2018 Dec;13(4):347-354. doi:

10.1016/j.gheart.2018.02.002. Epub 2018 Apr 21. PMID: 29685638; PMCID:

PMC6258347.

38: Magodoro IM, Albano AJ, Muthalaly R, Koplan B, North CM, Vořechovská D,

Downey J, Kraemer J, Vaglio M, Badilini F, Kakuhire B, Tsai AC, Siedner MJ.

Population Prevalence and Correlates of Prolonged QT Interval: Cross-Sectional,

Population-Based Study From Rural Uganda. Glob Heart. 2019 Mar;14(1):17-25.e4.

doi: 10.1016/j.gheart.2018.11.002. Epub 2018 Dec 21. PMID: 30584028; PMCID:

PMC6737252.

39: Sibomana JP, McNamara RL, Walker TD. Patient, clinician and logistic

barriers to blood pressure control among adult hypertensives in rural district

hospitals in Rwanda: a cross-sectional study. BMC Cardiovasc Disord. 2019 Oct

21;19(1):231. doi: 10.1186/s12872-019-1203-3. PMID: 31638907; PMCID: PMC6805529.

40: Mwangi N, Gachago M, Gichangi M, Gichuhi S, Githeko K, Jalango A, Karimurio

J, Kibachio J, Muthami L, Ngugi N, Nduri C, Nyaga P, Nyamori J, Zindamoyen ANM,

Bascaran C, Foster A. Adapting clinical practice guidelines for diabetic

retinopathy in Kenya: process and outputs. Implement Sci. 2018 Jun 15;13(1):81.

doi: 10.1186/s13012-018-0773-2. PMID: 29903039; PMCID: PMC6003001.

41: Lajeunesse-Trempe F, Dufour R, du Souich P, Paquette M, Kaduka LU,

Christensen DL. Anthropometric measures and their association with risk factors

for cardio-metabolic diseases in Kenyan adults. Ann Hum Biol. 2018 Sep-

Dec;45(6-8):486-495. doi: 10.1080/03014460.2018.1562568. PMID: 30608195.

42: Njelekela M, Sato T, Nara Y, Miki T, Kuga S, Noguchi T, Kanda T, Yamori M,

Ntogwisangu J, Masesa Z, Mashalla Y, Mtabaji J, Yamori Y. Nutritional variation

and cardiovascular risk factors in Tanzania--rural-urban difference. S Afr Med

J. 2003 Apr;93(4):295-9. PMID: 12806724.

43: Moloi AH, Mall S, Engel ME, Stafford R, Zhu ZW, Zühlke LJ, Watkins DA. The

Health Systems Barriers and Facilitators for RHD Prevalence: An Epidemiological

Meta-Analysis From Uganda and Tanzania. Glob Heart. 2017 Mar;12(1):5-15.e3. doi:

10.1016/j.gheart.2016.12.002. Epub 2017 Mar 14. PMID: 28302546.

44: Mashili F, Joachim A, Aboud S, Mchembe M, Chiwanga F, Addo J, Kendall L, Ako

A, Abbas Z. Prospective exploration of the effect of adiposity and associated

microbial factors on healing and progression of diabetic foot ulcers in

Tanzania: study protocol of a longitudinal cohort study. BMJ Open. 2019 Dec

16;9(12):e031896. doi: 10.1136/bmjopen-2019-031896. PMID: 31848165; PMCID:

PMC6937068.

45: Raichlen DA, Pontzer H, Harris JA, Mabulla AZ, Marlowe FW, Josh Snodgrass J,

Eick G, Colette Berbesque J, Sancilio A, Wood BM. Physical activity patterns and

biomarkers of cardiovascular disease risk in hunter-gatherers. Am J Hum Biol.

2017 Mar;29(2). doi: 10.1002/ajhb.22919. Epub 2016 Oct 9. PMID: 27723159.

46: Musinguzi G, Bastiaens H, Wanyenze RK, Mukose A, Van Geertruyden JP, Nuwaha

F. Capacity of Health Facilities to Manage Hypertension in Mukono and Buikwe

Districts in Uganda: Challenges and Recommendations. PLoS One. 2015 Nov

11;10(11):e0142312. doi: 10.1371/journal.pone.0142312. PMID: 26560131; PMCID:

PMC4641641.

47: Mugure G, Karama M, Kyobutungi C, Karanja S. Correlates for cardiovascular

diseases among diabetic/hypertensive patients attending outreach clinics in two

Nairobi slums, Kenya. Pan Afr Med J. 2014 Nov 10;19:261. doi:

10.11604/pamj.2014.19.261.5261. PMID: 25852804; PMCID: PMC4382055.

48: Barasa FA, Vedanthan R, Pastakia SD, Crowe SJ, Aruasa W, Sugut WK, White R,

Ogola ES, Bloomfield GS, Velazquez EJ. Approaches to Sustainable Capacity

Building for Cardiovascular Disease Care in Kenya. Cardiol Clin. 2017

Feb;35(1):145-152. doi: 10.1016/j.ccl.2016.08.014. PMID: 27886785.

49: Rachlis B, Naanyu V, Wachira J, Genberg B, Koech B, Kamene R, Akinyi J,

Braitstein P. Community Perceptions of Community Health Workers (CHWs) and Their

Roles in Management for HIV, Tuberculosis and Hypertension in Western Kenya.

PLoS One. 2016 Feb 22;11(2):e0149412. doi: 10.1371/journal.pone.0149412. PMID:

26901854; PMCID: PMC4764025.

50: Ngalesoni F, Ruhago G, Mayige M, Oliveira TC, Robberstad B, Norheim OF,

Higashi H. Cost-effectiveness analysis of population-based tobacco control

strategies in the prevention of cardiovascular diseases in Tanzania. PLoS One.

2017 Aug 2;12(8):e0182113. doi: 10.1371/journal.pone.0182113. PMID: 28767722;

PMCID: PMC5540531.

51: Mannik J, Figol A, Churchill V, Aw J, Francis S, Karino E, Chesire JK, Opot

D, Ochieng B, Hawkes MT. Community-based screening for cardiovascular risk using

a novel mHealth tool in rural Kenya. J Innov Health Inform. 2018 Oct

31;25(3):176-182. doi: 10.14236/jhi.v25i3.1012. PMID: 30398461.

52: Zuechner A, Mhada T, Majani NG, Sharau GG, Mahalu W, Freund MW. Spectrum of

heart diseases in children presenting to a paediatric cardiac echocardiography

clinic in the Lake Zone of Tanzania: a 7 years overview. BMC Cardiovasc Disord.

2019 Dec 13;19(1):291. doi: 10.1186/s12872-019-01292-4. PMID: 31835996; PMCID:

PMC6909619.

53: Nkusi AE, Muneza S, Nshuti S, Hakizimana D, Munyemana P, Nkeshimana M,

Rudakemwa E, Amendezo E. Stroke Burden in Rwanda: A Multicenter Study of Stroke

Management and Outcome. World Neurosurg. 2017 Oct;106:462-469. doi:

10.1016/j.wneu.2017.06.163. Epub 2017 Jul 8. PMID: 28698086.

54: Wekesah FM, Kyobutungi C, Grobbee DE, Klipstein-Grobusch K. Understanding of

and perceptions towards cardiovascular diseases and their risk factors: a

qualitative study among residents of urban informal settings in Nairobi. BMJ

Open. 2019 Jun 16;9(6):e026852. doi: 10.1136/bmjopen-2018-026852. PMID:

31209088; PMCID: PMC6588962.

55: Nyombi KV, Kizito S, Mukunya D, Nabukalu A, Bukama M, Lunyera J, Asiimwe M,

Kimuli I, Kalyesubula R. High prevalence of hypertension and cardiovascular

disease risk factors among medical students at Makerere University College of

Health Sciences, Kampala, Uganda. BMC Res Notes. 2016 Feb 17;9:110. doi:

10.1186/s13104-016-1924-7. PMID: 26887672; PMCID: PMC4756514.

56: Subramanian S, Hilscher R, Gakunga R, Munoz B, Ogola E. Cost-effectiveness

of risk stratified medication management for reducing premature cardiovascular

mortality in Kenya. PLoS One. 2019 Jun 25;14(6):e0218256. doi:

10.1371/journal.pone.0218256. Erratum in: PLoS One. 2020 May 7;15(5):e0233139.

PMID: 31237910; PMCID: PMC6592597.

57: Lunyera J, Kirenga B, Stanifer JW, Kasozi S, van der Molen T, Katagira W,

Kamya MR, Kalyesubula R. Geographic differences in the prevalence of

hypertension in Uganda: Results of a national epidemiological study. PLoS One.

2018 Aug 1;13(8):e0201001. doi: 10.1371/journal.pone.0201001. PMID: 30067823;

PMCID: PMC6070243.

58: Baumann AA, Mutabazi V, Brown AL, Hooley C, Reeds D, Ingabire C, Ndahindwa

V, Nishimwe A, Cade WT, de Las Fuentes L, Proctor EK, Karengera S, Schecthman

KB, Goss CW, Yarasheski K, Newsome B, Mutimura E, Davila-Roman VG. Dissemination

and Implementation Program in Hypertension in Rwanda: Report on Initial Training

and Evaluation. Glob Heart. 2019 Jun;14(2):135-141. doi:

10.1016/j.gheart.2019.06.001. PMID: 31324367; PMCID: PMC6816501.

59: Riha J, Karabarinde A, Ssenyomo G, Allender S, Asiki G, Kamali A, Young EH,

Sandhu MS, Seeley J. Urbanicity and lifestyle risk factors for cardiometabolic

diseases in rural Uganda: a cross-sectional study. PLoS Med. 2014 Jul

29;11(7):e1001683. doi: 10.1371/journal.pmed.1001683. PMID: 25072243; PMCID:

PMC4114555.

60: Ngalesoni FN, Ruhago GM, Mori AT, Robberstad B, Norheim OF. Equity impact

analysis of medical approaches to cardiovascular diseases prevention in

Tanzania. Soc Sci Med. 2016 Dec;170:208-217. doi:

10.1016/j.socscimed.2016.08.033. Epub 2016 Aug 24. PMID: 27590271.

61: Muhihi AJ, Njelekela MA, Mpembeni RNM, Muhihi BG, Anaeli A, Chillo O,

Kubhoja S, Lujani B, Maghembe M, Ngarashi D. Elevated blood pressure among

primary school children in Dar es salaam, Tanzania: prevalence and risk factors.

BMC Pediatr. 2018 Feb 13;18(1):54. doi: 10.1186/s12887-018-1052-8. PMID:

29433455; PMCID: PMC5809963.

62: Semakula JR, Mouton JP, Jorgensen A, Hutchinson C, Allie S, Semakula L,

French N, Lamorde M, Toh CH, Blockman M, Sekaggya-Wiltshire C, Waitt C,

Pirmohamed M, Cohen K. A cross-sectional evaluation of five warfarin

anticoagulation services in Uganda and South Africa. PLoS One. 2020 Jan

29;15(1):e0227458. doi: 10.1371/journal.pone.0227458. PMID: 31995565; PMCID:

PMC6988943.

63: Okello E, Longenecker CT, Beaton A, Kamya MR, Lwabi P. Rheumatic heart

disease in Uganda: predictors of morbidity and mortality one year after

presentation. BMC Cardiovasc Disord. 2017 Jan 7;17(1):20. doi:

10.1186/s12872-016-0451-8. PMID: 28061759; PMCID: PMC5219796.

64: Feinstein MJ, Kim JH, Bibangambah P, Sentongo R, Martin JN, Tsai AC,

Bangsberg DR, Hemphill L, Triant VA, Boum Y 2nd, Hunt PW, Okello S, Siedner MJ.

Ideal Cardiovascular Health and Carotid Atherosclerosis in a Mixed Cohort of

HIV-Infected and Uninfected Ugandans. AIDS Res Hum Retroviruses. 2017

Jan;33(1):49-56. doi: 10.1089/AID.2016.0104. Epub 2016 Sep 7. PMID: 27476547;

PMCID: PMC5240009.

65: Nahimana MR, Nyandwi A, Muhimpundu MA, Olu O, Condo JU, Rusanganwa A, Koama

JB, Ngoc CT, Gasherebuka JB, Ota MO, Okeibunor JC. A population-based national

estimate of the prevalence and risk factors associated with hypertension in

Rwanda: implications for prevention and control. BMC Public Health. 2017 Jul

10;18(1):2. doi: 10.1186/s12889-017-4536-9. Erratum in: BMC Public Health. 2017

Sep 22;17 (1):736. PMID: 28693458; PMCID: PMC5504833.

66: Bintabara D, Mpondo BCT. Preparedness of lower-level health facilities and

the associated factors for the outpatient primary care of hypertension: Evidence

from Tanzanian national survey. PLoS One. 2018 Feb 15;13(2):e0192942. doi:

10.1371/journal.pone.0192942. PMID: 29447231; PMCID: PMC5814020.

67: Pallangyo P, Fredrick F, Bhalia S, Nicholaus P, Kisenge P, Mtinangi B,

Janabi M, Humphrey S. Cardiorenal Anemia Syndrome and Survival among Heart

Failure Patients in Tanzania: A Prospective Cohort Study. BMC Cardiovasc Disord.

2017 Feb 14;17(1):59. doi: 10.1186/s12872-017-0497-2. PMID: 28193165; PMCID:

PMC5307834.

68: Muhihi A, Njelekela M, Mpembeni R, Masesa Z, Kitamori K, Mori M, Kato N,

Mtabaji J, Yamori Y. Physical activity and cardiovascular disease risk factors

among young and middle-aged men in urban Mwanza, Tanzania. Pan Afr Med J.

2012;11:11. Epub 2012 Jan 20. PMID: 22368754; PMCID: PMC3283018.

69: Kaduka L, Muniu E, Mbui J, Oduor Owuor C, Gakunga R, Kwasa J, Wabwire S,

Okerosi N, Korir A, Remick SC. Disability-Adjusted Life-Years Due to Stroke in

Kenya. Neuroepidemiology. 2019;53(1-2):48-54. doi: 10.1159/000498970. Epub 2019

Apr 15. PMID: 30986786.

70: Ettarh R, Van de Vijver S, Oti S, Kyobutungi C. Overweight, obesity, and

perception of body image among slum residents in Nairobi, Kenya, 2008-2009. Prev

Chronic Dis. 2013 Dec 19;10:E212. doi: 10.5888/pcd10.130198. PMID: 24355105;

PMCID: PMC3869529.

71: Hertz JT, Madut DB, Tesha RA, William G, Simmons RA, Galson SW, Sakita FM,

Maro VP, Bloomfield GS, Crump JA, Rubach MP. Knowledge of myocardial infarction

symptoms and perceptions of self-risk in Tanzania. Am Heart J. 2019

Apr;210:69-74. doi: 10.1016/j.ahj.2019.01.003. Epub 2019 Jan 15. PMID: 30743209;

PMCID: PMC6453561.

72: Temu TM, Lane KA, Shen C, Ng'ang'a L, Akwanalo CO, Chen PS, Emonyi W,

Heckbert SR, Koech MM, Manji I, Vatta M, Velazquez EJ, Wessel J, Kimaiyo S, Inui

TS, Bloomfield GS. Clinical characteristics and 12-month outcomes of patients

with valvular and non-valvular atrial fibrillation in Kenya. PLoS One. 2017 Sep

21;12(9):e0185204. doi: 10.1371/journal.pone.0185204. PMID: 28934312; PMCID:

PMC5608343.

73: Mkuu RS, Gilreath TD, Wekullo C, Reyes GA, Harvey IS. Social determinants of

hypertension and type-2 diabetes in Kenya: A latent class analysis of a

nationally representative sample. PLoS One. 2019 Aug 19;14(8):e0221257. doi:

10.1371/journal.pone.0221257. PMID: 31425539; PMCID: PMC6699668.

74: Chang AY, Nabbaale J, Nalubwama H, Okello E, Ssinabulya I, Longenecker CT,

Webel AR. Motivations of women in Uganda living with rheumatic heart disease: A

mixed methods study of experiences in stigma, childbearing, anticoagulation, and

contraception. PLoS One. 2018 Mar 28;13(3):e0194030. doi:

10.1371/journal.pone.0194030. PMID: 29590159; PMCID: PMC5874006.

75: Mosha NR, Mahande M, Juma A, Mboya I, Peck R, Urassa M, Michael D, Todd J.

Prevalence,awareness and factors associated with hypertension in North West

Tanzania. Glob Health Action. 2017;10(1):1321279. doi:

10.1080/16549716.2017.1321279. PMID: 28598724; PMCID: PMC5496079.

76: Kaduka L, Muniu E, Oduor C, Mbui J, Gakunga R, Kwasa J, Wabwire S, Okerosi

N, Korir A, Remick S. Stroke Mortality in Kenya's Public Tertiary Hospitals: A

Prospective Facility-Based Study. Cerebrovasc Dis Extra. 2018;8(2):70-79. doi:

10.1159/000488205. Epub 2018 Jun 12. PMID: 29895000; PMCID: PMC6031945.

77: Sander LD, Newell K, Ssebbowa P, Serwadda D, Quinn TC, Gray RH, Wawer MJ,

Mondo G, Reynolds S. Hypertension, cardiovascular risk factors and

antihypertensive medication utilisation among HIV-infected individuals in Rakai,

Uganda. Trop Med Int Health. 2015 Mar;20(3):391-6. doi: 10.1111/tmi.12443. Epub

2014 Dec 26. PMID: 25430847; PMCID: PMC4308448.

78: Mathenge W, Foster A, Kuper H. Urbanization, ethnicity and cardiovascular

risk in a population in transition in Nakuru, Kenya: a population-based survey.

BMC Public Health. 2010 Sep 22;10:569. doi: 10.1186/1471-2458-10-569. PMID:

20860807; PMCID: PMC2956724.

79: Spies LA, Gray J, Opollo JG, Mbalinda S, Nabirye R, Asher CA.

Transformational leadership as a framework for nurse education about

hypertension in Uganda. Nurse Educ Today. 2018 May;64:172-174. doi:

10.1016/j.nedt.2018.02.009. Epub 2018 Feb 17. PMID: 29500997.

80: Horwitz S, Yogo J, Juma E, Ice GH. Caregiving and cardiovascular disease

risk factors in male and female Luo elders from Kenya. Ann Hum Biol. 2009 Jul-

Aug;36(4):400-10. doi: 10.1080/03014460902845720. Epub 2009 May 26. PMID:

19468933.

81: Kaddumukasa M, Kayima J, Kaddumukasa MN, Ddumba E, Mugenyi L, Pundik S,

Furlan AJ, Sajatovic M, Katabira E. Knowledge, attitudes and perceptions of

stroke: a cross-sectional survey in rural and urban Uganda. BMC Res Notes. 2015

Dec 26;8:819. doi: 10.1186/s13104-015-1820-6. PMID: 26708348; PMCID: PMC4691295.

82: Kavishe B, Biraro S, Baisley K, Vanobberghen F, Kapiga S, Munderi P, Smeeth

L, Peck R, Mghamba J, Mutungi G, Ikoona E, Levin J, Bou Monclús MA, Katende D,

Kisanga E, Hayes R, Grosskurth H. High prevalence of hypertension and of risk

factors for non-communicable diseases (NCDs): a population based cross-sectional

survey of NCDS and HIV infection in Northwestern Tanzania and Southern Uganda.

BMC Med. 2015 May 29;13:126. doi: 10.1186/s12916-015-0357-9. PMID: 26021319;

PMCID: PMC4476208.

83: Shao PJ, Sawe HR, Murray BL, Mfinanga JA, Mwafongo V, Runyon MS. Profile of

patients with hypertensive urgency and emergency presenting to an urban

emergency department of a tertiary referral hospital in Tanzania. BMC Cardiovasc

Disord. 2018 Aug 2;18(1):158. doi: 10.1186/s12872-018-0895-0. PMID: 30068315;

PMCID: PMC6090910.

84: Walker RW, McLarty DG, Kitange HM, Whiting D, Masuki G, Mtasiwa DM, Machibya

H, Unwin N, Alberti KG. Stroke mortality in urban and rural Tanzania. Adult

Morbidity and Mortality Project. Lancet. 2000 May 13;355(9216):1684-7. doi:

10.1016/s0140-6736(00)02240-6. PMID: 10905244.

85: Litorp H, Kidanto HL, Rööst M, Abeid M, Nyström L, Essén B. Maternal near-

miss and death and their association with caesarean section complications: a

cross-sectional study at a university hospital and a regional hospital in

Tanzania. BMC Pregnancy Childbirth. 2014 Jul 23;14:244. doi:

10.1186/1471-2393-14-244. PMID: 25056517; PMCID: PMC4133054.

86: Namukwaya E, Murray SA, Downing J, Leng M, Grant L. 'I think my body has

become addicted to those tablets'. Chronic heart failure patients' understanding

of and beliefs about their illness and its treatment: A qualitative longitudinal

study from Uganda. PLoS One. 2017 Sep 28;12(9):e0182876. doi:

10.1371/journal.pone.0182876. PMID: 28957338; PMCID: PMC5619713.

87: Muthalaly RG, Koplan BA, Albano A, North C, Campbell JI, Kakuhikire B,

Vořechovská D, Kraemer JD, Tsai AC, Siedner MJ. Low population prevalence of

atrial fibrillation in rural Uganda: A community-based cross-sectional study.

Int J Cardiol. 2018 Nov 15;271:87-91. doi: 10.1016/j.ijcard.2018.05.074. Epub

2018 May 24. PMID: 29859712; PMCID: PMC6143408.

88: Bloomfield GS, Wang TY, Boulware LE, Califf RM, Hernandez AF, Velazquez EJ,

Peterson ED, Li JS. Implementation of management strategies for diabetes and

hypertension: from local to global health in cardiovascular diseases. Glob

Heart. 2015 Mar;10(1):31-8. doi: 10.1016/j.gheart.2014.12.010. PMID: 25754564;

PMCID: PMC4754665.

89: Abeya FC, Lumori BAE, Akello SJ, Annex BH, Buda AJ, Okello S. Incidence and

predictors of 6 months mortality after an acute heart failure event in rural

Uganda: The Mbarara Heart Failure Registry (MAHFER). Int J Cardiol. 2018 Aug

1;264:113-117. doi: 10.1016/j.ijcard.2018.03.110. Epub 2018 Mar 29. PMID:

29655949; PMCID: PMC6743717.

90: Smith MT, Monahan MP, Nelson P, Moruzzi M, DeLucenay AJ, Birnie CR. Elevated

blood pressure in the developing world: a role for clinical pharmacists. Int J

Pharm Pract. 2018 Aug;26(4):334-340. doi: 10.1111/ijpp.12398. Epub 2017 Sep 19.

PMID: 28925056.

91: Rusingiza EK, El-Khatib Z, Hedt-Gauthier B, Ngoga G, Dusabeyezu S, Tapela N,

Mutumbira C, Mutabazi F, Harelimana E, Mucumbitsi J, Kwan GF, Bukhman G.

Outcomes for patients with rheumatic heart disease after cardiac surgery

followed at rural district hospitals in Rwanda. Heart. 2018

Oct;104(20):1707-1713. doi: 10.1136/heartjnl-2017-312644. Epub 2018 Apr 20.

PMID: 29678896; PMCID: PMC6173815.

92: Mugeni R, Nkusi E, Rutaganda E, Musafiri S, Masaisa F, Lewis KL, Simpao M,

Tugirimana PL, Walker TD. Proximal deep vein thrombosis among hospitalised

medical and obstetric patients in Rwandan university teaching hospitals:

prevalence and associated risk factors: a cross-sectional study. BMJ Open. 2019

Nov 25;9(11):e032604. doi: 10.1136/bmjopen-2019-032604. PMID: 31772101; PMCID:

PMC6887052.

93: Grimaldi A, Alfieri O, Camici PG, La Canna G, Zoppei G, Olivotto I. "Mal

d'Africa" e cuore: i misteri della fibrosi endomiocardica ["African sickness"

and the heart: the mystery of endomyocardial fibrosis]. G Ital Cardiol (Rome).

2011 Jul-Aug;12(7-8):484-91. Italian. doi: 10.1714/915.10074. PMID: 21779114.

94: Kwarisiima D, Balzer L, Heller D, Kotwani P, Chamie G, Clark T, Ayieko J,

Mwangwa F, Jain V, Byonanebye D, Petersen M, Havlir D, Kamya MR. Population-

Based Assessment of Hypertension Epidemiology and Risk Factors among HIV-

Positive and General Populations in Rural Uganda. PLoS One. 2016 May

27;11(5):e0156309. doi: 10.1371/journal.pone.0156309. PMID: 27232186; PMCID:

PMC4883789.

95: Kuper H, Mathenge W, Macleod D, Foster A, Gichangi M, Rono H, Wing K, Weiss

HA, Bastawrous A, Burton M. Mortality during 6 years of follow-up in relation to

visual impairment and eye disease: results from a population-based cohort study

of people aged 50 years and above in Nakuru, Kenya. BMJ Open. 2019 Jun

9;9(6):e029700. doi: 10.1136/bmjopen-2019-029700. PMID: 31182456; PMCID:

PMC6561440.

96: Ngalesoni F, Ruhago G, Norheim OF, Robberstad B. Economic cost of primary

prevention of cardiovascular diseases in Tanzania. Health Policy Plan. 2015

Sep;30(7):875-84. doi: 10.1093/heapol/czu088. Epub 2014 Aug 11. PMID: 25113027;

PMCID: PMC4524339.

97: Noorani M, Lakhani N. Kawasaki disease: two case reports from the Aga Khan

Hospital, Dar es Salaam-Tanzania. BMC Pediatr. 2018 Oct 23;18(1):334. doi:

10.1186/s12887-018-1306-5. PMID: 30352566; PMCID: PMC6198366.

98: Galson SW, Stanifer JW, Hertz JT, Temu G, Thielman N, Gafaar T, Staton CA.

The burden of hypertension in the emergency department and linkage to care: A

prospective cohort study in Tanzania. PLoS One. 2019 Jan 25;14(1):e0211287. doi:

10.1371/journal.pone.0211287. PMID: 30682173; PMCID: PMC6347227.

99: Biraguma J, Mutimura E, Frantz JM. Knowledge about modifiable risk factors

for non-communicable diseases adults living with HIV in Rwanda. Afr Health Sci.

2019 Dec;19(4):3181-3189. doi: 10.4314/ahs.v19i4.41. PMID: 32127895; PMCID:

PMC7040312.

100: Jones R, Putnam HWI, Philippin H, Cleland C, Steel DH, Gray WK, Klaptocz

JE, Swai B, Walker RW. Retinal imaging to identify target organ damage in older

Africans: A pilot study. J Clin Hypertens (Greenwich). 2018 Sep;20(9):1296-1301.

doi: 10.1111/jch.13352. Epub 2018 Jul 19. PMID: 30027598.

101: Bloomfield GS, Mwangi A, Chege P, Simiyu CJ, Aswa DF, Odhiambo D, Obala AA,

Ayuo P, Khwa-Otsyula BO. Multiple cardiovascular risk factors in Kenya: evidence

from a health and demographic surveillance system using the WHO STEPwise

approach to chronic disease risk factor surveillance. Heart. 2013

Sep;99(18):1323-9. doi: 10.1136/heartjnl-2013-303913. Epub 2013 Jul 19. PMID:

23872588; PMCID: PMC3898037.

102: Rwebembera J, Manyilirah W, Zhu ZW, Nabbaale J, Namuyonga J, Ssinabulya I,

Lubega S, Lwabi P, Omagino J, Okello E. Prevalence and characteristics of

primary left-sided valve disease in a cohort of 15,000 patients undergoing

echocardiography studies in a tertiary hospital in Uganda. BMC Cardiovasc

Disord. 2018 May 4;18(1):82. doi: 10.1186/s12872-018-0813-5. PMID: 29728065;

PMCID: PMC5935941.

103: Maher D, Waswa L, Baisley K, Karabarinde A, Unwin N, Grosskurth H.

Distribution of hyperglycaemia and related cardiovascular disease risk factors

in low-income countries: a cross-sectional population-based survey in rural

Uganda. Int J Epidemiol. 2011 Feb;40(1):160-71. doi: 10.1093/ije/dyq156. Epub

2010 Oct 5. PMID: 20926371; PMCID: PMC3043279.

104: Omuse G, Maina D, Hoffman M, Mwangi J, Wambua C, Kagotho E, Amayo A, Ojwang

P, Premji Z, Ichihara K, Erasmus R. Metabolic syndrome and its predictors in an

urban population in Kenya: A cross sectional study. BMC Endocr Disord. 2017 Jul

4;17(1):37. doi: 10.1186/s12902-017-0188-0. Erratum in: BMC Endocr Disord. 2017

Jul 14;17 (1):43. PMID: 28676116; PMCID: PMC5496352.

105: Maletnlema TN. A Tanzanian perspective on the nutrition transition and its

implications for health. Public Health Nutr. 2002 Feb;5(1A):163-8. doi:

10.1079/phn2001289. PMID: 12027280.

106: Ngunga LM, Yonga G, Wachira B, Ezekowitz JA. Initial Rhythm and

Resuscitation Outcomes for Patients Developing Cardiac Arrest in Hospital: Data

From Low-Middle Income Country. Glob Heart. 2018 Dec;13(4):255-260. doi:

10.1016/j.gheart.2018.07.001. Epub 2018 Aug 18. PMID: 30131253.

107: Beaton A, Okello E, Scheel A, DeWyer A, Ssembatya R, Baaka O, Namisanvu H,

Njeri A, Matovu A, Namagembe I, Mccarter R, Carapetis J, DeStigter K, Sable C.

Impact of heart disease on maternal, fetal and neonatal outcomes in a low-

resource setting. Heart. 2019 May;105(10):755-760. doi:

10.1136/heartjnl-2018-313810. Epub 2018 Nov 10. PMID: 30415203.

108: Chillo P, Humphrey SH, Meda J, Kerry VB. Cardiac critical care in resource-

limited environments: lessons from Tanzania. Glob Heart. 2014 Sep;9(3):311-8.

doi: 10.1016/j.gheart.2014.06.004. Epub 2014 Oct 31. PMID: 25667182.

109: Tumaini B, Munseri P, Pallangyo K. Disease spectrum and outcomes among

elderly patients in two tertiary hospitals in Dar es Salaam, Tanzania. PLoS One.

2019 Oct 10;14(10):e0213131. doi: 10.1371/journal.pone.0213131. PMID: 31600198;

PMCID: PMC6786655.

110: Vusirikala A, Wekesah F, Kyobutungi C, Oyebode O. Assessment of

cardiovascular risk in a slum population in Kenya: use of World Health

Organisation/International Society of Hypertension (WHO/ISH) risk prediction

charts - secondary analyses of a household survey. BMJ Open. 2019 Sep

4;9(9):e029304. doi: 10.1136/bmjopen-2019-029304. PMID: 31488481; PMCID:

PMC6731939.

111: Eberly LA, Rusingiza E, Park PH, Ngoga G, Dusabeyezu S, Mutabazi F,

Harerimana E, Mucumbitsi J, Nyembo PF, Borg R, Gahamanyi C, Mutumbira C,

Ntaganda E, Rusangwa C, Kwan GF, Bukhman G. Understanding the Etiology of Heart

Failure Among the Rural Poor in Sub-Saharan Africa: A 10-Year Experience From

District Hospitals in Rwanda. J Card Fail. 2018 Dec;24(12):849-853. doi:

10.1016/j.cardfail.2018.10.002. Epub 2018 Oct 10. PMID: 30312764.

112: Lunyera J, Stanifer JW, Ingabire P, Etolu W, Bagasha P, Egger JR, Patel UD,

Mutungi G, Kalyesubula R. Prevalence and correlates of proteinuria in Kampala,

Uganda: a cross-sectional pilot study. BMC Res Notes. 2016 Feb 16;9:97. doi:

10.1186/s13104-016-1897-6. PMID: 26879636; PMCID: PMC4755001.

113: Lule SA, Namara B, Akurut H, Lubyayi L, Nampijja M, Akello F, Tumusiime J,

Aujo JC, Oduru G, Mentzer AJ, Smeeth L, Elliott AM, Webb EL. Blood pressure risk

factors in early adolescents: results from a Ugandan birth cohort. J Hum

Hypertens. 2019 Sep;33(9):679-692. doi: 10.1038/s41371-019-0178-y. Epub 2019 Feb

25. PMID: 30804461; PMCID: PMC6760975.

114: Abdallah A, Chang JL, O'Carroll CB, Okello S, Olum S, Acan M, Aden AA, Chow

FC, Siedner MJ. Validation of the Intracerebral Hemorrhage Score in Uganda.

Stroke. 2018 Dec;49(12):3063-3066. doi: 10.1161/STROKEAHA.118.022057. PMID:

30571425; PMCID: PMC6309793.

115: Vedanthan R, Kamano JH, Bloomfield GS, Manji I, Pastakia S, Kimaiyo SN.

Engaging the Entire Care Cascade in Western Kenya: A Model to Achieve the

Cardiovascular Disease Secondary Prevention Roadmap Goals. Glob Heart. 2015

Dec;10(4):313-7. doi: 10.1016/j.gheart.2015.09.003. PMID: 26704963; PMCID:

PMC4691279.

116: Kuria N, Reid A, Owiti P, Tweya H, Kibet CK, Mbau L, Manzi M, Murunga V,

Namusonge T, Kibachio J. Compliance with follow-up and adherence to medication

in hypertensive patients in an urban informal settlement in Kenya: comparison of

three models of care. Trop Med Int Health. 2018 Jul;23(7):785-794. doi:

10.1111/tmi.13078. PMID: 29779264.

117: Ogola EN, Okello FO, Herr JL, Macgregor-Skinner E, Mulvaney A, Yonga G.

Healthy Heart Africa-Kenya: A 12-Month Prospective Evaluation of Program Impact

on Health Care Providers' Knowledge and Treatment of Hypertension. Glob Heart.

2019 Mar;14(1):61-70. doi: 10.1016/j.gheart.2019.02.002. PMID: 31036303.

118: Ogeng'o JA, Gatonga P, Olabu BO. Cardiovascular causes of death in an east

African country: an autopsy study. Cardiol J. 2011;18(1):67-72. PMID: 21305488.

119: Njelekela MA, Mpembeni R, Muhihi A, Mligiliche NL, Spiegelman D, Hertzmark

E, Liu E, Finkelstein JL, Fawzi WW, Willett WC, Mtabaji J. Gender-related

differences in the prevalence of cardiovascular disease risk factors and their

correlates in urban Tanzania. BMC Cardiovasc Disord. 2009 Jul 17;9:30. doi:

10.1186/1471-2261-9-30. PMID: 19615066; PMCID: PMC2723083.

120: Kiggundu DS, Mutebi E, Kibirige D, Boxer R, Kakande B, Kigozi BK, Katabira

E. Vitamin D deficiency and its characteristics among patients with acute stroke

at a national referral hospital in Kampala Uganda. BMC Endocr Disord. 2015 Oct

5;15:53. doi: 10.1186/s12902-015-0053-y. PMID: 26437924; PMCID: PMC4594645.

121: Muddu M, Mutebi E, Ssinabulya I, Kizito S, Mondo CK. Hypertension among

newly diagnosed diabetic patients at Mulago National Referral Hospital in

Uganda: a cross sectional study. Cardiovasc J Afr. 2018 Jul/Aug

23;29(4):218-224. doi: 10.5830/CVJA-2018-015. Epub 2018 Apr 20. PMID: 29750228.

122: Rodríguez-Arbolí E, Mwamelo K, Kalinjuma AV, Furrer H, Hatz C, Tanner M,

Battegay M, Letang E; KIULARCO Study Group. Incidence and risk factors for

hypertension among HIV patients in rural Tanzania - A prospective cohort study.

PLoS One. 2017 Mar 8;12(3):e0172089. doi: 10.1371/journal.pone.0172089. PMID:

28273105; PMCID: PMC5342176.

123: Makubi A, Mmbando BP, Novelli EM, Lwakatare J, Soka D, Marik H, Tibarazwa

K, Ngaeje M, Newton CR, Gladwin MT, Makani J. Rates and risk factors of

hypertension in adolescents and adults with sickle cell anaemia in Tanzania:

10 years' experience. Br J Haematol. 2017 Jun;177(6):930-937. doi:

10.1111/bjh.14330. Epub 2016 Sep 21. PMID: 27650269; PMCID: PMC5612392.

124: Kontchou NT, McCrary AW, Schulman KA. Workforce Cost Model for Expanding

Congenital and Rheumatic Heart Disease Services in Kenya. World J Pediatr

Congenit Heart Surg. 2019 May;10(3):321-327. doi: 10.1177/2150135119837201.

PMID: 31084310.

125: Barsky J, Hunter R, McAllister C, Yeates K, Campbell N, Liu P, Perkins N,

Hua-Stewart D, Maar MA, Tobe SW. Analysis of the Implementation, User

Perspectives, and Feedback From a Mobile Health Intervention for Individuals

Living With Hypertension (DREAM-GLOBAL): Mixed Methods Study. JMIR Mhealth

Uhealth. 2019 Dec 9;7(12):e12639. doi: 10.2196/12639. PMID: 31815678; PMCID:

PMC6928701.

126: Lynch HM, Green AS, Clarke Nanyonga R, Gadikota-Klumpers DD, Squires A,

Schwartz JI, Heller DJ. Exploring patient experiences with and attitudes towards

hypertension at a private hospital in Uganda: a qualitative study. Int J Equity

Health. 2019 Dec 30;18(1):206. doi: 10.1186/s12939-019-1109-9. PMID: 31888767;

PMCID: PMC6937689.

127: Etyang AO, Munge K, Bunyasi EW, Matata L, Ndila C, Kapesa S, Owiti M,

Khandwalla I, Brent AJ, Tsofa B, Kabibu P, Morpeth S, Bauni E, Otiende M, Ojal

J, Ayieko P, Knoll MD, Smeeth L, Williams TN, Griffiths UK, Scott JA. Burden of

disease in adults admitted to hospital in a rural region of coastal Kenya: an

analysis of data from linked clinical and demographic surveillance systems.

Lancet Glob Health. 2014 Apr;2(4):e216-24. doi: 10.1016/S2214-109X(14)70023-3.

PMID: 24782954; PMCID: PMC3986034.

128: Phillips-Howard PA, Laserson KF, Amek N, Beynon CM, Angell SY, Khagayi S,

Byass P, Hamel MJ, van Eijk AM, Zielinski-Gutierrez E, Slutsker L, De Cock KM,

Vulule J, Odhiambo FO. Deaths ascribed to non-communicable diseases among rural

Kenyan adults are proportionately increasing: evidence from a health and

demographic surveillance system, 2003-2010. PLoS One. 2014 Nov 26;9(11):e114010.

doi: 10.1371/journal.pone.0114010. PMID: 25426945; PMCID: PMC4245262.

129: Huffman MD, Rao KD, Pichon-Riviere A, Zhao D, Harikrishnan S, Ramaiya K,

Ajay VS, Goenka S, Calcagno JI, Caporale JE, Niu S, Li Y, Liu J, Thankappan KR,

Daivadanam M, van Esch J, Murphy A, Moran AE, Gaziano TA, Suhrcke M, Reddy KS,

Leeder S, Prabhakaran D. A cross-sectional study of the microeconomic impact of

cardiovascular disease hospitalization in four low- and middle-income countries.

PLoS One. 2011;6(6):e20821. doi: 10.1371/journal.pone.0020821. Epub 2011 Jun 14.

PMID: 21695127; PMCID: PMC3114849.

130: Mushengezi B, Chillo P. Association between body fat composition and blood

pressure level among secondary school adolescents in Dar es Salaam, Tanzania.

Pan Afr Med J. 2014 Nov 27;19:327. doi: 10.11604/pamj.2014.19.327.5222. PMID:

25918567; PMCID: PMC4405073.

131: Achwoka D, Waruru A, Chen TH, Masamaro K, Ngugi E, Kimani M, Mukui I, Oyugi

JO, Mutave R, Achia T, Katana A, Ng'ang'a L, De Cock KM. Noncommunicable disease

burden among HIV patients in care: a national retrospective longitudinal

analysis of HIV-treatment outcomes in Kenya, 2003-2013. BMC Public Health. 2019

Apr 3;19(1):372. doi: 10.1186/s12889-019-6716-2. PMID: 30943975; PMCID:

PMC6448214.

132: Hertz JT, Sakita FM, Manavalan P, Mmbaga BT, Thielman NM, Staton CA.

Knowledge, attitudes, and preventative practices regarding ischemic heart

disease among emergency department patients in northern Tanzania. Public Health.

2019 Oct;175:60-67. doi: 10.1016/j.puhe.2019.06.017. Epub 2019 Aug 8. PMID:

31401252; PMCID: PMC6791754.

133: Okello S, Ueda P, Kanyesigye M, Byaruhanga E, Kiyimba A, Amanyire G, Kintu

A, Fawzi WW, Muyindike WR, Danaei G. Association between HIV and blood pressure

in adults and role of body weight as a mediator: Cross-sectional study in

Uganda. J Clin Hypertens (Greenwich). 2017 Nov;19(11):1181-1191. doi:

10.1111/jch.13092. Epub 2017 Sep 12. PMID: 28895288; PMCID: PMC5693777.

134: Nagu T, Ray R, Munseri P, Moshiro C, Shayo G, Kazema R, Mugusi F, Pallangyo

K. Tuberculosis among the elderly in Tanzania: disease presentation and initial

response to treatment. Int J Tuberc Lung Dis. 2017 Dec 1;21(12):1251-1257. doi:

10.5588/ijtld.17.0161. PMID: 29297445.

135: Vancampfort D, Probst M, Basangwa D, De Hert M, Myin-Germeys I, van Winkel

R, Ward PB, Rosenbaum S, Mugisha J. Adherence to physical activity

recommendations and physical and mental health risk in people with severe mental

illness in Uganda. Psychiatry Res. 2018 Feb;260:236-240. doi:

10.1016/j.psychres.2017.11.067. Epub 2017 Nov 29. PMID: 29220680.

136: Mwita JC, Mugusi F, Lwakatare J, Chiwanga F. Hypertension control and other

cardiovascular risk factors among diabetic patients at Muhimbili National

Hospital, Tanzania. East Afr J Public Health. 2012 Jun;9(2):70-3. PMID:

23139960.

137: Msemo OA, Schmiegelow C, Nielsen BB, Kousholt H, Grunnet LG, Christensen

DL, Lusingu JPA, Møller SL, Kavishe RA, Minja DTR, Bygbjerg IC. Risk factors of

pre-hypertension and hypertension among non-pregnant women of reproductive age

in northeastern Tanzania: a community based cross-sectional study. Trop Med Int

Health. 2018 Nov;23(11):1176-1187. doi: 10.1111/tmi.13149. Epub 2018 Oct 2.

PMID: 30280462.

138: Kitya D, Punchak M, Abdelgadir J, Obiga O, Harborne D, Haglund MM. Causes,

clinical presentation, management, and outcomes of chronic subdural hematoma at

Mbarara Regional Referral Hospital. Neurosurg Focus. 2018 Oct;45(4):E7. doi:

10.3171/2018.7.FOCUS18253. PMID: 30269586.

139: Syed N, Rockers PC, Vian T, Onyango M, Laing RO, Wirtz VJ. Access to

Antihypertensive Medicines at the Household Level: A Study From 8 Counties of

Kenya. Glob Heart. 2018 Dec;13(4):247-253.e5. doi: 10.1016/j.gheart.2018.08.001.

Epub 2018 Sep 20. PMID: 30245176.

140: Mwanri AW, Kinabo JL, Ramaiya K, Feskens EJ. High blood pressure and

associated risk factors among women attending antenatal clinics in Tanzania. J

Hypertens. 2015 May;33(5):940-7. doi: 10.1097/HJH.0000000000000501. PMID:

25909697.

141: Osoti A, Temu TM, Kirui N, Ngetich EK, Kamano JH, Page S, Farquhar C,

Bloomfield GS. Metabolic Syndrome Among Antiretroviral Therapy-Naive Versus

Experienced HIV-Infected Patients Without Preexisting Cardiometabolic Disorders

in Western Kenya. AIDS Patient Care STDS. 2018 Jun;32(6):215-222. doi:

10.1089/apc.2018.0052. PMID: 29851503; PMCID: PMC5982154.

142: Carlson S, Duber HC, Achan J, Ikilezi G, Mokdad AH, Stergachis A, Wollum A,

Bukhman G, Roth GA. Capacity for diagnosis and treatment of heart failure in

sub-Saharan Africa. Heart. 2017 Dec;103(23):1874-1879. doi:

10.1136/heartjnl-2016-310913. Epub 2017 May 10. PMID: 28490619.

143: Ssinabulya I, Nabunnya Y, Kiggundu B, Musoke C, Mungoma M, Kayima J.

Hypertension control and care at Mulago Hospital ambulatory clinic, Kampala-

Uganda. BMC Res Notes. 2016 Nov 17;9(1):487. doi: 10.1186/s13104-016-2293-y.

PMID: 27855717; PMCID: PMC5114775.

144: Walker RW, Dewhurst M, Gray WK, Jusabani A, Aris E, Unwin N, Swai M, Adams

PC, Mugusi F. Electrocardiographic assessment of coronary artery disease and

stroke risk factors in rural and urban Tanzania: a case-control study. J Stroke

Cerebrovasc Dis. 2014 Feb;23(2):315-20. doi:

10.1016/j.jstrokecerebrovasdis.2013.03.002. Epub 2013 Mar 30. PMID: 23545320;

PMCID: PMC4185096.

145: Kaddumukasa M, Mugenyi L, Kaddumukasa MN, Ddumba E, Devereaux M, Furlan A,

Sajatovic M, Katabira E. Prevalence and incidence of neurological disorders

among adult Ugandans in rural and urban Mukono district; a cross-sectional

study. BMC Neurol. 2016 Nov 17;16(1):227. doi: 10.1186/s12883-016-0732-y. PMID:

27855635; PMCID: PMC5114749.

146: Hertz JT, Madut DB, William G, Maro VP, Crump JA, Rubach MP. Perceptions of

Stroke and Associated Health-Care-Seeking Behavior in Northern Tanzania: A

Community-Based Study. Neuroepidemiology. 2019;53(1-2):41-47. doi:

10.1159/000499069. Epub 2019 Apr 15. PMID: 30986785; PMCID: PMC6842572.

147: Nyangasa MA, Buck C, Kelm S, Sheikh MA, Brackmann KL, Hebestreit A.

Association between cardiometabolic risk factors and body mass index, waist

circumferences and body fat in a Zanzibari cross-sectional study. BMJ Open. 2019

Jul 4;9(7):e025397. doi: 10.1136/bmjopen-2018-025397. PMID: 31278089; PMCID:

PMC6615808.

148: Bahiru E, Temu T, Mwanga J, Ndede K, Vusha S, Gitura B, Farquhar C, Bukachi

F, Huffman MD. Facilitators, context of and barriers to acute coronary syndrome

care at Kenyatta National Hospital, Nairobi, Kenya: a qualitative analysis.

Cardiovasc J Afr. 2018 May/Jun 23;29(3):177-182. doi: 10.5830/CVJA-2018-013.

Epub 2018 Apr 20. PMID: 29750227; PMCID: PMC6107733.

149: Larson E, Rabkin M, Mbaruku GM, Mbatia R, Kruk ME. Missed Opportunities to

Improve the Health of Postpartum Women: High Rates of Untreated Hypertension in

Rural Tanzania. Matern Child Health J. 2017 Mar;21(3):407-413. doi:

10.1007/s10995-016-2229-0. PMID: 28120288; PMCID: PMC5357453.

150: Ominde BS, Ogeng'o JA, Misiani MK, Kariuki BN. Pattern of stroke in a rural

Kenyan hospital. Malawi Med J. 2019 Mar;31(1):50-55. doi: 10.4314/mmj.v31i1.9.

PMID: 31143397; PMCID: PMC6526339.

151: Heller DJ, Balzer LB, Kazi D, Charlebois ED, Kwarisiima D, Mwangwa F, Jain

V, Kotwani P, Chamie G, Cohen CR, Clark TD, Ayieko J, Byonanabye DM, Petersen M,

Kamya MR, Havlir D, Kahn JG. Hypertension testing and treatment in Uganda and

Kenya through the SEARCH study: An implementation fidelity and outcome

evaluation. PLoS One. 2020 Jan 15;15(1):e0222801. doi:

10.1371/journal.pone.0222801. PMID: 31940346; PMCID: PMC6961918.

152: van de Vijver S, Oti S, Moll van Charante E, Allender S, Foster C, Lange J,

Oldenburg B, Kyobutungi C, Agyemang C. Cardiovascular prevention model from

Kenyan slums to migrants in the Netherlands. Global Health. 2015 Mar 7;11:11.

doi: 10.1186/s12992-015-0095-y. PMID: 25890177; PMCID: PMC4363048.

153: Wandera SO, Kwagala B, Ntozi J. Prevalence and risk factors for self-

reported non-communicable diseases among older Ugandans: a cross-sectional

study. Glob Health Action. 2015 Jul 22;8:27923. doi: 10.3402/gha.v8.27923. PMID:

26205363; PMCID: PMC4513181.

154: Vedanthan R, Lee DJ, Kamano JH, Herasme OI, Kiptoo P, Tulienge D, Kimaiyo

S, Balasubramanian H, Fuster V. Hypertension management in rural western Kenya:

a needs-based health workforce estimation model. Hum Resour Health. 2019 Jul

16;17(1):57. doi: 10.1186/s12960-019-0389-x. PMID: 31311561; PMCID: PMC6636021.

155: Wamala JF, Karyabakabo Z, Ndungutse D, Guwatudde D. Prevalence factors

associated with hypertension in Rukungiri district, Uganda--a community-based

study. Afr Health Sci. 2009 Sep;9(3):153-60. PMID: 20589143; PMCID: PMC2887031.

156: Swain JD, Sinnott C, Breakey S, Hasson Charles R, Mody G, Nyirimanzi N,

Patton-Bolman C, Come P, Ganza G, Rusingiza E, Ruhamya N, Mucumbitsi J, Borges

J, Zammert M, Muehlschlegel JD, Oakes R, Leavitt B, Bolman RM 3rd. Ten-year

clinical experience of humanitarian cardiothoracic surgery in Rwanda: Building a

platform for ultimate sustainability in a resource-limited setting. J Thorac

Cardiovasc Surg. 2018 Jun;155(6):2541-2550. doi: 10.1016/j.jtcvs.2017.11.106.

Epub 2018 Feb 27. PMID: 29499865.

157: Ververs M, Muriithi JW, Burton A, Burton JW, Lawi AO. Scurvy Outbreak Among

South Sudanese Adolescents and Young Men - Kakuma Refugee Camp, Kenya,

2017-2018. MMWR Morb Mortal Wkly Rep. 2019 Jan 25;68(3):72-75. doi:

10.15585/mmwr.mm6803a4. PMID: 30677009; PMCID: PMC6348760.

158: Cavanagh P, Attinger C, Abbas Z, Bal A, Rojas N, Xu ZR. Cost of treating

diabetic foot ulcers in five different countries. Diabetes Metab Res Rev. 2012

Feb;28 Suppl 1:107-11. doi: 10.1002/dmrr.2245. PMID: 22271734.

159: Joshi MD, Ayah R, Njau EK, Wanjiru R, Kayima JK, Njeru EK, Mutai KK.

Prevalence of hypertension and associated cardiovascular risk factors in an

urban slum in Nairobi, Kenya: a population-based survey. BMC Public Health. 2014

Nov 18;14:1177. doi: 10.1186/1471-2458-14-1177. PMID: 25407513; PMCID:

PMC4246542.

160: Guwatudde D, Nankya-Mutyoba J, Kalyesubula R, Laurence C, Adebamowo C,

Ajayi I, Bajunirwe F, Njelekela M, Chiwanga FS, Reid T, Volmink J, Adami HO,

Holmes MD, Dalal S. The burden of hypertension in sub-Saharan Africa: a four-

country cross sectional study. BMC Public Health. 2015 Dec 5;15:1211. doi:

10.1186/s12889-015-2546-z. PMID: 26637309; PMCID: PMC4670543.

161: Maginga J, Guerrero M, Koh E, Holm Hansen C, Shedafa R, Kalokola F, Smart

LR, Peck RN. Hypertension Control and Its Correlates Among Adults Attending a

Hypertension Clinic in Tanzania. J Clin Hypertens (Greenwich). 2016

Mar;18(3):207-16. doi: 10.1111/jch.12646. Epub 2015 Aug 17. PMID: 26279168;

PMCID: PMC4757509.

162: van de Vijver SJ, Oti SO, Agyemang C, Gomez GB, Kyobutungi C. Prevalence,

awareness, treatment and control of hypertension among slum dwellers in Nairobi,

Kenya. J Hypertens. 2013 May;31(5):1018-24. doi: 10.1097/HJH.0b013e32835e3a56.

PMID: 23425703.

163: Bastawrous A, Mathenge W, Wing K, Bastawrous M, Rono H, Weiss HA, Macleod

D, Foster A, Peto T, Blows P, Burton M, Kuper H. The incidence of diabetes

mellitus and diabetic retinopathy in a population-based cohort study of people

age 50 years and over in Nakuru, Kenya. BMC Endocr Disord. 2017 Mar 23;17(1):19.

doi: 10.1186/s12902-017-0170-x. PMID: 28335755; PMCID: PMC5364612.

164: Nuwaha F, Musinguzi G. Pre-hypertension in Uganda: a cross-sectional study.

BMC Cardiovasc Disord. 2013 Nov 14;13:101. doi: 10.1186/1471-2261-13-101. PMID:

24228945; PMCID: PMC3833647.

165: Unwin N, James P, McLarty D, Machybia H, Nkulila P, Tamin B, Nguluma M,

McNally R. Rural to urban migration and changes in cardiovascular risk factors

in Tanzania: a prospective cohort study. BMC Public Health. 2010 May 24;10:272.

doi: 10.1186/1471-2458-10-272. PMID: 20497567; PMCID: PMC2892446.

166: Whyte SR. Knowing hypertension and diabetes: Conditions of treatability in

Uganda. Health Place. 2016 May;39:219-25. doi:

10.1016/j.healthplace.2015.07.002. Epub 2015 Jul 29. PMID: 26233676.

167: Yonga G, Okello FO, Herr JL, Mulvaney A, Ogola EN. Healthy Heart Africa: a

prospective evaluation of programme outcomes on individuals' hypertension

awareness, screening, diagnosis and treatment in rural Kenya at 12 months.

Cardiovasc J Afr. 2020 Jan/Feb 23;31(1):9-15. doi: 10.5830/CVJA-2019-037. Epub

2019 Nov 27. PMID: 31781714.

168: Ploubidis GB, Mathenge W, De Stavola B, Grundy E, Foster A, Kuper H.

Socioeconomic position and later life prevalence of hypertension, diabetes and

visual impairment in Nakuru, Kenya. Int J Public Health. 2013 Feb;58(1):133-41.

doi: 10.1007/s00038-012-0389-2. Epub 2012 Jul 20. PMID: 22814479.

169: Oyando R, Njoroge M, Nguhiu P, Kirui F, Mbui J, Sigilai A, Bukania Z, Obala

A, Munge K, Etyang A, Barasa E. Patient costs of hypertension care in public

health care facilities in Kenya. Int J Health Plann Manage. 2019

Apr;34(2):e1166-e1178. doi: 10.1002/hpm.2752. Epub 2019 Feb 14. PMID: 30762904;

PMCID: PMC6618067.

170: Vedanthan R, Blank E, Tuikong N, Kamano J, Misoi L, Tulienge D, Hutchinson

C, Ascheim DD, Kimaiyo S, Fuster V, Were MC. Usability and feasibility of a

tablet-based Decision-Support and Integrated Record-keeping (DESIRE) tool in the

nurse management of hypertension in rural western Kenya. Int J Med Inform. 2015

Mar;84(3):207-19. doi: 10.1016/j.ijmedinf.2014.12.005. Epub 2015 Jan 7. PMID:

25612791; PMCID: PMC4314432.

171: Kamwesiga JT, Tham K, Guidetti S. Experiences of using mobile phones in

everyday life among persons with stroke and their families in Uganda - a

qualitative study. Disabil Rehabil. 2017 Mar;39(5):438-449. doi:

10.3109/09638288.2016.1146354. Epub 2016 Mar 3. PMID: 26939597.

172: Bloomfield GS, Hogan JW, Keter A, Sang E, Carter EJ, Velazquez EJ, Kimaiyo

S. Hypertension and obesity as cardiovascular risk factors among HIV

seropositive patients in Western Kenya. PLoS One. 2011;6(7):e22288. doi:

10.1371/journal.pone.0022288. Epub 2011 Jul 14. PMID: 21779407; PMCID:

PMC3136516.

173: Kimani S, Mirie W, Chege M, Okube OT, Muniu S. Association of lifestyle

modification and pharmacological adherence on blood pressure control among

patients with hypertension at Kenyatta National Hospital, Kenya: a cross-

sectional study. BMJ Open. 2019 Jan 17;9(1):e023995. doi:

10.1136/bmjopen-2018-023995. PMID: 30782721; PMCID: PMC6340423.

174: Werner ME, van de Vijver S, Adhiambo M, Egondi T, Oti SO, Kyobutungi C.

Results of a hypertension and diabetes treatment program in the slums of

Nairobi: a retrospective cohort study. BMC Health Serv Res. 2015 Nov 17;15:512.

doi: 10.1186/s12913-015-1167-7. PMID: 26577953; PMCID: PMC4650397.

175: Kotwani P, Kwarisiima D, Clark TD, Kabami J, Geng EH, Jain V, Chamie G,

Petersen ML, Thirumurthy H, Kamya MR, Charlebois ED, Havlir DV; SEARCH

Collaboration. Epidemiology and awareness of hypertension in a rural Ugandan

community: a cross-sectional study. BMC Public Health. 2013 Dec 9;13:1151. doi:

10.1186/1471-2458-13-1151. PMID: 24321133; PMCID: PMC3890617.

176: Nabbaale J, Kibirige D, Ssekasanvu E, Sebatta ES, Kayima J, Lwabi P,

Kalyesubula R. Microalbuminuria and left ventricular hypertrophy among newly

diagnosed black African hypertensive patients: a cross sectional study from a

tertiary hospital in Uganda. BMC Res Notes. 2015 May 14;8:198. doi:

10.1186/s13104-015-1156-2. PMID: 25971452; PMCID: PMC4434545.

177: Nakalema I, Kaddumukasa M, Nakibuuka J, Okello E, Sajatovic M, Katabira E.

Prevalence, patterns and factors associated with hypertensive crises in Mulago

hospital emergency department; a cross-sectional study. Afr Health Sci. 2019

Mar;19(1):1757-1767. doi: 10.4314/ahs.v19i1.52. PMID: 31149006; PMCID:

PMC6531930.

178: Abbas ZG, Lutale JK, Archibald LK. Diabetic foot ulcers and ethnicity in

Tanzania: a contrast between African and Asian populations. Int Wound J. 2009

Apr;6(2):124-31. doi: 10.1111/j.1742-481X.2008.00578.x. Epub 2009 Apr 2. PMID:

19368580.

179: Shaper AG, Jones KW. Serum-cholesterol, diet, and coronary heart-disease in

Africans and Asians in Uganda: 1959. Int J Epidemiol. 2012 Oct;41(5):1221-5.

doi: 10.1093/ije/dys137. PMID: 23045195.

180: Macheku GS, Philemon RN, Oneko O, Mlay PS, Masenga G, Obure J, Mahande MJ.

Frequency, risk factors and feto-maternal outcomes of abruptio placentae in

Northern Tanzania: a registry-based retrospective cohort study. BMC Pregnancy

Childbirth. 2015 Oct 7;15:242. doi: 10.1186/s12884-015-0678-x. PMID: 26446879;

PMCID: PMC4597387.

181: Akwanalo C, Njuguna B, Mercer T, Pastakia SD, Mwangi A, Dick J, Dickhaus J,

Andesia J, Bloomfield GS, Valente T, Kibachio J, Pillsbury M, Pathak S, Thakkar

A, Vedanthan R, Kamano J, Naanyu V. Strategies for Effective Stakeholder

Engagement in Strengthening Referral Networks for Management of Hypertension

Across Health Systems in Kenya. Glob Heart. 2019 Jun;14(2):173-179. doi:

10.1016/j.gheart.2019.06.003. PMID: 31324372; PMCID: PMC7250005.

182: Muddu M, Mutebi E, Mondo C. Prevalence, types and factors associated with

echocardiographic abnormalities among newly diagnosed diabetic patients at

Mulago Hospital. Afr Health Sci. 2016 Mar;16(1):183-93. doi:

10.4314/ahs.v16i1.25. PMID: 27358631; PMCID: PMC4915408.

183: Vedanthan R, Kamano JH, Lee H, Andama B, Bloomfield GS, DeLong AK, Edelman

D, Finkelstein EA, Hogan JW, Horowitz CR, Manyara S, Menya D, Naanyu V, Pastakia

SD, Valente TW, Wanyonyi CC, Fuster V. Bridging Income Generation with Group

Integrated Care for cardiovascular risk reduction: Rationale and design of the

BIGPIC study. Am Heart J. 2017 Jun;188:175-185. doi: 10.1016/j.ahj.2017.03.012.

Epub 2017 Mar 23. PMID: 28577673; PMCID: PMC5491075.

184: Manuthu EM, Joshi MD, Lule GN, Karari E. Prevalence of dyslipidemia and

dysglycaemia in HIV infected patients. East Afr Med J. 2008 Jan;85(1):10-7. doi:

10.4314/eamj.v85i1.9600. PMID: 18543521.

185: Musinguzi G, Nuwaha F. Prevalence, awareness and control of hypertension in

Uganda. PLoS One. 2013 Apr 17;8(4):e62236. doi: 10.1371/journal.pone.0062236.

PMID: 23614041; PMCID: PMC3629133.

186: Longenecker CT, Morris SR, Aliku TO, Beaton A, Costa MA, Kamya MR, Kityo C,

Lwabi P, Mirembe G, Nampijja D, Rwebembera J, Sable C, Salata RA, Scheel A,

Simon DI, Ssinabulya I, Okello E. Rheumatic Heart Disease Treatment Cascade in

Uganda. Circ Cardiovasc Qual Outcomes. 2017 Nov;10(11):e004037. doi:

10.1161/CIRCOUTCOMES.117.004037. PMID: 29133472; PMCID: PMC5728153.

187: Onyango MJ, Kombe I, Nyamongo DS, Mwangi M. A study to determine the

prevalence and factors associated with hypertension among employees working at a

call centre Nairobi Kenya. Pan Afr Med J. 2017 Jul 5;27:178. doi:

10.11604/pamj.2017.27.178.13073. PMID: 28904705; PMCID: PMC5579427.

188: Kabakambira JD, Niyonsenga Z, Hategeka M, Igiraneza G, Benurugo G, Lucero-

Prisno Iii DE, Hategeka C. Blood pressure measurement techniques: Assessing

performance in outpatient settings of a tertiary-level hospital in Rwanda. J

Clin Hypertens (Greenwich). 2018 Jun;20(6):1067-1072. doi: 10.1111/jch.13303.

Epub 2018 May 16. PMID: 29767423.

189: Kaddumukasa MN, Katabira E, Sajatovic M, Pundik S, Kaddumukasa M, Goldstein

LB. Influence of sodium consumption and associated knowledge on poststroke

hypertension in Uganda. Neurology. 2016 Sep 20;87(12):1198-205. doi:

10.1212/WNL.0000000000003117. Epub 2016 Aug 24. PMID: 27558374; PMCID:

PMC5035979.

190: Stanifer JW, Egger JR, Turner EL, Thielman N, Patel UD; Comprehensive

Kidney Disease Assessment for Risk factors, epidemiology, Knowledge, and

Attitudes (CKD AFRiKA) Study. Neighborhood clustering of non-communicable

diseases: results from a community-based study in Northern Tanzania. BMC Public

Health. 2016 Mar 5;16:226. doi: 10.1186/s12889-016-2912-5. PMID: 26944390;

PMCID: PMC4779220.

191: Rader F, Blyler CA. Finding Hay in the Haystack. J Am Coll Cardiol. 2019

Oct 15;74(15):1907-1909. doi: 10.1016/j.jacc.2019.08.1001. PMID: 31601370.

192: Babua C, Kalyesubula R, Okello E, Kakande B, Sebatta E, Mungoma M, Mondo

CK. Cardiovascular risk factors among patients with chronic kidney disease

attending a tertiary hospital in Uganda. Cardiovasc J Afr. 2015 Jul-

Aug;26(4):177-80. doi: 10.5830/CVJA-2015-045. PMID: 26407219; PMCID: PMC4683292.

193: Ayah R, Joshi MD, Wanjiru R, Njau EK, Otieno CF, Njeru EK, Mutai KK. A

population-based survey of prevalence of diabetes and correlates in an urban

slum community in Nairobi, Kenya. BMC Public Health. 2013 Apr 20;13:371. doi:

10.1186/1471-2458-13-371. PMID: 23601475; PMCID: PMC3641964.

194: Mworozi K, Ameda F, Byanyima RK, Nakasujja N. Carotid artery plaque

detected on ultrasound is associated with impaired cognitive state in the

elderly: A population-based study in Wakiso district, Uganda. J Clin Neurosci.

2019 Oct;68:194-200. doi: 10.1016/j.jocn.2019.06.011. Epub 2019 Jul 10. PMID:

31301929.

195: Galson SW, Staton CA, Karia F, Kilonzo K, Lunyera J, Patel UD, Hertz JT,

Stanifer JW. Epidemiology of hypertension in Northern Tanzania: a community-

based mixed-methods study. BMJ Open. 2017 Nov 9;7(11):e018829. doi:

10.1136/bmjopen-2017-018829. PMID: 29127232; PMCID: PMC5695455.

196: Kilonzo SB, Gunda DW, Bakshi FA, Kalokola F, Mayala HA, Dadi H. Control of

Hypertension among Diabetic Patients in a Referral Hospital in Tanzania: A

Cross-Sectional Study. Ethiop J Health Sci. 2017 Sep;27(5):473-480. doi:

10.4314/ejhs.v27i5.5. PMID: 29217952; PMCID: PMC5615008.

197: Haregu TN, Oti S, Ngomi N, Khayeka-Wandabwa C, Egondi T, Kyobutungi C.

Interlinkage among cardio-metabolic disease markers in an urban poor setting in

Nairobi, Kenya. Glob Health Action. 2016 Feb 9;9(0):30626. doi:

10.3402/gha.v9.30626. PMID: 26864740; PMCID: PMC4749862.

198: Wanzou JPV, Sekimpi P, Komagum JO, Nakwagala F, Mwaka ES. Charcot

arthropathy of the diabetic foot in a sub-Saharan tertiary hospital: a cross-

sectional study. J Foot Ankle Res. 2019 Jun 14;12:33. doi:

10.1186/s13047-019-0343-0. PMID: 31210786; PMCID: PMC6567465.

199: Mohamed AS, Sawe HR, Muhanuzi B, Marombwa NR, Mjema K, Weber EJ. Non-

traumatic chest pain in patients presenting to an urban emergency Department in

sub Saharan Africa: a prospective cohort study in Tanzania. BMC Cardiovasc

Disord. 2019 Jun 28;19(1):158. doi: 10.1186/s12872-019-1133-0. PMID: 31253098;

PMCID: PMC6599358.

200: Kisoli A, Gray WK, Dotchin CL, Orega G, Dewhurst F, Paddick SM, Longdon A,

Chaote P, Dewhurst M, Walker RW. Levels of functional disability in elderly

people in Tanzania with dementia, stroke and Parkinson's disease. Acta

Neuropsychiatr. 2015 Aug;27(4):206-12. doi: 10.1017/neu.2015.9. Epub 2015 Mar

17. PMID: 25777617.

201: Dzudie A, Ojji D, Anisiuba BC, Abdou BA, Cornick R, Damasceno A, Kane AL,

Mocumbi AO, Mohamed A, Nel G, Ogola E, Onwubere B, Otieno H, Rainer B, Schutte

A, Ali IT, Twagirumukiza M, Poulter N, Mayosi B; PASCAR Hypertension Task Force

members. Development of the roadmap and guidelines for the prevention and

management of high blood pressure in Africa: Proceedings of the PASCAR

Hypertension Task Force meeting: Nairobi, Kenya, 27 October 2014. Cardiovasc J

Afr. 2015 Mar-Apr;26(2):82-5. PMID: 25940121.

202: Okello E, Wanzhu Z, Musoke C, Twalib A, Kakande B, Lwabi P, Wilson NB,

Mondo CK, Odoi-Adome R, Freers J. Cardiovascular complications in newly

diagnosed rheumatic heart disease patients at Mulago Hospital, Uganda.

Cardiovasc J Afr. 2013 Apr;24(3):80-5. doi: 10.5830/CVJA-2013-004. PMID:

23736132; PMCID: PMC3721959.

203: van de Vijver S, Oti SO, Gomez GB, Agyemang C, Egondi T, Moll van Charante

E, Brewster LM, Hankins C, Tanovic Z, Ezeh A, Kyobutungi C, Stronks K. Impact

evaluation of a community-based intervention for prevention of cardiovascular

diseases in the slums of Nairobi: the SCALE-UP study. Glob Health Action. 2016

Mar 24;9:30922. doi: 10.3402/gha.v9.30922. PMID: 27019347; PMCID: PMC4808680.

204: Okello E, Kakande B, Sebatta E, Kayima J, Kuteesa M, Mutatina B, Nyakoojo

W, Lwabi P, Mondo CK, Odoi-Adome R, Juergen F. Socioeconomic and environmental

risk factors among rheumatic heart disease patients in Uganda. PLoS One.

2012;7(8):e43917. doi: 10.1371/journal.pone.0043917. Epub 2012 Aug 27. PMID:

22952810; PMCID: PMC3428272.

205: Mbalilaki JA, Masesa Z, Strømme SB, Høstmark AT, Sundquist J, Wändell P,

Rosengren A, Hellenius ML. Daily energy expenditure and cardiovascular risk in

Masai, rural and urban Bantu Tanzanians. Br J Sports Med. 2010 Feb;44(2):121-6.

doi: 10.1136/bjsm.2007.044966. Epub 2008 Jun 3. PMID: 18523037.

206: Vululi ST, Bugeza S, Zeridah M, Ddungu H, Openy AB, Frank M, Parkes-

Ratanshi R. Prevalence of lower limb deep venous thrombosis among adult HIV

positive patients attending an outpatient clinic at Mulago Hospital. AIDS Res

Ther. 2018 Jan 25;15(1):3. doi: 10.1186/s12981-018-0191-1. PMID: 29370816;

PMCID: PMC5784710.

207: Etyang AO, Kapesa S, Odipo E, Bauni E, Kyobutungi C, Abdalla M, Muntner P,

Musani SK, Macharia A, Williams TN, Cruickshank JK, Smeeth L, Scott JAG. Effect

of Previous Exposure to Malaria on Blood Pressure in Kilifi, Kenya: A Mendelian

Randomization Study. J Am Heart Assoc. 2019 Mar 19;8(6):e011771. doi:

10.1161/JAHA.118.011771. PMID: 30879408; PMCID: PMC6475058.

208: Mercer T, Njuguna B, Bloomfield GS, Dick J, Finkelstein E, Kamano J, Mwangi

A, Naanyu V, Pastakia SD, Valente TW, Vedanthan R, Akwanalo C. Strengthening

Referral Networks for Management of Hypertension Across the Health System

(STRENGTHS) in western Kenya: a study protocol of a cluster randomized trial.

Trials. 2019 Sep 9;20(1):554. doi: 10.1186/s13063-019-3661-4. PMID: 31500661;

PMCID: PMC6734355.

209: Mwita CC, Akello W, Sisenda G, Ogoti E, Tivey D, Munn Z, Mbogo D.

Assessment of cardiovascular risk and target organ damage among adult patients

with primary hypertension in Thika Level 5 Hospital, Kenya: a criteria-based

clinical audit. Int J Evid Based Healthc. 2013 Jun;11(2):115-20. doi:

10.1111/1744-1609.12014. PMID: 23750574.

210: Peck RN, Green E, Mtabaji J, Majinge C, Smart LR, Downs JA, Fitzgerald DW.

Hypertension-related diseases as a common cause of hospital mortality in

Tanzania: a 3-year prospective study. J Hypertens. 2013 Sep;31(9):1806-11. doi:

10.1097/HJH.0b013e328362bad7. PMID: 23777761; PMCID: PMC4005815.

211: Putnam HWI, Jones R, Rogathi J, Gray WK, Swai B, Dewhurst M, Dewhurst F,

Walker RW. Hypertension in a resource-limited setting: Is it associated with end

organ damage in older adults in rural Tanzania? J Clin Hypertens (Greenwich).

2018 Feb;20(2):217-224. doi: 10.1111/jch.13187. Epub 2018 Feb 15. PMID:

29446219.

212: Okello S, Kanyesigye M, Muyindike WR, Annex BH, Hunt PW, Haneuse S, Siedner

MJ. Incidence and predictors of hypertension in adults with HIV-initiating

antiretroviral therapy in south-western Uganda. J Hypertens. 2015

Oct;33(10):2039-45. doi: 10.1097/HJH.0000000000000657. PMID: 26431192; PMCID:

PMC4700387.

213: Magan T, Pouncey A, Gadhvi K, Katta M, Posner M, Davey C. Prevalence and

severity of diabetic retinopathy in patients attending the endocrinology

diabetes clinic at Mulago Hospital in Uganda. Diabetes Res Clin Pract. 2019

Jun;152:65-70. doi: 10.1016/j.diabres.2019.04.024. Epub 2019 May 4. PMID:

31063850.

214: Muhanuzi B, Sawe HR, Kilindimo SS, Mfinanga JA, Weber EJ. Respiratory

compromise in children presenting to an urban emergency department of a tertiary

hospital in Tanzania: a descriptive cohort study. BMC Emerg Med. 2019 Feb

28;19(1):21. doi: 10.1186/s12873-019-0235-4. PMID: 30819093; PMCID: PMC6393970.

215: Hulzebosch A, van de Vijver S, Oti SO, Egondi T, Kyobutungi C. Profile of

people with hypertension in Nairobi's slums: a descriptive study. Global Health.

2015 Jun 27;11:26. doi: 10.1186/s12992-015-0112-1. PMID: 26116577; PMCID:

PMC4491223.

216: Vedanthan R, Tuikong N, Kofler C, Blank E, Kamano JH, Naanyu V, Kimaiyo S,

Inui TS, Horowitz CR, Fuster V. Barriers and Facilitators to Nurse Management of

Hypertension: A Qualitative Analysis from Western Kenya. Ethn Dis. 2016 Jul

21;26(3):315-22. doi: 10.18865/ed.26.3.315. PMID: 27440970; PMCID: PMC4948797.

217: Scheel A, Ssinabulya I, Aliku T, Bradley-Hewitt T, Clauss A, Clauss S,

Crawford L, DeWyer A, Donofrio MT, Jacobs M, Klein J, Moore TE, Okello E, Scheel

J, Shaw R, Sable C, Lwabi P, Watkins DA, Beaton A. Community study to uncover

the full spectrum of rheumatic heart disease in Uganda. Heart. 2019

Jan;105(1):60-66. doi: 10.1136/heartjnl-2018-313171. Epub 2018 Aug 1. PMID:

30068532.

218: Kakarmath SS, Zack RM, Leyna GH, Fahimi S, Liu E, Fawzi WW, Lukmanji Z,

Killewo J, Sacks F, Danaei G. Dietary determinants of serum total cholesterol

among middle-aged and older adults: a population-based cross-sectional study in

Dar es Salaam, Tanzania. BMJ Open. 2017 Jun 6;7(5):e015028. doi:

10.1136/bmjopen-2016-015028. PMID: 28588111; PMCID: PMC5663012.

219: Ogeng'o JA, Olabu BO, Ong'era D, Sinkeet SR. Pattern of acute myocardial

infarction in an African country. Acta Cardiol. 2010 Dec;65(6):613-8. doi:

10.2143/AC.65.6.2059856. PMID: 21302665.

220: Jablonski-Cohen MS, Kosgei RJ, Rerimoi AJ, Mamlin JJ. The emerging problem

of coronary heart disease in Kenya. East Afr Med J. 2003 Jun;80(6):293-7. doi:

10.4314/eamj.v80i6.8704. PMID: 12953737.

221: Gray WK, Dewhurst F, Dewhurst MJ, Orega G, Kissima J, Chaote P, Walker RW.

Rates and predictors of three-year mortality in older people in rural Tanzania.

Arch Gerontol Geriatr. 2016 Jan-Feb;62:36-42. doi:

10.1016/j.archger.2015.10.008. Epub 2015 Oct 31. PMID: 26549489.

222: Meme N, Amwayi S, Nganga Z, Buregyeya E. Prevalence of undiagnosed diabetes

and pre-diabetes among hypertensive patients attending Kiambu district Hospital,

Kenya: a cross-sectional study. Pan Afr Med J. 2015 Nov 24;22:286. doi:

10.11604/pamj.2015.22.286.7395. PMID: 26966482; PMCID: PMC4769056.

223: Muturi A, Ndaguatha P, Ojuka D, Kibet A. Prevalence and predictors of

intra-abdominal hypertension and compartment syndrome in surgical patients in

critical care units at Kenyatta National Hospital. BMC Emerg Med. 2017 Mar

23;17(1):10. doi: 10.1186/s12873-017-0120-y. PMID: 28330440; PMCID: PMC5363018.

224: Edwards R, Unwin N, Mugusi F, Whiting D, Rashid S, Kissima J, Aspray TJ,

Alberti KG. Hypertension prevalence and care in an urban and rural area of

Tanzania. J Hypertens. 2000 Feb;18(2):145-52. doi:

10.1097/00004872-200018020-00003. PMID: 10694181.

225: Nyaruhucha CN, Msuya JM, Augustino B. Health status of hospitalised elderly

in Morogoro Regional Hospital. East Afr Med J. 2001 Sep;78(9):489-92. doi:

10.4314/eamj.v78i9.8982. PMID: 11921585.

226: Nankabirwa H, Kalyesubula R, Ssinabulya I, Katabira ET, Cumming RG. A

cross-sectional study of hyponatraemia among elderly patients with heart failure

in Uganda. BMJ Open. 2016 May 17;6(5):e009775. doi: 10.1136/bmjopen-2015-009775.

PMID: 27188802; PMCID: PMC4874129.

227: Lule SA, Namara B, Akurut H, Muhangi L, Lubyayi L, Nampijja M, Akello F,

Tumusiime J, Aujo JC, Oduru G, Smeeth L, Elliott AM, Webb EL. Are birthweight

and postnatal weight gain in childhood associated with blood pressure in early

adolescence? Results from a Ugandan birth cohort. Int J Epidemiol. 2019 Feb

1;48(1):148-156. doi: 10.1093/ije/dyy118. PMID: 29982658; PMCID: PMC6380421.

228: Mayega RW, Makumbi F, Rutebemberwa E, Peterson S, Östenson CG, Tomson G,

Guwatudde D. Modifiable socio-behavioural factors associated with overweight and

hypertension among persons aged 35 to 60 years in eastern Uganda. PLoS One.

2012;7(10):e47632. doi: 10.1371/journal.pone.0047632. Epub 2012 Oct 15. PMID:

23077653; PMCID: PMC3471867.

229: Lasky D, Becerra E, Boto W, Otim M, Ntambi J. Obesity and gender

differences in the risk of type 2 diabetes mellitus in Uganda. Nutrition. 2002

May;18(5):417-21. doi: 10.1016/s0899-9007(01)00726-2. PMID: 11985948.

230: Walker RW, Jusabani A, Aris E, Gray WK, Mitra D, Swai M. A prospective

study of stroke sub-type from within an incident population in Tanzania. S Afr

Med J. 2011 May;101(5):338-44. doi: 10.7196/samj.4511. PMID: 21837879.

231: Kibudde S, Mondo CK, Kibirige D, Walusansa V, Orem J. Anthracycline induced

cardiotoxicity in adult cancer patients: a prospective cohort study from a

specialized oncology treatment centre in Uganda. Afr Health Sci. 2019

Mar;19(1):1647-1656. doi: 10.4314/ahs.v19i1.40. PMID: 31148994; PMCID:

PMC6531975.

232: Siedner MJ, Kim JH, Nakku RS, Bibangambah P, Hemphill L, Triant VA, Haberer

JE, Martin JN, Mocello AR, Boum Y 2nd, Kwon DS, Tracy RP, Burdo T, Huang Y, Cao

H, Okello S, Bangsberg DR, Hunt PW. Persistent Immune Activation and Carotid

Atherosclerosis in HIV-Infected Ugandans Receiving Antiretroviral Therapy. J

Infect Dis. 2016 Feb 1;213(3):370-8. doi: 10.1093/infdis/jiv450. Epub 2015 Sep

7. PMID: 26347573; PMCID: PMC4704672.

233: Mateen FJ, Kanters S, Kalyesubula R, Mukasa B, Kawuma E, Kengne AP, Mills

EJ. Hypertension prevalence and Framingham risk score stratification in a large

HIV-positive cohort in Uganda. J Hypertens. 2013 Jul;31(7):1372-8; discussion

1378. doi: 10.1097/HJH.0b013e328360de1c. PMID: 23615323.

234: Cleland CR, Burton MJ, Hall C, Hall A, Courtright P, Makupa WU, Philippin

H. Diabetic retinopathy in Tanzania: prevalence and risk factors at entry into a

regional screening programme. Trop Med Int Health. 2016 Mar;21(3):417-26. doi:

10.1111/tmi.12652. Epub 2016 Jan 13. PMID: 26644361; PMCID: PMC4819693.

235: Maaløe N, Andersen CB, Housseine N, Meguid T, Bygbjerg IC, van Roosmalen J.

Effect of locally tailored clinical guidelines on intrapartum management of

severe hypertensive disorders at Zanzibar's tertiary hospital (the PartoMa

study). Int J Gynaecol Obstet. 2019 Jan;144(1):27-36. doi: 10.1002/ijgo.12692.

Epub 2018 Oct 26. PMID: 30307609; PMCID: PMC7379925.

236: van de Vijver S, Oti S, Tervaert TC, Hankins C, Kyobutungi C, Gomez GB,

Brewster L, Agyemang C, Lange J. Introducing a model of cardiovascular

prevention in Nairobi's slums by integrating a public health and private-sector

approach: the SCALE-UP study. Glob Health Action. 2013 Oct 21;6:22510. doi:

10.3402/gha.v6i0.22510. PMID: 24149078; PMCID: PMC3805842.

237: Okello S, Nasasira B, Muiru AN, Muyingo A. Validity and Reliability of a

Self-Reported Measure of Antihypertensive Medication Adherence in Uganda. PLoS

One. 2016 Jul 1;11(7):e0158499. doi: 10.1371/journal.pone.0158499. Erratum in:

PLoS One. 2017 Oct 31;12 (10 ):e0187620. PMID: 27367542; PMCID: PMC4930194.

238: Saidi H, Smart LR, Kamugisha E, Ambrose EE, Soka D, Peck RN, Makani J.

Complications of sickle cell anaemia in children in Northwestern Tanzania.

Hematology. 2016 May;21(4):248-256. doi: 10.1080/10245332.2015.1101976. Epub

2016 Feb 17. PMID: 26868490; PMCID: PMC4972452.

239: Eberly LA, Rusingiza E, Park PH, Ngoga G, Dusabeyezu S, Mutabazi F,

Harerimana E, Mucumbitsi J, Nyembo PF, Borg R, Gahamanyi C, Mutumbira C,

Ntaganda E, Rusangwa C, Kwan GF, Bukhman G. Nurse-Driven Echocardiography and

Management of Heart Failure at District Hospitals in Rural Rwanda. Circ

Cardiovasc Qual Outcomes. 2018 Dec;11(12):e004881. doi:

10.1161/CIRCOUTCOMES.118.004881. PMID: 30562070.

240: Kaddumukasa M, Nakibuuka J, Mugenyi L, Namusoke O, Birungi D, Kabaala B,

Blixen C, Katabira E, Furlan A, Sajatovic M. Feasibility study of a targeted

self-management intervention for reducing stroke risk factors in a high-risk

population in Uganda. J Neurol Sci. 2018 Mar 15;386:23-28. doi:

10.1016/j.jns.2017.12.032. Epub 2018 Jan 4. PMID: 29406961; PMCID: PMC5804990.

241: Ploutz M, Aliku T, Bradley-Hewitt T, Dantin A, Lemley B, Gillespie CW,

Lwabi P, Sable C, Beaton A. Child and teacher acceptability of school-based

echocardiographic screening for rheumatic heart disease in Uganda. Cardiol

Young. 2017 Jan;27(1):82-89. doi: 10.1017/S1047951116000159. Epub 2016 Mar 17.

PMID: 26983378.

242: Batte A, Lwabi P, Lubega S, Kiguli S, Nabatte V, Karamagi C. Prevalence of

arrhythmias among children below 15 years of age with congenital heart diseases

attending Mulago National Referral Hospital, Uganda. BMC Cardiovasc Disord. 2016

Apr 13;16:67. doi: 10.1186/s12872-016-0243-1. PMID: 27074797; PMCID: PMC4831118.

243: Kingery JR, Yango M, Wajanga B, Kalokola F, Brejt J, Kataraihya J, Peck R.

Heart failure, post-hospital mortality and renal function in Tanzania: A

prospective cohort study. Int J Cardiol. 2017 Sep 15;243:311-317. doi:

10.1016/j.ijcard.2017.05.025. Epub 2017 May 9. PMID: 28522164; PMCID:

PMC5570474.

244: Makubi A, Hage C, Lwakatare J, Kisenge P, Makani J, Rydén L, Lund LH.

Contemporary aetiology, clinical characteristics and prognosis of adults with

heart failure observed in a tertiary hospital in Tanzania: the prospective

Tanzania Heart Failure (TaHeF) study. Heart. 2014 Aug;100(16):1235-41. doi:

10.1136/heartjnl-2014-305599. Epub 2014 Apr 17. PMID: 24743164; PMCID:

PMC5555386.

245: Ndejjo R, Musinguzi G, Nuwaha F, Wanyenze RK, Bastiaens H. Acceptability of

a community cardiovascular disease prevention programme in Mukono and Buikwe

districts in Uganda: a qualitative study. BMC Public Health. 2020 Jan

16;20(1):75. doi: 10.1186/s12889-020-8188-9. PMID: 31948423; PMCID: PMC6966788.

246: Ogeng'o JA, Otieno B. Aneurysms in the arteries of the upper extremity in a

Kenyan population. Cardiovasc Pathol. 2011 Jan-Feb;20(1):e53-6. doi:

10.1016/j.carpath.2010.01.009. Epub 2010 Feb 2. PMID: 20129800.

247: Tusubira AK, Akiteng AR, Nakirya BD, Nalwoga R, Ssinabulya I, Nalwadda CK,

Schwartz JI. Accessing medicines for non-communicable diseases: Patients and

health care workers' experiences at public and private health facilities in

Uganda. PLoS One. 2020 Jul 7;15(7):e0235696. doi: 10.1371/journal.pone.0235696.

PMID: 32634164; PMCID: PMC7340292.

248: Bizimana JB, Lawani MM, Akplogan B, Gaturagi C. Activités physiques libres

ou encadrées et condition physique liée à la santé chez des adultes burundais:

étude transversale [Nonorganized and organized physical activity and health-

related physical fitness in Burundian adults: cross-sectional study]. Pan Afr

Med J. 2016 Sep 28;25:38. French. doi: 10.11604/pamj.2016.25.38.7688. PMID:

28203315; PMCID: PMC5292119.

249: Paddick SM, Kisoli A, Samuel M, Higginson J, Gray WK, Dotchin CL, Longdon

AR, Teodorczuk A, Chaote P, Walker RW. Mild Cognitive Impairment in Rural

Tanzania: Prevalence, Profile, and Outcomes at 4-Year Follow-up. Am J Geriatr

Psychiatry. 2015 Sep;23(9):950-9. doi: 10.1016/j.jagp.2014.12.005. Epub 2014 Dec

11. PMID: 25579049.

250: Settumba SN, Sweeney S, Seeley J, Biraro S, Mutungi G, Munderi P,

Grosskurth H, Vassall A. The health system burden of chronic disease care: an

estimation of provider costs of selected chronic diseases in Uganda. Trop Med

Int Health. 2015 Jun;20(6):781-90. doi: 10.1111/tmi.12487. Epub 2015 Mar 6.

PMID: 25707376; PMCID: PMC4973817.

251: Maar M, Yeates K, Barron M, Hua D, Liu P, Moy Lum-Kwong M, Perkins N,

Sleeth J, Tobe J, Wabano MJ, Williamson P, Tobe SW. I-RREACH: an engagement and

assessment tool for improving implementation readiness of researchers,

organizations and communities in complex interventions. Implement Sci. 2015 May

4;10:64. doi: 10.1186/s13012-015-0257-6. PMID: 25935849; PMCID: PMC4424962.

252: Kayima J, Nankabirwa J, Sinabulya I, Nakibuuka J, Zhu X, Rahman M,

Longenecker CT, Katamba A, Mayanja-Kizza H, Kamya MR. Determinants of

hypertension in a young adult Ugandan population in epidemiological transition-

the MEPI-CVD survey. BMC Public Health. 2015 Aug 28;15:830. doi:

10.1186/s12889-015-2146-y. PMID: 26315787; PMCID: PMC4552375.

253: Kaddumukasa M, Ddumba E, Duncan P, Goldstein LB. Poststroke hypertension in

Africa. Stroke. 2012 Dec;43(12):3402-4. doi: 10.1161/STROKEAHA.112.672485. Epub

2012 Sep 25. PMID: 23010675; PMCID: PMC3508341.

254: Masyuko SJ, Page ST, Kinuthia J, Osoti AO, Polyak SJ, Otieno FC, Kibachio

JM, Mogaka JN, Temu TM, Zifodya JS, Otedo A, Nakanjako D, Hughes JP, Farquhar C.

Metabolic syndrome and 10-year cardiovascular risk among HIV-positive and HIV-

negative adults: A cross-sectional study. Medicine (Baltimore). 2020 Jul

2;99(27):e20845. doi: 10.1097/MD.0000000000020845. PMID: 32629671; PMCID:

PMC7337552.

255: Biraguma J, Mutimura E, Frantz JM. Health-related quality of life and

associated factors in adults living with HIV in Rwanda. SAHARA J. 2018

Dec;15(1):110-120. doi: 10.1080/17290376.2018.1520144. PMID: 30200815; PMCID:

PMC6136357.

256: Hovis IW, Namuyonga J, Kisitu GP, Ndagire E, Okello E, Longenecker CT,

Sanyahumbi A, Sable CA, Penny DJ, Lwabi P, Kekitiinwa AR, Beaton A. Decreased

Prevalence of Rheumatic Heart Disease Confirmed Among HIV-positive Youth.

Pediatr Infect Dis J. 2019 Apr;38(4):406-409. doi: 10.1097/INF.0000000000002161.

PMID: 30882733; PMCID: PMC6355385.

257: Bakari M, Chillo P, Lwakatare J. Factors associated with, and

echocardiographic findings of heart failure among HIV infected patients at a

tertiary health care facility in Dar es Salaam, Tanzania. Tanzan J Health Res.

2013 Apr;15(2):73-81. doi: 10.4314/thrb.v15i2.1. PMID: 26591712.

258: Debussche X, Balcou-Debussche M, Baranderaka NA, Ndayirorere S, La Hausse

de Lalouviére V, Nitunga N. Réseau d'éducation en santé communautaire pour la

prévention des maladies cardiovasculaires et du diabète au Burundi: mise en

place et premiers résultats [Community health education network for the

prevention of cardiovascular disease and diabetes in Burundi: development and

first results]. Glob Health Promot. 2010 Jun;17(2 Suppl):68-75. French. doi:

10.1177/1757975910366515. PMID: 20595345.

259: Naemi R, Chockalingam N, Lutale JK, Abbas ZG. Can a combination of

lifestyle and clinical characteristics explain the presence of foot ulcer in

patients with diabetes? J Diabetes Complications. 2019 Jun;33(6):437-444. doi:

10.1016/j.jdiacomp.2019.02.006. Epub 2019 Mar 1. PMID: 31005476.

260: Maher D, Waswa L, Baisley K, Karabarinde A, Unwin N. Epidemiology of

hypertension in low-income countries: a cross-sectional population-based survey

in rural Uganda. J Hypertens. 2011 Jun;29(6):1061-8. doi:

10.1097/HJH.0b013e3283466e90. PMID: 21505357.

261: Pastakia SD, Manyara SM, Vedanthan R, Kamano JH, Menya D, Andama B, Chesoli

C, Laktabai J. Impact of Bridging Income Generation with Group Integrated Care

(BIGPIC) on Hypertension and Diabetes in Rural Western Kenya. J Gen Intern Med.

2017 May;32(5):540-548. doi: 10.1007/s11606-016-3918-5. Epub 2016 Dec 5. PMID:

27921256; PMCID: PMC5400758.

262: Scheel A, Beaton A, Okello E, Longenecker CT, Otim IO, Lwabi P, Sable C,

Webel AR, Aliku T. The impact of a peer support group for children with

rheumatic heart disease in Uganda. Patient Educ Couns. 2018 Jan;101(1):119-123.

doi: 10.1016/j.pec.2017.07.006. Epub 2017 Jul 11. PMID: 28716486.

263: Kazooba P, Kasamba I, Mayanja BN, Lutaakome J, Namakoola I, Salome T,

Kaleebu P, Munderi P. Cardiometabolic risk among HIV-POSITIVE Ugandan adults:

prevalence, predictors and effect of long-term antiretroviral therapy. Pan Afr

Med J. 2017 May 15;27:40. doi: 10.11604/pamj.2017.27.40.9840. PMID: 28761616;

PMCID: PMC5516660.

264: Vedanthan R, Kamano JH, Naanyu V, Delong AK, Were MC, Finkelstein EA, Menya

D, Akwanalo CO, Bloomfield GS, Binanay CA, Velazquez EJ, Hogan JW, Horowitz CR,

Inui TS, Kimaiyo S, Fuster V. Optimizing linkage and retention to hypertension

care in rural Kenya (LARK hypertension study): study protocol for a randomized

controlled trial. Trials. 2014 Apr 27;15:143. doi: 10.1186/1745-6215-15-143.

PMID: 24767476; PMCID: PMC4113229.

265: Kurji K, Kiage D, Rudnisky CJ, Damji KF. Improving diabetic retinopathy

screening in Africa: patient satisfaction with teleophthalmology versus

ophthalmologist-based screening. Middle East Afr J Ophthalmol. 2013 Jan-

Mar;20(1):56-60. doi: 10.4103/0974-9233.106388. PMID: 23580853; PMCID:

PMC3617530.

266: Oti SO, van de Vijver SJ, Kyobutungi C, Gomez GB, Agyemang C, Moll van

Charante EP, Brewster LM, Hendriks ME, Schultsz C, Ettarh R, Ezeh A, Lange J. A

community-based intervention for primary prevention of cardiovascular diseases

in the slums of Nairobi: the SCALE UP study protocol for a prospective quasi-

experimental community-based trial. Trials. 2013 Dec 1;14:409. doi:

10.1186/1745-6215-14-409. PMID: 24289751; PMCID: PMC4220814.

267: Ogeng'o JA, Gatonga P, Olabu BO, Ongera D. Pattern of hypertensive kidney

disease in a black Kenyan population. Cardiology. 2011;120(3):125-9. doi:

10.1159/000334433. Epub 2011 Dec 15. PMID: 22179118.

268: Naanyu V, Vedanthan R, Kamano JH, Rotich JK, Lagat KK, Kiptoo P, Kofler C,

Mutai KK, Bloomfield GS, Menya D, Kimaiyo S, Fuster V, Horowitz CR, Inui TS.

Barriers Influencing Linkage to Hypertension Care in Kenya: Qualitative Analysis

from the LARK Hypertension Study. J Gen Intern Med. 2016 Mar;31(3):304-14. doi:

10.1007/s11606-015-3566-1. Epub 2016 Jan 4. PMID: 26728782; PMCID: PMC4762819.

269: Bovet P, Ross AG, Gervasoni JP, Mkamba M, Mtasiwa DM, Lengeler C, Whiting

D, Paccaud F. Distribution of blood pressure, body mass index and smoking habits

in the urban population of Dar es Salaam, Tanzania, and associations with

socioeconomic status. Int J Epidemiol. 2002 Feb;31(1):240-7. doi:

10.1093/ije/31.1.240. PMID: 11914327.

270: Oti SO, van de Vijver S, Gomez GB, Agyemang C, Egondi T, Kyobutungi C,

Stronks K. Outcomes and costs of implementing a community-based intervention for

hypertension in an urban slum in Kenya. Bull World Health Organ. 2016 Jul

1;94(7):501-9. doi: 10.2471/BLT.15.156513. Epub 2016 Apr 26. PMID: 27429489;

PMCID: PMC4933135.

271: Irazola VE, Gutierrez L, Bloomfield G, Carrillo-Larco RM, Dorairaj P,

Gaziano T, Levitt NS, Miranda JJ, Ortiz AB, Steyn K, Wu Y, Xavier D, Yan LL, He

J, Rubinstein A. Hypertension Prevalence, Awareness, Treatment, and Control in

Selected LMIC Communities: Results From the NHLBI/UHG Network of Centers of

Excellence for Chronic Diseases. Glob Heart. 2016 Mar;11(1):47-59. doi:

10.1016/j.gheart.2015.12.008. PMID: 27102022; PMCID: PMC4843831.

272: Walker R, Whiting D, Unwin N, Mugusi F, Swai M, Aris E, Jusabani A, Kabadi

G, Gray WK, Lewanga M, Alberti G. Stroke incidence in rural and urban Tanzania:

a prospective, community-based study. Lancet Neurol. 2010 Aug;9(8):786-92. doi:

10.1016/S1474-4422(10)70144-7. Epub 2010 Jul 6. PMID: 20609629.

273: Coors ME, Matthew TL, Matthew DB. Ethical precepts for medical

volunteerism: including local voices and values to guide RHD surgery in Rwanda.

J Med Ethics. 2015 Oct;41(10):814-9. doi: 10.1136/medethics-2013-101694. Epub

2015 Jun 11. PMID: 26066361.

274: Ondoa-Onama C, Tumwine JK. Immediate outcome of babies with low Apgar score

in Mulago Hospital, Uganda. East Afr Med J. 2003 Jan;80(1):22-9. doi:

10.4314/eamj.v80i1.8662. PMID: 12755238.

275: Rugwizangoga B, Mwabili T, Scanlan T, Meyer P, Kitinya J. Coats' disease in

Tanzania: first case report and literature review. Afr Health Sci. 2014

Sep;14(3):763-8. doi: 10.4314/ahs.v14i3.37. PMID: 25352900; PMCID: PMC4209640.

276: Okeng'o K, Chillo P, Gray WK, Walker RW, Matuja W. Early Mortality and

Associated Factors among Patients with Stroke Admitted to a Large Teaching

Hospital in Tanzania. J Stroke Cerebrovasc Dis. 2017 Apr;26(4):871-878. doi:

10.1016/j.jstrokecerebrovasdis.2016.10.037. Epub 2016 Nov 29. PMID: 27913201.

277: Rasmussen JB, Mwaniki DL, Kaduka LU, Boit MK, Borch-Johnsen K, Friis H,

Christensen DL. Hemoglobin levels and blood pressure are associated in rural

black africans. Am J Hum Biol. 2016 Jan-Feb;28(1):145-8. doi:

10.1002/ajhb.22758. Epub 2015 Jun 19. PMID: 26087952.

278: Nakimuli A, Nakubulwa S, Kakaire O, Osinde MO, Mbalinda SN, Kakande N,

Nabirye RC, Kaye DK. The burden of maternal morbidity and mortality attributable

to hypertensive disorders in pregnancy: a prospective cohort study from Uganda.

BMC Pregnancy Childbirth. 2016 Aug 4;16:205. doi: 10.1186/s12884-016-1001-1.

PMID: 27492552; PMCID: PMC4973370.

279: Bloomfield GS, Temu TM, Akwanalo CO, Chen PS, Emonyi W, Heckbert SR, Koech

MM, Manji I, Shen C, Vatta M, Velazquez EJ, Wessel J, Kimaiyo S, Inui TS.

Genetic mutations in African patients with atrial fibrillation: Rationale and

design of the Study of Genetics of Atrial Fibrillation in an African Population

(SIGNAL). Am Heart J. 2015 Sep;170(3):455-64.e5. doi: 10.1016/j.ahj.2015.06.008.

Epub 2015 Jun 14. PMID: 26385028; PMCID: PMC4575772.

280: Odar E, Wandabwa J, Kiondo P. Maternal and fetal outcome of gestational

diabetes mellitus in Mulago Hospital, Uganda. Afr Health Sci. 2004

Apr;4(1):9-14. PMID: 15126187; PMCID: PMC2141655.

281: Simiyu IN, Mchaile DN, Katsongeri K, Philemon RN, Msuya SE. Prevalence,

severity and early outcomes of hypoxic ischemic encephalopathy among newborns at

a tertiary hospital, in northern Tanzania. BMC Pediatr. 2017 May 25;17(1):131.

doi: 10.1186/s12887-017-0876-y. PMID: 28545428; PMCID: PMC5445334.

282: Mshana G, Hampshire K, Panter-Brick C, Walker R. Urban-rural contrasts in

explanatory models and treatment-seeking behaviours for stroke in Tanzania. J

Biosoc Sci. 2008 Jan;40(1):35-52. doi: 10.1017/S0021932007002295. Epub 2007 Sep

4. PMID: 17767790.

283: Viswanathan V, Wadud JR, Madhavan S, Rajasekar S, Kumpatla S, Lutale JK,

Abbas ZG. Comparison of post amputation outcome in patients with type 2 diabetes

from specialized foot care centres in three developing countries. Diabetes Res

Clin Pract. 2010 May;88(2):146-50. doi: 10.1016/j.diabres.2010.02.015. Epub 2010

Mar 17. PMID: 20299119.

284: Krebs E, Gerardo CJ, Park LP, Nickenig Vissoci JR, Byiringiro JC,

Byiringiro F, Rulisa S, Thielman NM, Staton CA. Mortality-Associated

Characteristics of Patients with Traumatic Brain Injury at the University

Teaching Hospital of Kigali, Rwanda. World Neurosurg. 2017 Jun;102:571-582. doi:

10.1016/j.wneu.2017.03.001. Epub 2017 Mar 21. Erratum in: World Neurosurg. 2017

Oct;106:1047. PMID: 28336445; PMCID: PMC5681277.

285: Mbuya FE, Fredrick F, Kundi B. Knowledge of diabetes and hypertension among

members of teaching staff of higher learning institutions in Dar es Salaam,

Tanzania. Tanzan J Health Res. 2014 Apr;16(2):98-103. doi: 10.4314/thrb.v16i2.5.

PMID: 26875303.

286: Olotu A, Ndiritu M, Ismael M, Mohammed S, Mithwani S, Maitland K, Newton

CR. Characteristics and outcome of cardiopulmonary resuscitation in hospitalised

African children. Resuscitation. 2009 Jan;80(1):69-72. doi:

10.1016/j.resuscitation.2008.09.019. Epub 2008 Nov 14. PMID: 19013705; PMCID:

PMC2706393.

287: Sang HK. Agrammatic aphasia verb and argument patterns in Kiswahili-English

spontaneous language. S Afr J Commun Disord. 2015 Jun 8;62(1):E1-10. doi:

10.4102/sajcd.v62i1.89. PMID: 26304215.

288: Kentoffio K, Albano A, Koplan B, Feng M, Muthalaly RG, Campbell JI,

Sentongo R, Tracy RP, Peck R, Okello S, Tsai AC, Siedner MJ.

Electrocardiographic Evidence of Cardiac Disease by Sex and HIV Serostatus in

Mbarara, Uganda. Glob Heart. 2019 Dec;14(4):395-397. doi:

10.1016/j.gheart.2019.08.007. Epub 2019 Oct 1. PMID: 31585846; PMCID:

PMC7017713.

289: Miller N, Gray WK, Howitt SC, Jusabani A, Swai M, Mugusi F, Jones MP,

Walker RW. Aphasia and swallowing problems in subjects with incident stroke in

rural northern Tanzania: a case-control study. Top Stroke Rehabil. 2014 Jan-

Feb;21(1):52-62. doi: 10.1310/tsr2101-52. PMID: 24521840.

290: Guwatudde D, Mutungi G, Wesonga R, Kajjura R, Kasule H, Muwonge J, Ssenono

V, Bahendeka SK. The Epidemiology of Hypertension in Uganda: Findings from the

National Non-Communicable Diseases Risk Factor Survey. PLoS One. 2015 Sep

25;10(9):e0138991. doi: 10.1371/journal.pone.0138991. PMID: 26406462; PMCID:

PMC4583385.

291: Mayanja BN, Kasamba I, Levin J, Namakoola I, Kazooba P, Were J, Kaleebu P,

Munderi P; CoLTART study team. COHORT PROFILE: The Complications of Long-Term

Antiretroviral Therapy study in Uganda (CoLTART), a prospective clinical cohort.

AIDS Res Ther. 2017 May 4;14:26. doi: 10.1186/s12981-017-0154-y. PMID: 28484508;

PMCID: PMC5418696.

292: Shavadia J, Yonga G, Mwanzi S, Jinah A, Moriasi A, Otieno H. Clinical

characteristics and outcomes of atrial fibrillation and flutter at the Aga Khan

University Hospital, Nairobi. Cardiovasc J Afr. 2013 Mar;24(2):6-9. doi:

10.5830/CVJA-2012-064. PMID: 23612946; PMCID: PMC3734872.

293: Musinguzi G, Van Geertruyden JP, Bastiaens H, Nuwaha F. Uncontrolled

hypertension in Uganda: a comparative cross-sectional study. J Clin Hypertens

(Greenwich). 2015 Jan;17(1):63-9. doi: 10.1111/jch.12371. Epub 2014 Jul 15.

PMID: 25040204.

294: Njelekela M, Negishi H, Nara Y, Tomohiro M, Kuga S, Noguchi T, Kanda T,

Yamori M, Mashalla Y, Jian Liu L, Mtabaji J, Ikeda K, Yamori Y. Cardiovascular

risk factors in Tanzania: a revisit. Acta Trop. 2001 Jun 22;79(3):231-9. doi:

10.1016/s0001-706x(01)00134-6. PMID: 11412807.

295: Pai-Dhungat JV, Parikh F. Brazilian viper and BP. control. Stamps issued on

kidneys and hypertension--Uganda, 1978; John R Vane--Micronesia 2001 and

Bothrops jararaca viper--Brazil, 2001. J Assoc Physicians India. 2008

Dec;56:997. PMID: 19322984.

296: Bloomfield GS, Kirwa K, Agarwal A, Eliot MN, Alenezi F, Carter EJ, Foster

MC, Kimaiyo S, Lumsden R, Menya D, Mitter SS, Velazquez EJ, Vedanthan R,

Wellenius GA. Effects of a Cookstove Intervention on Cardiac Structure, Cardiac

Function, and Blood Pressure in Western Kenya. J Am Soc Echocardiogr. 2019

Mar;32(3):427-430. doi: 10.1016/j.echo.2018.11.013. Epub 2019 Jan 18. PMID:

30665729.

297: Nabaweesi-Batuka J, Kitunguu PK, Kiboi JG. Pattern of Cerebral Aneurysms in

a Kenyan Population as Seen at an Urban Hospital. World Neurosurg. 2016

Mar;87:255-65. doi: 10.1016/j.wneu.2015.09.061. Epub 2015 Oct 8. PMID: 26456068.

298: Ogeng'o JA, Gatonga PM, Olabu BO, Nyamweya DK, Ong'era D. Pattern of

congestive heart failure in a Kenyan paediatric population. Cardiovasc J Afr.

2013 May;24(4):117-20. doi: 10.5830/CVJA-2013-015. PMID: 24217041; PMCID:

PMC3734873.

299: Ogeng'o JA, Otieno BO, Kilonzi J, Sinkeet SR, Muthoka JM. Intracranial

aneurysms in an African country. Neurol India. 2009 Sep-Oct;57(5):613-6. doi:

10.4103/0028-3886.57816. PMID: 19934561.

300: Munube D, Katabira E, Ndeezi G, Joloba M, Lhatoo S, Sajatovic M, Tumwine

JK. Prevalence of stroke in children admitted with sickle cell anaemia to Mulago

Hospital. BMC Neurol. 2016 Sep 17;16:175. doi: 10.1186/s12883-016-0704-2. PMID:

27639979; PMCID: PMC5026778.

301: Katalambula LK, Meyer DN, Ngoma T, Buza J, Mpolya E, Mtumwa AH, Petrucka P.

Dietary pattern and other lifestyle factors as potential contributors to

hypertension prevalence in Arusha City, Tanzania: a population-based descriptive

study. BMC Public Health. 2017 Aug 16;17(1):659. doi: 10.1186/s12889-017-4679-8.

PMID: 28814286; PMCID: PMC5559771.

302: Chepchirchir A, Jaoko W, Nyagol J. Risk indicators and effects of

hypertension on HIV/AIDS disease progression among patients seen at Kenyatta

hospital HIV care center. AIDS Care. 2018 May;30(5):544-550. doi:

10.1080/09540121.2017.1384533. Epub 2017 Oct 9. PMID: 28990417.

303: Nuwaha F, Musinguzi G. Use of alternative medicine for hypertension in

Buikwe and Mukono districts of Uganda: a cross sectional study. BMC Complement

Altern Med. 2013 Nov 4;13:301. doi: 10.1186/1472-6882-13-301. PMID: 24180548;

PMCID: PMC3819467.

304: Adinan J, Manongi R, Temu GA, Kapologwe N, Marandu A, Wajanga B, Dika H,

Maongezi S, Laizer S, Manyuti R, Nassir RA, Renju J, Todd J. Preparedness of

health facilities in managing hypertension & diabetes mellitus in Kilimanjaro,

Tanzania: a cross sectional study. BMC Health Serv Res. 2019 Jul 31;19(1):537.

doi: 10.1186/s12913-019-4316-6. PMID: 31366384; PMCID: PMC6670222.

305: Paddick SM, Longdon A, Kisoli A, Gray WK, Dotchin CL, Jusabani A, Iqbal A,

Hughes J, Teodorczuk A, Chaote P, Walker RW. The prevalence of dementia subtypes

in rural Tanzania. Am J Geriatr Psychiatry. 2014 Dec;22(12):1613-22. doi:

10.1016/j.jagp.2014.02.004. Epub 2014 Feb 19. PMID: 25134968.

306: Murphy GA, Asiki G, Ekoru K, Nsubuga RN, Nakiyingi-Miiro J, Young EH,

Seeley J, Sandhu MS, Kamali A. Sociodemographic distribution of non-communicable

disease risk factors in rural Uganda: a cross-sectional study. Int J Epidemiol.

2013 Dec;42(6):1740-53. doi: 10.1093/ije/dyt184. Epub 2013 Nov 4. Erratum in:

Int J Epidemiol. 2016 Dec 1;45(6):2209. PMID: 24191304; PMCID: PMC4234905.

307: Wesonga R, Guwatudde D, Bahendeka SK, Mutungi G, Nabugoomu F, Muwonge J.

Burden of cumulative risk factors associated with non-communicable diseases

among adults in Uganda: evidence from a national baseline survey. Int J Equity

Health. 2016 Dec 1;15(1):195. doi: 10.1186/s12939-016-0486-6. PMID: 27905949;

PMCID: PMC5133748.

308: Namuyonga J, Lubega S, Musiime V, Lwabi P, Lubega I. Cardiac Dysfunction

Among Ugandan HIV-infected Children on Antiretroviral Therapy. Pediatr Infect

Dis J. 2016 Mar;35(3):e85-8. doi: 10.1097/INF.0000000000000997. PMID: 26569195;

PMCID: PMC4752406.

309: Kaddumukasa M, Kayima J, Nakibuuka J, Blixen C, Welter E, Katabira E,

Sajatovic M. Modifiable lifestyle risk factors for stroke among a high risk

hypertensive population in Greater Kampala, Uganda; a cross-sectional study. BMC

Res Notes. 2017 Dec 4;10(1):675. doi: 10.1186/s13104-017-3009-7. PMID: 29202881;

PMCID: PMC5716002.

310: Walker RW, Jusabani A, Aris E, Gray WK, Unwin N, Swai M, Alberti G, Mugusi

F. Stroke risk factors in an incident population in urban and rural Tanzania: a

prospective, community-based, case-control study. Lancet Glob Health. 2013

Nov;1(5):e282-8. doi: 10.1016/S2214-109X(13)70068-8. PMID: 24748275; PMCID:

PMC3986030.

311: Mugwano I, Kaddumukasa M, Mugenyi L, Kayima J, Ddumba E, Sajatovic M, Sila

C, DeGeorgia M, Katabira E. Poor drug adherence and lack of awareness of

hypertension among hypertensive stroke patients in Kampala, Uganda: a cross

sectional study. BMC Res Notes. 2016 Jan 2;9:3. doi: 10.1186/s13104-015-1830-4.

PMID: 26725042; PMCID: PMC4698320.

312: Nyamu DG, Guantai AN, Osanjo GO, Godman B, Aklillu E. Profiles of patients

on warfarin anticoagulation therapy in a leading tertiary referral hospital in

Kenya; findings and implications for Kenya. Expert Rev Cardiovasc Ther. 2020

Mar;18(3):165-173. doi: 10.1080/14779072.2020.1734452. Epub 2020 Mar 2. PMID:

32090626.

313: Morbach S, Lutale JK, Viswanathan V, Möllenberg J, Ochs HR, Rajashekar S,

Ramachandran A, Abbas ZG. Regional differences in risk factors and clinical

presentation of diabetic foot lesions. Diabet Med. 2004 Jan;21(1):91-5. doi:

10.1046/j.1464-5491.2003.01069.x. PMID: 14706061.

314: Ogeng'o JA, Olabu BO, Kilonzi JP. Pattern of aortic aneurysms in an African

country. J Thorac Cardiovasc Surg. 2010 Oct;140(4):797-800. doi:

10.1016/j.jtcvs.2009.11.023. Epub 2010 Feb 21. PMID: 20176370.

315: Beaton A, Okello E, Lwabi P, Mondo C, McCarter R, Sable C. Echocardiography

screening for rheumatic heart disease in Ugandan schoolchildren. Circulation.

2012 Jun 26;125(25):3127-32. doi: 10.1161/CIRCULATIONAHA.112.092312. Epub 2012

May 24. PMID: 22626741.

316: Yego F, D'Este C, Byles J, Williams JS, Nyongesa P. Risk factors for

maternal mortality in a Tertiary Hospital in Kenya: a case control study. BMC

Pregnancy Childbirth. 2014 Jan 22;14:38. doi: 10.1186/1471-2393-14-38. PMID:

24447854; PMCID: PMC3904405.

317: Vedanthan R, Kamano JH, Horowitz CR, Ascheim D, Velazquez EJ, Kimaiyo S,

Fuster V. Nurse management of hypertension in rural western Kenya:

implementation research to optimize delivery. Ann Glob Health. 2014 Jan-

Feb;80(1):5-12. doi: 10.1016/j.aogh.2013.12.002. Epub 2013 Dec 25. PMID:

24751560; PMCID: PMC4036099.

318: Diarz EJ, Leyaro BJ, Kivuyo SL, Ngowi BJ, Msuya SE, Mfinanga SG, Bonfoh B,

Mahande MJ. Red meat consumption and its association with hypertension and

hyperlipidaemia among adult Maasai pastoralists of Ngorongoro Conservation Area,

Tanzania. PLoS One. 2020 Jun 1;15(6):e0233777. doi:

10.1371/journal.pone.0233777. PMID: 32479535; PMCID: PMC7263614.

319: Peck RN, Shedafa R, Kalluvya S, Downs JA, Todd J, Suthanthiran M,

Fitzgerald DW, Kataraihya JB. Hypertension, kidney disease, HIV and

antiretroviral therapy among Tanzanian adults: a cross-sectional study. BMC Med.

2014 Jul 29;12:125. doi: 10.1186/s12916-014-0125-2. PMID: 25070128; PMCID:

PMC4243281.

320: Asayama K, Ohkubo T. Unattended Automated Measurements: Office and Out-of-

Office Blood Pressures Affected by Medical Staff and Environment. Hypertension.

2019 Dec;74(6):1294-1296. doi: 10.1161/HYPERTENSIONAHA.119.13753. Epub 2019 Oct

7. PMID: 31587586.

321: Jowi JO, Mativo PM. Pathological sub-types, risk factors and outcome of

stroke at the Nairobi Hospital, Kenya. East Afr Med J. 2008 Dec;85(12):572-81.

doi: 10.4314/eamj.v85i12.43535. PMID: 19413212.

322: Kiboi JG, Kitunguu PK, Angwenyi P, Mbuthia F, Sagina LS. Predictors of

functional recovery in African patients with traumatic intracranial hematomas.

World Neurosurg. 2011 May-Jun;75(5-6):586-91. doi: 10.1016/j.wneu.2010.05.041.

PMID: 21704911.

323: Namuyonga J, Lwabi PS, Omagino J, Yacoub M, Mocumbi AO. Feasibility of

south-south collaboration in Africa: the Uganda-Mozambique perspective.

Cardiovasc J Afr. 2018 Jul/Aug;29(4):262-263. doi: 10.5830/CVJA-2018-030. PMID:

30204223.

324: Engelman D, Okello E, Beaton A, Selnow G, Remenyi B, Watson C, Longenecker

CT, Sable C, Steer AC. Evaluation of Computer-Based Training for Health Workers

in Echocardiography for RHD. Glob Heart. 2017 Mar;12(1):17-23.e8. doi:

10.1016/j.gheart.2015.12.001. Epub 2016 Mar 16. PMID: 26994642.

325: Beaton A, Lu JC, Aliku T, Dean P, Gaur L, Weinberg J, Godown J, Lwabi P,

Mirembe G, Okello E, Reese A, Shrestha-Astudillo A, Bradley-Hewitt T, Scheel J,

Webb C, McCarter R, Ensing G, Sable C. The utility of handheld echocardiography

for early rheumatic heart disease diagnosis: a field study. Eur Heart J

Cardiovasc Imaging. 2015 May;16(5):475-82. doi: 10.1093/ehjci/jeu296. Epub 2015

Jan 5. PMID: 25564396; PMCID: PMC4542771.

326: Walker RW, McLarty DG, Masuki G, Kitange HM, Whiting D, Moshi AF, Massawe

JG, Amaro R, Mhina A, Alberti KG. Age specific prevalence of impairment and

disability relating to hemiplegic stroke in the Hai District of northern

Tanzania. Adult Morbidity and Mortality Project. J Neurol Neurosurg Psychiatry.

2000 Jun;68(6):744-9. doi: 10.1136/jnnp.68.6.744. PMID: 10811698; PMCID:

PMC1736964.

327: Nyamu PN, Otieno CF, Amayo EO, McLigeyo SO. Risk factors and prevalence of

diabetic foot ulcers at Kenyatta National Hospital, Nairobi. East Afr Med J.

2003 Jan;80(1):36-43. doi: 10.4314/eamj.v80i1.8664. PMID: 12755240.

328: Serem GK, Newton CR, Kariuki SM. Incidence, causes and phenotypes of acute

seizures in Kenyan children post the malaria-decline period. BMC Neurol. 2015

Oct 6;15:180. doi: 10.1186/s12883-015-0444-8. PMID: 26444670; PMCID: PMC4596303.

329: Kimando MW, Otieno FCF, Ogola EN, Mutai K. Adequacy of control of

cardiovascular risk factors in ambulatory patients with type 2 diabetes

attending diabetes out-patients clinic at a county hospital, Kenya. BMC Endocr

Disord. 2017 Dec 1;17(1):73. doi: 10.1186/s12902-017-0223-1. PMID: 29191193;

PMCID: PMC5709860.

330: Siedner MJ, Kim JH, Nakku RS, Hemphill L, Triant VA, Haberer JE, Martin JN,

Boum Y 2nd, Kwon DS, Tsai AC, Hunt PW, Okello S, Bangsberg DR. HIV infection and

arterial stiffness among older-adults taking antiretroviral therapy in rural

Uganda. AIDS. 2016 Feb 20;30(4):667-70. doi: 10.1097/QAD.0000000000000992. PMID:

26636926; PMCID: PMC4733585.

331: Piot P, Aerts A, Wood DA, Lamptey P, Oti S, Connell K, Dorairaj P, Boufford

JI, Caldwell A, Perel P. Innovating healthcare delivery to address

noncommunicable diseases in low-income settings: the example of hypertension.

Future Cardiol. 2016 Jul;12(4):401-3. doi: 10.2217/fca-2016-0032. Epub 2016 Jun

13. PMID: 27291058.

332: Jenson A, Omar AL, Omar MA, Rishad AS, Khoshnood K. Assessment of

hypertension control in a district of Mombasa, Kenya. Glob Public Health.

2011;6(3):293-306. doi: 10.1080/17441692.2010.510478. PMID: 20845123.

333: Winnicki M, Somers VK, Accurso V, Phillips BG, Puato M, Palatini P,

Pauletto P. Fish-rich diet, leptin, and body mass. Circulation. 2002 Jul

16;106(3):289-91. doi: 10.1161/01.cir.0000025241.01418.4d. PMID: 12119240.

334: Michael D, Kezakubi D, Juma A, Todd J, Reyburn H, Renju J. The feasibility

and acceptability of screening for hypertension in private drug retail outlets:

a pilot study in Mwanza region, Tanzania. Int Health. 2016 Sep;8(5):360-6. doi:

10.1093/inthealth/ihw023. Epub 2016 May 11. PMID: 27170761; PMCID: PMC5039818.

335: Ndwiga C, Odwe G, Pooja S, Ogutu O, Osoti A, E Warren C. Clinical

presentation and outcomes of pre-eclampsia and eclampsia at a national hospital,

Kenya: A retrospective cohort study. PLoS One. 2020 Jun 5;15(6):e0233323. doi:

10.1371/journal.pone.0233323. PMID: 32502144; PMCID: PMC7274433.

336: Nguchu HK, Joshi MD, Otieno CF. Acute coronary syndromes amongst type 2

diabetics with ischaemic electrocardiograms presenting to accident and emergency

department of a Kenyan tertiary institution. East Afr Med J. 2009

Oct;86(10):463-8. doi: 10.4314/eamj.v86i10.54972. PMID: 21650069.

337: Langli Ersdal H, Mduma E, Svensen E, Sundby J, Perlman J. Intermittent

detection of fetal heart rate abnormalities identify infants at greatest risk

for fresh stillbirths, birth asphyxia, neonatal resuscitation, and early

neonatal deaths in a limited-resource setting: a prospective descriptive

observational study at Haydom Lutheran Hospital. Neonatology.

2012;102(3):235-42. doi: 10.1159/000339481. Epub 2012 Aug 15. PMID: 22907583.

338: Lazar JM, Wu X, Shi Q, Kagame A, Cohen M, Binagwaho A, Munyakazi L,

Salciccioli L, Shi D, Anastos K. Arterial wave reflection in HIV-infected and

HIV-uninfected Rwandan women. AIDS Res Hum Retroviruses. 2009 Sep;25(9):877-82.

doi: 10.1089/aid.2008.0269. PMID: 19689195; PMCID: PMC2858930.

339: Ogeng'o JA, Obimbo MM, King'ori J. Pattern of limb amputation in a Kenyan

rural hospital. Int Orthop. 2009 Oct;33(5):1449-53. doi:

10.1007/s00264-009-0810-5. Epub 2009 May 28. PMID: 19475408; PMCID: PMC2899126.

340: Di Maria MV, Hsu HH, Al-Naami G, Gruenwald J, Kirby KS, Kirkham FJ, Cox SE,

Younoszai AK. Left ventricular rotational mechanics in Tanzanian children with

sickle cell disease. J Am Soc Echocardiogr. 2015 Mar;28(3):340-6. doi:

10.1016/j.echo.2014.11.014. Epub 2014 Dec 30. PMID: 25555520; PMCID: PMC4346279.

341: Etyang AO, Warne B, Kapesa S, Munge K, Bauni E, Cruickshank JK, Smeeth L,

Scott JA. Clinical and Epidemiological Implications of 24-Hour Ambulatory Blood

Pressure Monitoring for the Diagnosis of Hypertension in Kenyan Adults: A

Population-Based Study. J Am Heart Assoc. 2016 Dec 15;5(12):e004797. doi:

10.1161/JAHA.116.004797. PMID: 27979807; PMCID: PMC5210452.

342: Zhang W, Mondo C, Okello E, Musoke C, Kakande B, Nyakoojo W, Kayima J,

Freers J. Presenting features of newly diagnosed rheumatic heart disease

patients in Mulago Hospital: a pilot study. Cardiovasc J Afr. 2013

Mar;24(2):28-33. doi: 10.5830/CVJA-2012-076. PMID: 23612950; PMCID: PMC3734881.

343: Saidi H, Ongeti WK, Ogeng'o J. Morphology of human myocardial bridges and

association with coronary artery disease. Afr Health Sci. 2010 Sep;10(3):242-7.

PMID: 21327135; PMCID: PMC3035959.

344: Baker EC, Hezelgrave N, Magesa SM, Edmonds S, de Greeff A, Shennan A.

Introduction of automated blood pressure devices intended for a low resource

setting in rural Tanzania. Trop Doct. 2012 Apr;42(2):101-3. doi:

10.1258/td.2011.110352. PMID: 22431828.

345: Abbas ZG, Lutale JK, Game FL, Jeffcoate WJ. Comparison of four systems of

classification of diabetic foot ulcers in Tanzania. Diabet Med. 2008

Feb;25(2):134-7. doi: 10.1111/j.1464-5491.2007.02308.x. Epub 2008 Jan 19. PMID:

18215177.

346: Kisozi T, Mutebi E, Kisekka M, Lhatoo S, Sajatovic M, Kaddumukasa M,

Nakwagala FN, Katabira E. Prevalence, severity and factors associated with

peripheral neuropathy among newly diagnosed diabetic patients attending Mulago

hospital: a cross-sectional study. Afr Health Sci. 2017 Jun;17(2):463-473. doi:

10.4314/ahs.v17i2.21. PMID: 29062342; PMCID: PMC5637032.

347: Whyte SR. Chronicity and control: framing 'noncommunicable diseases' in

Africa. Anthropol Med. 2012 Apr;19(1):63-74. doi: 10.1080/13648470.2012.660465.

PMID: 22612492.

348: Dewhurst MJ, Adams PC, Gray WK, Dewhurst F, Orega GP, Chaote P, Walker RW.

Strikingly low prevalence of atrial fibrillation in elderly Tanzanians. J Am

Geriatr Soc. 2012 Jun;60(6):1135-40. doi: 10.1111/j.1532-5415.2012.03963.x. Epub

2012 May 30. PMID: 22646732.

349: Abbas ZG, Lutale JK, Bakker K, Baker N, Archibald LK. The 'Step by Step'

Diabetic Foot Project in Tanzania: a model for improving patient outcomes in

less-developed countries. Int Wound J. 2011 Apr;8(2):169-75. doi:

10.1111/j.1742-481X.2010.00764.x. Epub 2011 Jan 25. PMID: 21266010.

350: Smide B. Outcome of foot examinations in Tanzanian and Swedish diabetic

patients, a comparative study. J Clin Nurs. 2009 Feb;18(3):391-8. doi:

10.1111/j.1365-2702.2008.02492.x. PMID: 19191986.

351: Chamie G, Kwarisiima D, Clark TD, Kabami J, Jain V, Geng E, Petersen ML,

Thirumurthy H, Kamya MR, Havlir DV, Charlebois ED; SEARCH Collaboration.

Leveraging rapid community-based HIV testing campaigns for non-communicable

diseases in rural Uganda. PLoS One. 2012;7(8):e43400. doi:

10.1371/journal.pone.0043400. Epub 2012 Aug 20. PMID: 22916256; PMCID:

PMC3423366.

352: Nakimuli A, Elliott AM, Kaleebu P, Moffett A, Mirembe F. Hypertension

persisting after pre-eclampsia: a prospective cohort study at Mulago Hospital,

Uganda. PLoS One. 2013 Dec 31;8(12):e85273. doi: 10.1371/journal.pone.0085273.

PMID: 24392003; PMCID: PMC3877387.

353: Sallo FB, Leung I, Mathenge W, Kyari F, Kuper H, Gilbert CE, Bird AC, Peto

T; MacTel Study Group. The prevalence of type 2 idiopathic macular

telangiectasia in two African populations. Ophthalmic Epidemiol. 2012

Aug;19(4):185-9. doi: 10.3109/09286586.2011.638744. Epub 2012 Feb 24. PMID:

22364548.

354: Ocen D, Kalungi S, Ejoku J, Luggya T, Wabule A, Tumukunde J, Kwizera A.

Prevalence, outcomes and factors associated with adult in hospital cardiac

arrests in a low-income country tertiary hospital: a prospective observational

study. BMC Emerg Med. 2015 Sep 16;15:23. doi: 10.1186/s12873-015-0047-0. PMID:

26376745; PMCID: PMC4574081.

355: Urimubenshi G. Activity limitations and participation restrictions

experienced by people with stroke in Musanze district in Rwanda. Afr Health Sci.

2015 Sep;15(3):917-24. doi: 10.4314/ahs.v15i3.28. PMID: 26957982; PMCID:

PMC4765476.

356: Mugambi-Nturibi E, Otieno CF, Kwasa TO, Oyoo GO, Acharya K. Stratification

of persons with diabetes into risk categories for foot ulceration. East Afr Med

J. 2009 May;86(5):233-9. doi: 10.4314/eamj.v86i5.54195. PMID: 20084992.

357: Halestrap P, Scheenstra S. Outbreak of scurvy in Tana River County, Kenya:

A case report. Afr J Prim Health Care Fam Med. 2018 Oct 25;10(1):e1-e3. doi:

10.4102/phcfm.v10i1.1811. PMID: 30456974; PMCID: PMC6244223.

358: Maro EE, Kaushik R. The role of echocardiography in the management of

patients with congestive heart failure. "Tanzanian experience". Cent Afr J Med.

2009 May-Aug;55(5-8):35-9. doi: 10.4314/cajm.v55i5-8.63638. PMID: 21977826.

359: Mucumbitsi J, Bulwer B, Mutesa L, Ndahindwa V, Semakula M, Rusingiza E,

Arya P, Breakey S, Patton-Bolman C, Kaplan EL. Prevalence of rheumatic valvular

heart disease in Rwandan school children: echocardiographic evaluation using the

World Heart Federation criteria. Cardiovasc J Afr. 2017 Sep/Oct

23;28(5):285-292. doi: 10.5830/CVJA-2017-007. Epub 2017 Mar 1. PMID: 28252675;

PMCID: PMC5730679.

360: Omar FJ, Sheeladevi S, Rani PK, Ning G, Kabona G. Evaluating the

effectiveness of opportunistic eye screening model for people with diabetes

attending diabetes clinic at Mnazi Mmoja Hospital, Zanzibar. BMC Ophthalmol.

2014 Jun 24;14:81. doi: 10.1186/1471-2415-14-81. PMID: 24957576; PMCID:

PMC4078096.

361: Olack B, Wabwire-Mangen F, Smeeth L, Montgomery JM, Kiwanuka N, Breiman RF.

Risk factors of hypertension among adults aged 35-64 years living in an urban

slum Nairobi, Kenya. BMC Public Health. 2015 Dec 17;15:1251. doi:

10.1186/s12889-015-2610-8. PMID: 26679701; PMCID: PMC4683777.

362: Nakibuuka J, Sajatovic M, Nankabirwa J, Ssendikadiwa C, Kalema N, Kwizera

A, Byakika-Tusiime J, Furlan AJ, Kayima J, Ddumba E, Katabira E. Effect of a 72

Hour Stroke Care Bundle on Early Outcomes after Acute Stroke: A Non Randomised

Controlled Study. PLoS One. 2016 May 4;11(5):e0154333. doi:

10.1371/journal.pone.0154333. PMID: 27145035; PMCID: PMC4856379.

363: Chalya PL, Mabula JB, Dass RM, Ngayomela IH, Chandika AB, Mbelenge N,

Gilyoma JM. Major limb amputations: a tertiary hospital experience in

northwestern Tanzania. J Orthop Surg Res. 2012 May 11;7:18. doi:

10.1186/1749-799X-7-18. PMID: 22578187; PMCID: PMC3413574.

364: Mathenge W, Bastawrous A, Peto T, Leung I, Yorston D, Foster A, Kuper H.

Prevalence and correlates of diabetic retinopathy in a population-based survey

of older people in Nakuru, Kenya. Ophthalmic Epidemiol. 2014 Jun;21(3):169-77.

doi: 10.3109/09286586.2014.903982. Epub 2014 Apr 23. PMID: 24758280.

365: Mutimura E, Crowther NJ, Stewart A, Cade WT. The human immunodeficiency

virus and the cardiometabolic syndrome in the developing world: an African

perspective. J Cardiometab Syndr. 2008 Spring;3(2):106-10. doi:

10.1111/j.1559-4572.2008.07584.x. PMID: 18453811; PMCID: PMC4493161.

366: Hansen MB, Abràmoff MD, Folk JC, Mathenge W, Bastawrous A, Peto T. Results

of Automated Retinal Image Analysis for Detection of Diabetic Retinopathy from

the Nakuru Study, Kenya. PLoS One. 2015 Oct 1;10(10):e0139148. doi:

10.1371/journal.pone.0139148. PMID: 26425849; PMCID: PMC4591009.

367: Walker RW, Viney R, Green L, Mawanswila M, Maro VP, Gjertsen C, Godfrey H,

Smailes R, Gray WK. Trends in stroke admissions to a Tanzanian hospital over

four decades: a retrospective audit. Trop Med Int Health. 2015

Oct;20(10):1290-6. doi: 10.1111/tmi.12547. Epub 2015 Jun 1. PMID: 25983015.

368: Adegboye VO, Ogunseyinde AO, Obajimi MO, Brimmo AI, Adebo OA. Superior vena

cava obstruction: diagnosis, management and outcome. East Afr Med J. 2008

Mar;85(3):129-36. PMID: 18663886.

369: Hertz JT, Kweka GL, Manavalan P, Watt MH, Sakita FM. Provider-perceived

barriers to diagnosis and treatment of acute coronary syndrome in Tanzania: a

qualitative study. Int Health. 2020 Feb 12;12(2):148-154. doi:

10.1093/inthealth/ihz061. PMID: 31329876; PMCID: PMC7017879.

370: Parati G, Kilama MO, Faini A, Facelli E, Ochen K, Opira C, Mendis S, Wang

J, Atkins N, O'Brien E. A new solar-powered blood pressure measuring device for

low-resource settings. Hypertension. 2010 Dec;56(6):1047-53. doi:

10.1161/HYPERTENSIONAHA.110.160408. Epub 2010 Nov 8. PMID: 21059998.

371: Epstein D, Petersiel N, Klein E, Marcusohn E, Aviran E, Harel R, Azzam ZS,

Neuberger A, Fuchs L. Pocket-size point-of-care ultrasound in rural Uganda - A

unique opportunity "to see", where no imaging facilities are available. Travel

Med Infect Dis. 2018 May-Jun;23:87-93. doi: 10.1016/j.tmaid.2018.01.001. Epub

2018 Jan 6. PMID: 29317333.

372: Wandabwa J, Doyle P, Paul K, Wandabwa MA, Aziga F. Risk factors for severe

abruptio placenta in Mulago Hospital, Kampala, Uganda. Afr Health Sci. 2005

Dec;5(4):285-90. doi: 10.5555/afhs.2005.5.4.285. PMID: 16615836; PMCID:

PMC1831945.

373: Mwale C, Karimurio J, Njuguna M. Refractive errors in type 2 diabetic

patients. East Afr Med J. 2007 Jun;84(6):259-63. doi: 10.4314/eamj.v84i6.9534.

PMID: 18254467.

374: Lagat DK, DeLong AK, Wellenius GA, Carter EJ, Bloomfield GS, Velazquez EJ,

Hogan J, Kimaiyo S, Sherman CB. Factors associated with isolated right heart

failure in women: a pilot study from western Kenya. Glob Heart. 2014

Jun;9(2):249-54. doi: 10.1016/j.gheart.2014.04.003. PMID: 25667096; PMCID:

PMC4405788.

375: Kwan GF, Bukhman AK, Miller AC, Ngoga G, Mucumbitsi J, Bavuma C, Dusabeyezu

S, Rich ML, Mutabazi F, Mutumbira C, Ngiruwera JP, Amoroso C, Ball E, Fraser HS,

Hirschhorn LR, Farmer P, Rusingiza E, Bukhman G. A simplified echocardiographic

strategy for heart failure diagnosis and management within an integrated

noncommunicable disease clinic at district hospital level for sub-Saharan

Africa. JACC Heart Fail. 2013 Jun;1(3):230-6. doi: 10.1016/j.jchf.2013.03.006.

Epub 2013 Jun 3. PMID: 24621875.

376: Tippett B, Wilson J, Shepherd J, Cutler R, Webster P. Operation Open Heart

--Rwanda. Heart Lung Circ. 2008;17 Suppl 4:S82-3. doi:

10.1016/j.hlc.2008.08.011. Epub 2008 Oct 16. PMID: 18929504.

377: Mwebaze RM, Kibirige D. Peripheral arterial disease among adult diabetic

patients attending a large outpatient diabetic clinic at a national referral

hospital in Uganda: a descriptive cross sectional study. PLoS One. 2014 Aug

18;9(8):e105211. doi: 10.1371/journal.pone.0105211. PMID: 25133533; PMCID:

PMC4136814.

378: Walker RW, Jusabani A, Aris E, Gray WK, Whiting D, Kabadi G, Mugusi F, Swai

M, Alberti G, Unwin N. Post-stroke case fatality within an incident population

in rural Tanzania. J Neurol Neurosurg Psychiatry. 2011 Sep;82(9):1001-5. doi:

10.1136/jnnp.2010.231944. Epub 2011 Mar 8. PMID: 21386108.

379: Aliku TO, Lubega S, Lwabi P, Oketcho M, Omagino JO, Mwambu T. Outcome of

patients undergoing open heart surgery at the Uganda heart institute, Mulago

hospital complex. Afr Health Sci. 2014 Dec;14(4):946-52. doi:

10.4314/ahs.v14i4.25. PMID: 25834506; PMCID: PMC4370076.

380: Hotez PJ. Linking Tropical Infections to Hypertension: New Comorbid Disease

Paradigms in Our Era of "Blue Marble Health". J Am Heart Assoc. 2019 Mar

19;8(6):e03984. doi: 10.1161/JAHA.119.012313. PMID: 30879371; PMCID: PMC6475037.

381: Njenga FG, Kamotho CG, Joshi MD, Gikonyo DK, Wanyoike M. Coronary artery

disease and symptoms of depression in a Kenyan population. East Afr Med J. 2004

Dec;81(12):611-5. doi: 10.4314/eamj.v81i12.9244. PMID: 15868974.

382: Bovet P, Gervasoni JP, Ross AG, Mkamba M, Mtasiwa DM, Lengeler C, Burnier

M, Paccaud F. Assessing the prevalence of hypertension in populations: are we

doing it right? J Hypertens. 2003 Mar;21(3):509-17. doi:

10.1097/00004872-200303000-00016. PMID: 12640244.

383: Walker RW, Jusabani A, Aris E, Gray WK, Mugusi F, Swai M, Alberti KG, Unwin

N. Correlates of short- and long-term case fatality within an incident stroke

population in Tanzania. S Afr Med J. 2012 Dec 11;103(2):107-12. doi:

10.7196/samj.5793. PMID: 23374304.

384: Mecha JO, Kubo EN, Odhiambo CO, Kinoti FG, Njau K, Yonga G, Ogola EN.

Burden of prehypertension among adults in Kenya: a retrospective analysis of

findings from the Healthy Heart Africa (HHA) Programme. BMC Public Health. 2020

Mar 3;20(1):281. doi: 10.1186/s12889-020-8363-z. PMID: 32126994; PMCID:

PMC7055018.

385: Mocumbi AO. Rheumatic Heart Disease: Is Continuum of Care Achievable in

Africa? Circ Cardiovasc Qual Outcomes. 2017 Nov;10(11):e004304. doi:

10.1161/CIRCOUTCOMES.117.004304. PMID: 29133471.

386: Muthuuri JM. Characteristics of patients with diabetic foot in Mombasa,

Kenya. East Afr Med J. 2007 Jun;84(6):251-8. doi: 10.4314/eamj.v84i6.9533. PMID:

18254466.

387: Lubega S, Zirembuzi GW, Lwabi P. Heart disease among children with HIV/AIDS

attending the paediatric infectious disease clinic at Mulago Hospital. Afr

Health Sci. 2005 Sep;5(3):219-26. doi: 10.5555/afhs.2005.5.3.219. PMID:

16245992; PMCID: PMC1831930.

388: Achwoka D, Oyugi JO, Mutave R, Munywoki P, Achia T, Akolo M, Muriuki F,

Muthui M, Kimani J. High prevalence of non-communicable diseases among key

populations enrolled at a large HIV prevention & treatment program in Kenya.

PLoS One. 2020 Jul 2;15(7):e0235606. doi: 10.1371/journal.pone.0235606. PMID:

32614906; PMCID: PMC7332043.

389: Marshall SL, Edidin DV, Arena VC, Becker DJ, Bunker CH, Gishoma C, Gishoma

F, LaPorte RE, Kaberuka V, Ogle G, Sibomana L, Orchard TJ. Glucose control in

Rwandan youth with type 1 diabetes following establishment of systematic, HbA1c

based, care and education. Diabetes Res Clin Pract. 2015 Jan;107(1):113-22. doi:

10.1016/j.diabres.2014.09.045. Epub 2014 Oct 7. PMID: 25458328; PMCID:

PMC4300260.

390: Bloomfield GS, Hogan JW, Keter A, Holland TL, Sang E, Kimaiyo S, Velazquez

EJ. Blood pressure level impacts risk of death among HIV seropositive adults in

Kenya: a retrospective analysis of electronic health records. BMC Infect Dis.

2014 May 22;14:284. doi: 10.1186/1471-2334-14-284. PMID: 24886474; PMCID:

PMC4046023.

391: Muli G, Rhoda A. Quality of life amongst young adults with stroke living in

Kenya. Afr Health Sci. 2013 Sep;13(3):632-8. doi: 10.4314/ahs.v13i3.16. PMID:

24250300; PMCID: PMC3824418.

392: Jamal N, Rajhy M, Bapumia M. Painless acute myocardial infarction on Mount

Kilimanjaro. BMJ Case Rep. 2016 Mar 17;2016:bcr2015214038. doi:

10.1136/bcr-2015-214038. PMID: 26989121; PMCID: PMC4800208.

393: Abbas ZG, Lutale J, Gill GV, Archibald LK. Tropical diabetic hand syndrome:

risk factors in an adult diabetes population. Int J Infect Dis. 2001;5(1):19-23.

doi: 10.1016/s1201-9712(01)90043-8. PMID: 11285154.

394: Onyambu CK, Amayo E, Kitonyi JM. CLINICAL FEATURES AND PATTERNS OF IMAGING

IN CEREBRAL VENOUS SINUS THROMBOSIS AT KENYATTA NATIONAL HOSPITAL. East Afr Med

J. 2013 Sep;90(9):297-304. PMID: 26862647.

395: Birbeck GL, Sposato LA. Hypertension prevention: In need of a grain of salt

knowledge. Neurology. 2016 Sep 20;87(12):1192-3. doi:

10.1212/WNL.0000000000003136. Epub 2016 Aug 24. PMID: 27558373.

396: Chillo P, Lwakatare J, Rieck AE, Lutale J, Gerdts E. Prevalence and

covariates of abnormal left ventricular geometry in never-treated hypertensive

patients in Tanzania. Blood Press. 2014 Feb;23(1):31-8. doi:

10.3109/08037051.2013.791415. Epub 2013 May 31. PMID: 23721542.

397: Centers for Disease Control and Prevention (CDC). Tropical diabetic hand

syndrome--Dar es Salaam, Tanzania, 1998-2002. MMWR Morb Mortal Wkly Rep. 2002

Nov 1;51(43):969-70. PMID: 12433020.

398: Gyagenda JO, Ddumba E, Odokonyero R, Kaddumukasa M, Sajatovic M, Smyth K,

Katabira E. Post-stroke depression among stroke survivors attending two

hospitals in Kampala Uganda. Afr Health Sci. 2015 Dec;15(4):1220-31. doi:

10.4314/ahs.v15i4.22. PMID: 26958024; PMCID: PMC4765432.

399: Senga J, Rusingiza E, Mucumbitsi J, Binagwaho A, Suys B, Lys C, Carbonez K,

Ovaert C, Sluysmans T. Catheter interventions in congenital heart disease

without regular catheterization laboratory equipment: the chain of hope

experience in Rwanda. Pediatr Cardiol. 2013 Jan;34(1):39-45. doi:

10.1007/s00246-012-0378-5. Epub 2012 May 27. PMID: 22644416.

400: Sawers N. Evidence-Based Medicine vs Traditional Healers in Africa. JAMA

Ophthalmol. 2016 Oct 1;134(10):1085-1086. doi: 10.1001/jamaophthalmol.2016.2493.

PMID: 27491011.

401: Walker RW, Wakefield K, Gray WK, Jusabani A, Swai M, Mugusi F. Case-

fatality and disability in the Tanzanian Stroke Incidence Project cohort. Acta

Neurol Scand. 2016 Jan;133(1):49-54. doi: 10.1111/ane.12422. Epub 2015 May 5.

PMID: 25939728; PMCID: PMC4737228.

402: Kleczka B, Musiega A, Rabut G, Wekesa P, Mwaniki P, Marx M, Kumar P. Rubber

stamp templates for improving clinical documentation: A paper-based, m-Health

approach for quality improvement in low-resource settings. Int J Med Inform.

2018 Jun;114:121-129. doi: 10.1016/j.ijmedinf.2017.10.014. Epub 2017 Oct 23.

PMID: 29107565; PMCID: PMC6997026.

403: Rule ARL, Maina E, Cheruiyot D, Mueri P, Simmons JM, Kamath-Rayne BD. Using

quality improvement to decrease birth asphyxia rates after 'Helping Babies

Breathe' training in Kenya. Acta Paediatr. 2017 Oct;106(10):1666-1673. doi:

10.1111/apa.13940. Epub 2017 Jul 14. PMID: 28580692.

404: Stephens JH, Alizadeh F, Bamwine JB, Baganizi M, Chaw GF, Yao Cohen M,

Patel A, Schaefle KJ, Mangat JS, Mukiza J, Paccione GA. Managing hypertension in

rural Uganda: Realities and strategies 10 years of experience at a district

hospital chronic disease clinic. PLoS One. 2020 Jun 5;15(6):e0234049. doi:

10.1371/journal.pone.0234049. PMID: 32502169; PMCID: PMC7274420.

405: Dewhurst MJ, Dewhurst F, Gray WK, Chaote P, Orega GP, Walker RW. The high

prevalence of hypertension in rural-dwelling Tanzanian older adults and the

disparity between detection, treatment and control: a rule of sixths? J Hum

Hypertens. 2013 Jun;27(6):374-80. doi: 10.1038/jhh.2012.59. Epub 2012 Dec 13.

PMID: 23235367.

406: Edward A, Hoffmann L, Manase F, Matsushita K, Pariyo GW, Brady TM, Appel

LJ. An exploratory study on the quality of patient screening and counseling for

hypertension management in Tanzania. PLoS One. 2020 Jan 16;15(1):e0227439. doi:

10.1371/journal.pone.0227439. PMID: 31945075; PMCID: PMC6964881.

407: Ismail Y, Andia I, Byaruhanga S, Shaw MR, Mathieson PW, Wilde P.

Echocardiographic features of cardiac failure in Uganda. Trop Doct. 2007

Oct;37(4):267-8. doi: 10.1258/004947507782332900. PMID: 17988510.

408: Awori MN, Ogendo SW, Gitome SW, Ong'uti SK, Obonyo NG. Management pathway

for congenital heart disease at Kenyatta National Hospital, Nairobi. East Afr

Med J. 2007 Jul;84(7):312-7. doi: 10.4314/eamj.v84i7.9585. PMID: 17886424.

409: Gulam-Abbas Z, Lutale JK, Morbach S, Archibald LK. Clinical outcome of

diabetes patients hospitalized with foot ulcers, Dar es Salaam, Tanzania. Diabet

Med. 2002 Jul;19(7):575-9. doi: 10.1046/j.1464-5491.2002.00740.x. PMID:

12099961.

410: Teteli R, Uwineza A, Butera Y, Hitayezu J, Murorunkwere S, Umurerwa L,

Ndinkabandi J, Hellin AC, Jamar M, Caberg JH, Muganga N, Mucumbitsi J, Rusingiza

EK, Mutesa L. Pattern of congenital heart diseases in Rwandan children with

genetic defects. Pan Afr Med J. 2014 Sep 25;19:85. doi:

10.11604/pamj.2014.19.85.3428. PMID: 25722758; PMCID: PMC4335284.

411: Zhang W, Okello E, Nyakoojo W, Lwabi P, Mondo CK. Proportion of patients in

the Uganda rheumatic heart disease registry with advanced disease requiring

urgent surgical interventions. Afr Health Sci. 2015 Dec;15(4):1182-8. doi:

10.4314/ahs.v15i4.17. PMID: 26958019; PMCID: PMC4765392.

412: Silangei LK, Maro VP, Diefenthal H, Kapanda G, Dewhurst M, Mwandolela H,

Hamel B. Assessment of left ventricular geometrical patterns and function among

hypertensive patients at a tertiary hospital, Northern Tanzania. BMC Cardiovasc

Disord. 2012 Nov 23;12:109. doi: 10.1186/1471-2261-12-109. PMID: 23173763;

PMCID: PMC3528419.

413: Aliku T, Sable C, Scheel A, Tompsett A, Lwabi P, Okello E, McCarter R,

Summar M, Beaton A. Targeted Echocardiographic Screening for Latent Rheumatic

Heart Disease in Northern Uganda: Evaluating Familial Risk Following

Identification of an Index Case. PLoS Negl Trop Dis. 2016 Jun 13;10(6):e0004727.

doi: 10.1371/journal.pntd.0004727. PMID: 27294545; PMCID: PMC4905680.

414: Twagirumukiza M, Nkeramihigo E, Seminega B, Gasakure E, Boccara F, Barbaro

G. Prevalence of dilated cardiomyopathy in HIV-infected African patients not

receiving HAART: a multicenter, observational, prospective, cohort study in

Rwanda. Curr HIV Res. 2007 Jan;5(1):129-37. doi: 10.2174/157016207779316288.

PMID: 17266564.

415: Kuule JK, Seremba E, Freers J. Anaemia among patients with congestive

cardiac failure in Uganda - its impact on treatment outcomes. S Afr Med J. 2009

Dec 7;99(12):876-80. PMID: 20459998.

416: Bimenya GS, Byarugaba W, Kalungi S, Mayito J, Mugabe K, Makabayi R, Ayebare

E, Wanzira H, Muhame M. Blood pressure profiles among Makerere University

undergraduate students. Afr Health Sci. 2005 Jun;5(2):99-106. PMID: 16006215;

PMCID: PMC1831913.

417: Nguyen NW. Micro-Costing Estimation of Workforce Needs: Will That Work for

Cardiac Services in Kenya? World J Pediatr Congenit Heart Surg. 2019

May;10(3):328-329. doi: 10.1177/2150135119843067. PMID: 31084300.

418: Chillo P, Bakari M, Lwakatare J. Echocardiographic diagnoses in HIV-

infected patients presenting with cardiac symptoms at Muhimbili National

Hospital in Dar es Salaam, Tanzania. Cardiovasc J Afr. 2012 Mar;23(2):90-7. doi:

10.5830/CVJA-2011-060. Epub 2012 Feb 13. PMID: 22331234; PMCID: PMC3721886.

419: Yuko-Jowi C, Bakari M. Echocardiographic patterns of juvenile rheumatic

heart disease at the Kenyatta National Hospital, Nairobi. East Afr Med J. 2005

Oct;82(10):514-9. doi: 10.4314/eamj.v82i10.9349. PMID: 16450679.

420: Olang PR, Wamalwa DC, Omondi-Ogutu. MATERNAL HYPOTENSION AND NEONATAL

ACIDAEMIA DURING CAESEREAN DELIVERY UNDER SPINAL ANAESTHESIA. East Afr Med J.

2012 Oct;89(10):317-21. PMID: 26852440.

421: Maro EE, Janabi M, Kaushik R. Clinical and echocardiographic study of

hypertrophic cardiomyopathy in Tanzania. Trop Doct. 2006 Oct;36(4):225-7. doi:

10.1258/004947506778604904. PMID: 17034699.

422: Ellis J, Martin R, Wilde P, Tometzki A, Senkungu J, Nansera D.

Echocardiographic, chest X-ray and electrocardiogram findings in children

presenting with heart failure to a Ugandan paediatric ward. Trop Doct. 2007

Jul;37(3):149-50. doi: 10.1258/004947507781524665. PMID: 17716499.

423: Qureshi ZP. Current management of hypertensive disease in pregnancy. East

Afr Med J. 2002 Apr;79(4):169-71. PMID: 12625667.

424: Urimubenshi G, Rhoda A. Environmental barriers experienced by stroke

patients in Musanze district in Rwanda: a descriptive qualitative study. Afr

Health Sci. 2011 Sep;11(3):398-405. PMID: 22275930; PMCID: PMC3261026.

425: Chin JH. Letter re: Influence of sodium consumption and associated

knowledge on poststroke hypertension in Uganda. Neurology. 2017 Mar

14;88(11):1103. doi: 10.1212/WNL.0000000000003726. PMID: 28289170.

426: Peck R, Mghamba J, Vanobberghen F, Kavishe B, Rugarabamu V, Smeeth L, Hayes

R, Grosskurth H, Kapiga S. Preparedness of Tanzanian health facilities for

outpatient primary care of hypertension and diabetes: a cross-sectional survey.

Lancet Glob Health. 2014 May;2(5):e285-92. doi: 10.1016/S2214-109X(14)70033-6.

Erratum in: Lancet Glob Health. 2014 Sep;2(9):511. PMID: 24818084; PMCID:

PMC4013553.

427: Matuja SS, Munseri P, Khanbhai K. The burden and outcomes of stroke in

young adults at a tertiary hospital in Tanzania: a comparison with older adults.

BMC Neurol. 2020 May 25;20(1):206. doi: 10.1186/s12883-020-01793-2. PMID:

32450825; PMCID: PMC7247244.

428: Mwachaka PM, Obonyo NG, Mutiso BK, Ranketi S, Mwang'ombe N.

Ventriculoperitoneal shunt complications: a three-year retrospective study in a

Kenyan national teaching and referral hospital. Pediatr Neurosurg.

2010;46(1):1-5. doi: 10.1159/000314050. Epub 2010 May 5. PMID: 20453556.

429: Kaplan EL, Bulwer B, Adams D. Low Incidence of Congenital Bicuspid Aortic

Valve in Sub-Saharan African Children. J Am Soc Echocardiogr. 2017

Sep;30(9):932-933. doi: 10.1016/j.echo.2017.05.015. Epub 2017 Jul 6. PMID:

28688855.

430: Mangeni F, Kawooya MG, Kiguli-Malwadde E, Ssali F. Sonography and risk

factors for lower limb deep venous thrombosis at Mulago Hospital, Uganda. East

Afr Med J. 2006 Aug;83(8):443-9. doi: 10.4314/eamj.v83i8.9460. PMID: 17153658.

431: Kuga S, Njelekela M, Noguchi T, Kanda T, Yamori M, Sato T, Miki T, Ikeda K,

Nara Y, Mtabaji J. Prevalence of overweight and hypertension in Tanzania:

special emphasis on resting energy expenditure and leptin. Clin Exp Pharmacol

Physiol Suppl. 2002 Oct;(29):S23-6. PMID: 12355911.

432: Agaba DC, Migisha R, Katamba G, Ashaba S. Cardio-metabolic abnormalities

among patients with severe mental illness at a Regional Referral Hospital in

southwestern Uganda. PLoS One. 2020 Jul 17;15(7):e0235956. doi:

10.1371/journal.pone.0235956. PMID: 32678850; PMCID: PMC7367467.

433: Tesfalul MA, Natureeba P, Day N, Thomas O, Gaw SL. Identifying risk factors

for perinatal death at Tororo District Hospital, Uganda: a case-control study.

BMC Pregnancy Childbirth. 2020 Jan 20;20(1):45. doi: 10.1186/s12884-020-2727-3.

PMID: 31959141; PMCID: PMC6972019.

434: Ouma MN, Chemwolo BT, Pastakia S, Christoffersen-Deb A, Washington S. Pilot

study of single-use obstetric emergency medical kits to reduce maternal

mortality. Int J Gynaecol Obstet. 2012 Oct;119(1):49-52. doi:

10.1016/j.ijgo.2012.05.028. Epub 2012 Aug 11. PMID: 22889547.

435: Ogendo SW. Thirty day mortality and related variables in open heart

patients at the Kenyatta National Hospital, Nairobi. East Afr Med J. 2001

Oct;78(10):526-30. doi: 10.4314/eamj.v78i10.8962. PMID: 11921596.

436: Akomea-Agyin C, Galukande M, Mwambu T, Ttendo S, Clarke I. Pioneer human

open heart surgery using cardiopulmonary by pass in Uganda. Afr Health Sci. 2008

Dec;8(4):259-60. PMID: 20589135; PMCID: PMC2887010.

437: Matuja SS, Khanbhai K, Mahawish KM, Munseri P. Stroke mimics in patients

clinically diagnosed with stroke at a tertiary teaching hospital in Tanzania: a

prospective cohort study. BMC Neurol. 2020 Jul 7;20(1):270. doi:

10.1186/s12883-020-01853-7. PMID: 32635888; PMCID: PMC7339381.

438: Arwady MA. A piece of my mind. Collaterals. JAMA. 2010 Jun

9;303(22):2229-30. doi: 10.1001/jama.2010.762. PMID: 20530767.

439: Ogeng'o JA, Olabu BO. Cortical stroke in Kenya. Int J Stroke. 2010

Dec;5(6):517-8. doi: 10.1111/j.1747-4949.2010.00530.x. PMID: 21050415.

440: Awori KO, Atinga JE. Lower limb amputations at the Kenyatta National

Hospital, Nairobi. East Afr Med J. 2007 Mar;84(3):121-6. PMID: 17600981.

441: Kamotho C, Ogola EO, Joshi M, Gikonyo D. Cardiovascular risk factor profile

of black Africans undergoing coronary angiography. East Afr Med J. 2004

Feb;81(2):82-6. doi: 10.4314/eamj.v81i2.9130. PMID: 15125091.

442: Gleason B, Mirembe G, Namuyonga J, Okello E, Lwabi P, Lubega I, Lubega S,

Musiime V, Kityo C, Salata RA, Longenecker CT. Brief Report: Prevalence of

Latent Rheumatic Heart Disease Among HIV-Infected Children in Kampala, Uganda. J

Acquir Immune Defic Syndr. 2016 Feb 1;71(2):196-9. doi:

10.1097/QAI.0000000000000827. PMID: 26413847; PMCID: PMC4712089.

443: Chiolero A, Gervasoni JP, Rwebogora A, Mkamba M, Waeber B, Paccaud F,

Burnier M, Bovet P. Discordant prevalence of hypertension using two different

automated blood pressure measurement devices: a population-based study in Dar es

Salaam (Tanzania). Blood Press Monit. 2004 Apr;9(2):59-64. doi:

10.1097/00126097-200404000-00001. PMID: 15096901.

444: Monsell E. Is CPR always appropriate? A personal perspective from working

in East Africa. Br J Nurs. 2017 May 11;26(9):503-504. doi:

10.12968/bjon.2017.26.9.503. PMID: 28493782.

445: Chiolero A, Gervasoni JP, Rwebogora A, Balampama M, Paccaud F, Bovet P.

Difference in blood pressure readings with mercury and automated devices: Impact

on hypertension prevalence estimates in Dar es Salaam, Tanzania. Eur J

Epidemiol. 2006;21(6):427-33. doi: 10.1007/s10654-006-9015-z. Epub 2006 Jul 7.

PMID: 16826452.

446: Otedo AE, Oyoo GO, Obondi JO, Otieno CF. Vasculitis in HIV: report of eight

cases. East Afr Med J. 2005 Dec;82(12):656-9. doi: 10.4314/eamj.v82i12.9373.

PMID: 16619712.

447: Lin C, Vakani R, Kussin P, Guhwe M, Farjat AE, Choudhury K, Renner D, Oduor

C, Graffagnino C; Neuroscience Knowledge Base Investigator Group. Assessment of

healthcare personnel knowledge of stroke care at a large referral hospital in

sub-Saharan Africa - A survey based approach. J Clin Neurosci. 2017

Aug;42:71-74. doi: 10.1016/j.jocn.2017.04.013. Epub 2017 Apr 28. PMID: 28457860.

448: Abbas ZG, Lutale J, Archibald LK. Rodent bites on the feet of diabetes

patients in Tanzania. Diabet Med. 2005 May;22(5):631-3. doi:

10.1111/j.1464-5491.2005.01488.x. PMID: 15842520.

449: Järhult B. Det diabola syndromet [The diabolic syndrome]. Lakartidningen.

2004 Sep 2;101(36):2746-7. Swedish. PMID: 15455640.

450: Cleland CR, Burton MJ, Hall C, Hall A, Courtright P, Makupa WU, Philippin

H. Diabetic retinopathy screening: experiences from northern Tanzania. Lancet

Diabetes Endocrinol. 2016 Jan;4(1):10-2. doi: 10.1016/S2213-8587(15)00422-2.

Epub 2015 Dec 3. PMID: 26656290.

451: PrayGod G, Changalucha J, Kapiga S, Todd J, Filteau S, Peck R. Elevated

blood pressure and correlates in a cohort of HIV-infected adults who started

antiretroviral therapy when undernourished. J Clin Hypertens (Greenwich). 2017

Aug;19(8):803-806. doi: 10.1111/jch.13031. Epub 2017 May 26. PMID: 28548437;

PMCID: PMC5568948.

452: Mwendwa FM, Otieno CF, Kayima JK, Amayo EO, Otieno PO. Risk factor profile

and the occurrence of microvascular complications in short-term type 2 diabetes

mellitus at Kenyatta National Hospital, Nairobi. East Afr Med J. 2005 Dec;82(12

Suppl):S163-72. doi: 10.4314/eamj.v82i12.9377. PMID: 16619728.

453: Alaro D, Bashir A, Musoke R, Wanaiana L. Prevalence and outcomes of acute

kidney injury in term neonates with perinatal asphyxia. Afr Health Sci. 2014

Sep;14(3):682-8. doi: 10.4314/ahs.v14i3.26. PMID: 25352889; PMCID: PMC4209658.

454: Murphy GA, Asiki G, Young EH, Seeley J, Nsubuga RN, Sandhu MS, Kamali A.

Cardiometabolic risk in a rural Ugandan population. Diabetes Care. 2013

Sep;36(9):e143. doi: 10.2337/dc13-0739. Erratum in: Diabetes Care. 2014

Apr;37(4):1169. Erratum in: Diabetes Care. 2017 Apr;40(4):625. PMID: 23970722;

PMCID: PMC3747938.

455: Kitua M, Davis M, Colvett J, Mwachiro M, Qayyum R. Clinical presentation,

management and outcome of cardiopulmonary resuscitation at Tenwek hospital: The

oCPRTen Study. Resuscitation. 2016 Sep;106:e15-6. doi:

10.1016/j.resuscitation.2016.06.008. Epub 2016 Jun 18. PMID: 27327228.

456: Connor DH, Somers K, Nelson AM, D'Arbela PG, Lukande R. The cause of

endomyocardial fibrosis in Uganda. Trop Doct. 2012 Oct;42(4):206-7. doi:

10.1258/td.2012.120245. Epub 2012 Aug 7. PMID: 22875807.

457: Shiroya-Wandabwa M, Yuko-Jowi C, Nduati R, Githanga J, Wamalwa D. Risk

factors for cardiac dysfunction in children on treatment for cancer at Kenyatta

National Hospital, Nairobi. East Afr Med J. 2009 Dec;86(12 Suppl):S52-7. doi:

10.4314/eamj.v86i12.62902. PMID: 21591510.

458: Swain JD, Mucumbisti J, Rusingiza E, Bolman RM 3rd, Binagwaho A. Cardiac

surgery for advanced rheumatic heart disease in Rwanda. Lancet Glob Health. 2014

Mar;2(3):e141-2. doi: 10.1016/S2214-109X(14)70022-1. Epub 2014 Feb 27. PMID:

25102844.

459: Tucker R. Advising pregnant women on miminising travel risks. Nurs Times.

2014 Apr 2-8;110(14):19-21. PMID: 24772798.

460: Lang'o MO, Githanga JN, Yuko-Jowi CA. Prevalence of iron deficiency in

children with cyanotic heart disease seen at Kenyatta National Hospital and

Mater Hospital Nairobi. East Afr Med J. 2009 Dec;86(12 Suppl):S47-51. doi:

10.4314/eamj.v86i12.62901. PMID: 21591509.

461: Hertz JT, Sakita FM, Kweka GL, Limkakeng AT, Galson SW, Ye JJ, Tarimo TG,

Temu G, Thielman NM, Bettger JP, Bartlett JA, Mmbaga BT, Bloomfield GS. Acute

myocardial infarction under-diagnosis and mortality in a Tanzanian emergency

department: A prospective observational study. Am Heart J. 2020 Aug;226:214-221.

doi: 10.1016/j.ahj.2020.05.017. Epub 2020 Jun 5. PMID: 32619815; PMCID:

PMC7442678.

462: Mohsen AH, Green ST, West JN, McKendrick MW. Myocarditis associated with

Plasmodium falciparum malaria: a case report and a review of the literature. J

Travel Med. 2001 Jul-Aug;8(4):219-20. doi: 10.2310/7060.2001.22133. PMID:

11712506.

463: Otieno CF. Diabetes care in Kenya--where are we and which way forward? East

Afr Med J. 2005 Dec;82(12 Suppl):S161-2. doi: 10.4314/eamj.v82i12.9376. PMID:

16619727.

464: di Summa M, Iezzi F, Oburu G, Mehta N. Huge left ventricular pseudoaneurysm

rupture in an African young adult patient. Interact Cardiovasc Thorac Surg. 2014

Jul;19(1):160-1. doi: 10.1093/icvts/ivu086. Epub 2014 Apr 11. PMID: 24729311.

465: Jafar TH. Commentary: cardiovascular risk factors--the next epidemic in

Uganda: findings from the population-based HIV/AIDS rural surveillance cohort.

Int J Epidemiol. 2011 Feb;40(1):171-3. doi: 10.1093/ije/dyq169. Epub 2010 Oct

29. PMID: 21037249.

466: Githaiga JW, Adwok JA. Wandering spleen presenting as a right hypochondrial

mass and intestinal obstruction. East Afr Med J. 2002 Aug;79(8):450-2. doi:

10.4314/eamj.v79i8.8834. PMID: 12638849.

467: Saidi HS, Olumbe AO, Kalebi A. Anatomy and pathology of coronary artery in

adult black Kenyans. East Afr Med J. 2002 Jun;79(6):323-7. doi:

10.4314/eamj.v79i6.8853. PMID: 12638823.

468: Cox SE, Soka D, Kirkham FJ, Newton CR, Prentice AM, Makani J, Younoszai AK.

Tricuspid regurgitant jet velocity and hospitalization in Tanzanian children

with sickle cell anemia. Haematologica. 2014 Jan;99(1):e1-4. doi:

10.3324/haematol.2013.089235. PMID: 24425690; PMCID: PMC4007918.

469: Ogendo SW. Long term valve-related morbidity following open-heart surgery

at the Kenyatta National Hospital. East Afr Med J. 2000 Apr;77(4):199-202. PMID:

12858903.

470: Grimaldi A, Ammirati E, Mirabel M, Marijon E. Challenges of using

ultrasounds for subclinical rheumatic heart disease screening. Int J Cardiol.

2013 Sep 10;167(6):3061. doi: 10.1016/j.ijcard.2012.11.083. Epub 2012 Dec 11.

PMID: 23245797.

471: Osowicki J, Steer AC. Diagnosis of rheumatic fever: the need for a better

test. Arch Dis Child. 2020 Sep;105(9):813-814. doi:

10.1136/archdischild-2020-318970. Epub 2020 Jun 29. PMID: 32601088.

472: Maro E, Janabi M. Echocardiographic profile of endomyocardial fibrosis in

Tanzania, East Africa. Cent Afr J Med. 2004 Sep-Oct;50(9-10):91-4. PMID:

16411343.

473: Urassa DP, Nystrom L, Carlstedt A, Msamanga GI, Lindmark G. Management of

hypertension in pregnancy as a quality indicator of antenatal care in rural

Tanzania. Afr J Reprod Health. 2003 Dec;7(3):69-76. PMID: 15055149.

474: Okello S, Rogers O, Byamugisha A, Rwebembera J, Buda AJ. Characteristics of

acute heart failure hospitalizations in a general medical ward in Southwestern

Uganda. Int J Cardiol. 2014 Oct 20;176(3):1233-4. doi:

10.1016/j.ijcard.2014.07.212. Epub 2014 Aug 4. PMID: 25127332; PMCID:

PMC4536291.

475: Freers J, Masembe V, Schmauz R, Mayanja-Kizza H. Endomyocardial fibrosis

syndrome in Uganda. Lancet. 2000 Jun 3;355(9219):1994-5. doi:

10.1016/S0140-6736(05)72932-9. PMID: 10859061.

476: Longenecker CT, Mondo C, Le VV, Jensen TP, Foster E. HIV infection is not

associated with echocardiographic signs of cardiomyopathy or pulmonary

hypertension among pregnant Ugandan women. Int J Cardiol. 2011 Mar

3;147(2):300-2. doi: 10.1016/j.ijcard.2010.12.047. Epub 2010 Dec 28. PMID:

21190741; PMCID: PMC3073580.

477: Longenecker CT, Okello E, Lwabi P, Costa MA, Simon DI, Salata RA.

Management of rheumatic heart disease in uganda: the emerging epidemic of non-

AIDS comorbidity in resource-limited settings. J Acquir Immune Defic Syndr. 2014

Feb 1;65(2):e79-80. doi: 10.1097/QAI.0b013e3182a03eb9. PMID: 24442227; PMCID:

PMC4423536.

478: Biasibetti E, Sferra C, Lynen G, Di Giulio G, De Meneghi D, Tomassone L,

Valenza F, Capucchio MT. Severe meningeal fibrinoid vasculitis associated with

Theileria taurotragi infection in two short-horned Zebu cattle. Trop Anim Health

Prod. 2016 Aug;48(6):1297-9. doi: 10.1007/s11250-016-1072-z. Epub 2016 May 4.

PMID: 27145963.

479: Beaman-Mbaya V, Ogola EN. The urinary levels of catecholamines, aldosterone

and cortisol in hypertensive East Africans: a pilot study. Ethn Dis. 2000

Autumn;10(3):357-63. PMID: 11110352.

480: Kaddumukasa MN, Goldstein LB. Author response: Influence of sodium

consumption and associated knowledge on poststroke hypertension in Uganda.

Neurology. 2017 Mar 14;88(11):1103. doi: 10.1212/WNL.0000000000003729. PMID:

28289171.

481: Koech MM, Barasa F, Ng'eno TK. Why prevention of rheumatic heart disease

should be a component of primary healthcare. J Trop Pediatr. 2012

Oct;58(5):414-5. doi: 10.1093/tropej/fmr101. Epub 2012 Jan 6. PMID: 22228821.

482: Ivy A, Tam J, Dewhurst MJ, Gray WK, Chaote P, Rogathi J, Dewhurst F, Walker

RW. Ambulatory blood pressure monitoring to assess the white-coat effect in an

elderly East African population. J Clin Hypertens (Greenwich). 2015

May;17(5):389-94. doi: 10.1111/jch.12501. Epub 2015 Feb 18. PMID: 25690267.

483: Mulei CM, Gathumbi PK, Mbuthia PG. Suspected sheep-associated malignant

catarrhal fever in a zero-grazed dairy herd in Kenya. Onderstepoort J Vet Res.

2000 Mar;67(1):43-7. PMID: 10843321.

484: Morbach S, Gröblinghoff U, Schulze H, Wulff S, Schönauer M, Rümenapf G,

Lutale JK, Abbas ZG. All-cause mortality after diabetes-related amputation in

Barbados: a prospective case-control study: response to Hambleton et Al.

Diabetes Care. 2009 Aug;32(8):e100; author reply e101. doi: 10.2337/dc09-0736.

PMID: 19638516.

485: Arioli F, D'Arbela PG, Aloi F, Nsubuga MM, Grimaldi A, Ammirati E.

Prosthetic thrombosis and pregnancy on warfarin: debate on mechanical mitral

valve replacement in sub-Saharan Africa based on a case report. Int J Cardiol.

2014 Oct 20;176(3):e86-8. doi: 10.1016/j.ijcard.2014.07.162. Epub 2014 Aug 4.

PMID: 25147063.

486: Campbell SD. Fahad's journey. Bull Am Coll Surg. 2008 Nov;93(11):8-10.

PMID: 19472827.

487: Jusabani A, Gray WK, Swai M, Walker R. Post-stroke carotid ultrasound

findings from an incident Tanzanian population. Neuroepidemiology.

2011;37(3-4):245-8. doi: 10.1159/000334610. Epub 2011 Dec 7. PMID: 22156625.

488: Yung BM, Browne-Yung K, Marsh K. Outcome of cardiopulmonary resuscitation

in hospitalized African children. J Trop Pediatr. 2001 Apr;47(2):108-10. PMID:

11336126.

489: Fielder JF. A 23-year-old woman admitted to Kijabe Mission Hospital with

bilateral lower extremity gangrene. MedGenMed. 2004 Mar 15;6(1):56. PMID:

15208567; PMCID: PMC1140744.

490: Opio MO, Kellett J; Kitovu Hospital Study Group. The association between a

simple measure of QRS voltage and the in-hospital mortality of acutely ill

medical patients. Eur J Intern Med. 2017 Apr;39:e9. doi:

10.1016/j.ejim.2017.03.001. Epub 2017 Mar 6. PMID: 28279566.

491: Yonga GO, Munene JC, Ogendo SW. Post-infarction ventricular septal defect

in Nairobi: case report. East Afr Med J. 2005 Dec;82(12):660-2. doi:

10.4314/eamj.v82i12.9374. PMID: 16619713.

492: Shavadia J, Shah R, Yonga G, Patel R, Stebbing J, Nelson M. The influence

of antiretroviral therapy on the QTc interval in an African cohort. Clin Infect

Dis. 2012 Feb 1;54(3):448-9. doi: 10.1093/cid/cir712. Epub 2011 Dec 9. PMID:

22157175.

493: Juttner K, McKenzie L, Razzak N, Zehyle E, Belliere M, Anderson D, Tulloh

R. Cardiac hydatid disease in the third world. BMJ Case Rep. 2011 Apr

1;2011:bcr1220103609. doi: 10.1136/bcr.12.2010.3609. PMID: 22700616; PMCID:

PMC3079493.

494: Tufton N, Patel RR. Prevalence of hypertensive disorders in a prenatal

clinic in Zanzibar. Int J Gynaecol Obstet. 2011 Jan;112(1):69-70. doi:

10.1016/j.ijgo.2010.09.005. Epub 2010 Nov 16. PMID: 21084085.

495: Truelsen T. Stroke incidence studies in Africa. Lancet Neurol. 2010

Aug;9(8):755-7. doi: 10.1016/S1474-4422(10)70161-7. Epub 2010 Jul 6. PMID:

20609628.

496: Shah R. From the heart. Interview by Lynne Wallis. Nurs Stand. 2003 Oct

15-21;18(5):16. PMID: 14603761.

497: Galukande M, Fualal J, Nyangoma E. Mesenteric venous thrombosis in Uganda:

a retrospective study of five cases. Afr Health Sci. 2009 Dec;9(4):284-9. PMID:

21503183; PMCID: PMC3074391.

498: Joshi MD, Amayo EO. Cardiovascular risk factors in patients with type 2

diabetes mellitus in Kenya: levels of control attained at the Outpatient

Diabetic Clinic of Kenyatta National Hospital, Nairobi. East Afr Med J. 2006

Jul;83(7):405-6; author reply 406-7. PMID: 17089502.

499: Wiggin TR. Emergency craniotomy using improvised instruments. Trop Doct.

2001 Jul;31(3):174. doi: 10.1177/004947550103100322. PMID: 11444346.

500: Isaksson G. Tuberkulös perikardit med hjärttamponad--inte ovanligt i Kenya.

Perikardiocentes kan vara livräddande [Tuberculous pericarditis with cardiac

tamponade--not uncommon in Kenya. Pericardiocentesis can be life-saving].

Lakartidningen. 2004 Mar 18;101(12):1099-100. Swedish. PMID: 15088358.

501: Pastakia SD, Vincent WR 3rd, Manji I, Kamau E, Schellhase EM. Clinical

pharmacy consultations provided by American and Kenyan pharmacy students during

an acute care advanced pharmacy practice experience. Am J Pharm Educ. 2011 Apr

11;75(3):42. doi: 10.5688/ajpe75342. PMID: 21655396; PMCID: PMC3109796.

502: Howitt SC, Jones MP, Jusabani A, Gray WK, Aris E, Mugusi F, Swai M, Walker

RW. A cross-sectional study of quality of life in incident stroke survivors in

rural northern Tanzania. J Neurol. 2011 Aug;258(8):1422-30. doi:

10.1007/s00415-011-5948-6. Epub 2011 Feb 19. PMID: 21336782.

503: Katende D, Mutungi G, Baisley K, Biraro S, Ikoona E, Peck R, Smeeth L,

Hayes R, Munderi P, Grosskurth H. Readiness of Ugandan health services for the

management of outpatients with chronic diseases. Trop Med Int Health. 2015

Oct;20(10):1385-95. doi: 10.1111/tmi.12560. Epub 2015 Jul 8. PMID: 26095069;

PMCID: PMC4758403.

504: Mutimura E, Crowther NJ, Cade TW, Yarasheski KE, Stewart A. Exercise

training reduces central adiposity and improves metabolic indices in HAART-

treated HIV-positive subjects in Rwanda: a randomized controlled trial. AIDS Res

Hum Retroviruses. 2008 Jan;24(1):15-23. doi: 10.1089/aid.2007.0023. PMID:

18275343; PMCID: PMC3936606.

505: Wandabwa J, Doyle P, Todd J, Ononge S, Kiondo P. Risk factors for severe

post partum haemorrhage in Mulago hospital, Kampala, Uganda. East Afr Med J.

2008 Feb;85(2):64-71. doi: 10.4314/eamj.v85i2.9608. PMID: 18557249.

506: de Mast Q, Molhoek JE, van der Ven AJ, Gray WK, de Groot PG, Jusabani A,

Mugusi F, Urbanus RT, Walker RW. Antiphospholipid Antibodies and the Risk of

Stroke in Urban and Rural Tanzania: A Community-Based Case-Control Study.

Stroke. 2016 Oct;47(10):2589-95. doi: 10.1161/STROKEAHA.116.013760. Epub 2016

Sep 13. PMID: 27625376.

507: Makubi A, Hage C, Lwakatare J, Mmbando B, Kisenge P, Lund LH, Rydén L,

Makani J. Prevalence and prognostic implications of anaemia and iron deficiency

in Tanzanian patients with heart failure. Heart. 2015 Apr;101(8):592-9. doi:

10.1136/heartjnl-2014-306890. Epub 2014 Dec 24. PMID: 25539945; PMCID:

PMC5573172.

508: Akintunde A, Nondi J, Gogo K, Jones ESW, Rayner BL, Hackam DG, Spence JD.

Physiological Phenotyping for Personalized Therapy of Uncontrolled Hypertension

in Africa. Am J Hypertens. 2017 Sep 1;30(9):923-930. doi: 10.1093/ajh/hpx066.

PMID: 28472315.

509: Terio KA, Kinsel MJ, Raphael J, Mlengeya T, Lipende I, Kirchhoff CA,

Gilagiza B, Wilson ML, Kamenya S, Estes JD, Keele BF, Rudicell RS, Liu W, Patton

S, Collins A, Hahn BH, Travis DA, Lonsdorf EV. Pathologic lesions in chimpanzees

(Pan trogylodytes schweinfurthii) from Gombe National Park, Tanzania, 2004-2010.

J Zoo Wildl Med. 2011 Dec;42(4):597-607. doi: 10.1638/2010-0237.1. PMID:

22204054; PMCID: PMC3693847.

510: Warshow MM. Obesity: what lies beneath? East Afr Med J. 2002 Feb;79(2):57.

doi: 10.4314/eamj.v79i2.8900. PMID: 12380876.

511: Majaliwa ES, Munubhi E, Ramaiya K, Mpembeni R, Sanyiwa A, Mohn A, Chiarelli

F. Survey on acute and chronic complications in children and adolescents with

type 1 diabetes at Muhimbili National Hospital in Dar es Salaam, Tanzania.

Diabetes Care. 2007 Sep;30(9):2187-92. doi: 10.2337/dc07-0594. Epub 2007 Jun 11.

PMID: 17563337.

512: Lumsden RH, Akwanalo C, Chepkwony S, Kithei A, Omollo V, Holland TL,

Bloomfield GS, O'Meara WP. Clinical and geographic patterns of rheumatic heart

disease in outpatients attending cardiology clinic in western Kenya. Int J

Cardiol. 2016 Nov 15;223:228-235. doi: 10.1016/j.ijcard.2016.08.069. Epub 2016

Aug 4. PMID: 27541662.

513: Brent B, Obonyo N, Akech S, Shebbe M, Mpoya A, Mturi N, Berkley JA, Tulloh

RMR, Maitland K. Assessment of Myocardial Function in Kenyan Children With

Severe, Acute Malnutrition: The Cardiac Physiology in Malnutrition (CAPMAL)

Study. JAMA Netw Open. 2019 Mar 1;2(3):e191054. doi:

10.1001/jamanetworkopen.2019.1054. PMID: 30901050; PMCID: PMC6583281.

514: Ndayambagye EB, Nakalembe M, Kaye DK. Factors associated with persistent

hypertension after puerperium among women with pre-eclampsia/eclampsia in Mulago

hospital, Uganda. BMC Pregnancy Childbirth. 2010 Mar 12;10:12. doi:

10.1186/1471-2393-10-12. PMID: 20222993; PMCID: PMC2848130.

515: Pallangyo P, Nicholaus P, Kisenge P, Mayala H, Swai N, Janabi M. A

community-based study on prevalence and correlates of erectile dysfunction among

Kinondoni District Residents, Dar Es Salaam, Tanzania. Reprod Health. 2016 Nov

29;13(1):140. doi: 10.1186/s12978-016-0249-2. PMID: 27899129; PMCID: PMC5129661.

516: Okello S, Millard A, Owori R, Asiimwe SB, Siedner MJ, Rwebembera J, Wilson

LA, Moore CC, Annex BH. Prevalence of lower extremity peripheral artery disease

among adult diabetes patients in southwestern Uganda. BMC Cardiovasc Disord.

2014 Jun 10;14:75. doi: 10.1186/1471-2261-14-75. PMID: 24913468; PMCID:

PMC4057935.

517: Idro R, Karamagi C, Tumwine J. Immediate outcome and prognostic factors for

cerebral malaria among children admitted to Mulago Hospital, Uganda. Ann Trop

Paediatr. 2004 Mar;24(1):17-24. doi: 10.1179/027249304225013240. PMID: 15005962.

518: Kaye D. Risk factors for preterm premature rupture of membranes at Mulago

Hospital, Kampala. East Afr Med J. 2001 Feb;78(2):65-9. PMID: 11682948.

519: Nyanzi R, Wamala R, Atuhaire LK. Diabetes and quality of life: a Ugandan

perspective. J Diabetes Res. 2014;2014:402012. doi: 10.1155/2014/402012. Epub

2014 Mar 2. PMID: 24724107; PMCID: PMC3958803.

520: Nielsen J, Bahendeka SK, Gregg EW, Whyte SR, Bygbjerg IC, Meyrowitsch DW. A

comparison of cardiometabolic risk factors in households in rural Uganda with

and without a resident with type 2 diabetes, 2012-2013. Prev Chronic Dis. 2015

Apr 2;12:E44. doi: 10.5888/pcd12.140486. PMID: 25837257; PMCID: PMC4383445.

521: Saadi A, Okeng'o K, Biseko MR, Shayo AF, Mmbando TN, Grundy SJ, Xu A,

Parker RA, Wibecan L, Iyer G, Onesmo PM, Kapina BN, Regenhardt RW, Mateen FJ.

Post-stroke social networks, depressive symptoms, and disability in Tanzania: A

prospective study. Int J Stroke. 2018 Oct;13(8):840-848. doi:

10.1177/1747493018772788. Epub 2018 Apr 20. PMID: 29676225; PMCID: PMC6919309.

522: Arioli F, Ammirati E. Mitral prosthetic valve thrombosis and cardiogenic

shock in a limited resource setting in sub-Saharan Africa: a tailored treatment

approach. Int J Cardiol. 2015 Jan 15;178:65-6. doi:

10.1016/j.ijcard.2014.10.105. Epub 2014 Oct 22. PMID: 25464220.

523: Mwangi N, Ng'ang'a M, Gakuo E, Gichuhi S, Macleod D, Moorman C, Muthami L,

Tum P, Jalango A, Githeko K, Gichangi M, Kibachio J, Bascaran C, Foster A.

Effectiveness of peer support to increase uptake of retinal examination for

diabetic retinopathy: study protocol for the DURE pragmatic cluster randomized

clinical trial in Kirinyaga, Kenya. BMC Public Health. 2018 Jul 13;18(1):871.

doi: 10.1186/s12889-018-5761-6. PMID: 30005643; PMCID: PMC6044026.

524: Brown CJ, Chenery SR, Smith B, Mason C, Tomkins A, Roberts GJ, Sserunjogi

L, Tiberindwa JV. Environmental influences on the trace element content of teeth

--implications for disease and nutritional status. Arch Oral Biol. 2004

Sep;49(9):705-17. doi: 10.1016/j.archoralbio.2004.04.008. PMID: 15275858.

525: Christensen DL, Faurholt-Jepsen D, Birkegaard L, Mwaniki DL, Boit MK,

Kilonzo B, Brage S, Friis H, Tetens I, Borch-Johnsen K, Vistisen D.

Cardiovascular risk factors in rural Kenyans are associated with differential

age gradients, but not modified by sex or ethnicity. Ann Hum Biol.

2016;43(1):42-9. doi: 10.3109/03014460.2015.1013987. Epub 2015 Jun 15. PMID:

26073640.

526: Shavadia J, Yonga G, Otieno H. A prospective review of acute coronary

syndromes in an urban hospital in sub-Saharan Africa. Cardiovasc J Afr. 2012

Jul;23(6):318-21. doi: 10.5830/CVJA-2012-002. PMID: 22836154; PMCID: PMC3734739.

527: Hagmann CF, Robertson NJ, Acolet D, Chan D, Onda S, Nyombi N, Nakakeeto M,

Cowan FM. Cranial ultrasound findings in well newborn Ugandan infants. Arch Dis

Child Fetal Neonatal Ed. 2010 Sep;95(5):F338-44. doi: 10.1136/adc.2009.174607.

Epub 2010 May 20. PMID: 20488861.

528: Kidy F, Rutebarika D, Lule SA, Kizza M, Odiit A, Webb EL, Elliott AM. Blood

pressure in primary school children in Uganda: a cross-sectional survey. BMC

Public Health. 2014 Nov 26;14:1223. doi: 10.1186/1471-2458-14-1223. PMID:

25427456; PMCID: PMC4289384.

529: Kim JK, Chang YS, Sung S, Park WS. Mortality rate-dependent variations in

the survival without major morbidities rate of extremely preterm infants. Sci

Rep. 2019 May 14;9(1):7371. doi: 10.1038/s41598-019-43879-z. PMID: 31089251;

PMCID: PMC6517394.

530: Muyanja D, Muzoora C, Muyingo A, Muyindike W, Siedner MJ. High Prevalence

of Metabolic Syndrome and Cardiovascular Disease Risk Among People with HIV on

Stable ART in Southwestern Uganda. AIDS Patient Care STDS. 2016 Jan;30(1):4-10.

doi: 10.1089/apc.2015.0213. Epub 2015 Dec 18. PMID: 26683587; PMCID: PMC4717524.

531: Kimani KN, Murray SA, Grant L. Multidimensional needs of patients living

and dying with heart failure in Kenya: a serial interview study. BMC Palliat

Care. 2018 Feb 17;17(1):28. doi: 10.1186/s12904-018-0284-6. PMID: 29454383;

PMCID: PMC5816535.

532: Rockers PC, Wirtz VJ, Vian T, Onyango MA, Ashigbie PG, Laing R. Study

protocol for a cluster-randomised controlled trial of an NCD access to medicines

initiative: evaluation of Novartis Access in Kenya. BMJ Open. 2016 Nov

25;6(11):e013386. doi: 10.1136/bmjopen-2016-013386. PMID: 27888177; PMCID:

PMC5168521.

533: Otieno CF, Vaghela V, Mwendwa FW, Kayima JK, Ogola EN. Cardiovascular risk

factors in patients with type 2 diabetes mellitus in Kenya: levels of control

attained at the Outpatient Diabetic Clinic of Kenyatta National Hospital,

Nairobi. East Afr Med J. 2005 Dec;82(12 Suppl):S184-90. doi:

10.4314/eamj.v82i12.9380. PMID: 16619731.

534: Maganga E, Smart LR, Kalluvya S, Kataraihya JB, Saleh AM, Obeid L, Downs

JA, Fitzgerald DW, Peck RN. Glucose Metabolism Disorders, HIV and Antiretroviral

Therapy among Tanzanian Adults. PLoS One. 2015 Aug 19;10(8):e0134410. doi:

10.1371/journal.pone.0134410. PMID: 26287742; PMCID: PMC4545793.

535: Cohen D. Stroke thrombolysis in Mombasa--an outreach service. Pract Neurol.

2011 Dec;11(6):372-4. doi: 10.1136/practneurol-2011-000038. PMID: 22100952.

536: Nguku SW, Wanyoike-Gichuhi J, Aywak AA. Biophysical profile scores and

resistance indices of the umbilical artery as seen in patients with pregnancy

induced hypertension. East Afr Med J. 2006 Mar;83(3):96-101. doi:

10.4314/eamj.v83i3.9404. PMID: 16771106.

537: Lewis BC, Nair PC, Heran SS, Somogyi AA, Bowden JJ, Doogue MP, Miners JO.

Warfarin resistance associated with genetic polymorphism of VKORC1: linking

clinical response to molecular mechanism using computational modeling.

Pharmacogenet Genomics. 2016 Jan;26(1):44-50. doi: 10.1097/FPC.0000000000000184.

PMID: 26513304.

538: Mutua EM, Gitonga MM, Mbuthia B, Muiruri N, Cheptum JJ, Maingi T. Level of

blood pressure control among hypertensive patients on follow-up in a regional

referral hospital in Central Kenya. Pan Afr Med J. 2014 Aug 5;18:278. doi:

10.11604/pamj.2014.18.278.4308. PMID: 25489372; PMCID: PMC4258197.

539: Kamara NT, Asiimwe S. Dyslipidaemia and hypertension among adults with

diabetes in rural Uganda. Trop Doct. 2010 Jan;40(1):41-2. doi:

10.1258/td.2009.090086. PMID: 20075425.

540: Vannappagari V, Albano JD, Koram N, Tilson H, Scheuerle AE, Napier MD.

Prenatal exposure to zidovudine and risk for ventricular septal defects and

congenital heart defects: data from the Antiretroviral Pregnancy Registry. Eur J

Obstet Gynecol Reprod Biol. 2016 Feb;197:6-10. doi:

10.1016/j.ejogrb.2015.11.015. Epub 2015 Nov 24. PMID: 26687320.

541: Kalyesubula R, Nankabirwa JI, Ssinabulya I, Siddharthan T, Kayima J,

Nakibuuka J, Salata RA, Mondo C, Kamya MR, Hricik D. Kidney disease in Uganda: a

community based study. BMC Nephrol. 2017 Apr 3;18(1):116. doi:

10.1186/s12882-017-0521-x. PMID: 28372551; PMCID: PMC5379733.

542: Ukah UV, Payne B, Lee T, Magee LA, von Dadelszen P; fullPIERS and miniPIERS

Working Groups. External Validation of the fullPIERS Model for Predicting

Adverse Maternal Outcomes in Pregnancy Hypertension in Low- and Middle-Income

Countries. Hypertension. 2017 Apr;69(4):705-711. doi:

10.1161/HYPERTENSIONAHA.116.08706. Epub 2017 Feb 6. PMID: 28167685.

543: Lu JC, Sable C, Ensing GJ, Webb C, Scheel J, Aliku T, Lwabi P, Godown J,

Beaton A. Simplified rheumatic heart disease screening criteria for handheld

echocardiography. J Am Soc Echocardiogr. 2015 Apr;28(4):463-9. doi:

10.1016/j.echo.2015.01.001. Epub 2015 Feb 7. PMID: 25660669.

544: Aywak AA, Masesa JV. Comparison of sonography with venography in the

diagnosis of deep venous thrombosis. East Afr Med J. 2007 Jul;84(7):304-11. doi:

10.4314/eamj.v84i7.9584. PMID: 17886423.

545: Nanji K, Kherani IN, Damji KF, Nyenze M, Kiage D, Tennant MT. The Muranga

Teleophthalmology Study: A Comparison of Virtual (Teleretina) Assessment with

in-person Clinical Examination to Diagnose Diabetic Retinopathy and Age-related

Macular Degeneration in Kenya. Middle East Afr J Ophthalmol. 2020 Jul

20;27(2):91-99. doi: 10.4103/meajo.MEAJO_144_19. PMID: 32874041; PMCID:

PMC7442076.

546: Haregu TN, Oti S, Egondi T, Kyobutungi C. Co-occurrence of behavioral risk

factors of common non-communicable diseases among urban slum dwellers in

Nairobi, Kenya. Glob Health Action. 2015 Sep 16;8:28697. doi:

10.3402/gha.v8.28697. PMID: 26385542; PMCID: PMC4575413.

547: Lubega S, Aliku T, Lwabi P. Echocardiographic pattern and severity of valve

dysfunction in children with rheumatic heart disease seen at Uganda Heart

Institute, Mulago hospital. Afr Health Sci. 2014 Sep;14(3):617-25. doi:

10.4314/ahs.v14i3.17. PMID: 25352880; PMCID: PMC4209653.

548: Pastakia SD, Ali SM, Kamano JH, Akwanalo CO, Ndege SK, Buckwalter VL,

Vedanthan R, Bloomfield GS. Screening for diabetes and hypertension in a rural

low income setting in western Kenya utilizing home-based and community-based

strategies. Global Health. 2013 May 16;9:21. doi: 10.1186/1744-8603-9-21. PMID:

23680083; PMCID: PMC3662603.

549: Tarus NK, Pau AK, Sereti I, Kirui FK, Sawe FK, Agan BK, Momanyi LM, Ngeno

HC, Koskei GK, Shaffer DN. CHALLENGES IN MANAGEMENT OF WARFARIN ANTI-COAGULATION

IN ADVANCED HIV/AIDS PATIENTS WITH VENOUS THROMBOTIC EVENTS--A CASE SERIES FROM

A RESEARCH CLINIC IN RURAL KERICHO, KENYA. East Afr Med J. 2013

Jul;90(7):207-13. PMID: 26862618.

550: Manji I, Pastakia SD, DO AN, Ouma MN, Schellhase E, Karwa R, Miller ML,

Saina C, Akwanalo C. Performance outcomes of a pharmacist-managed

anticoagulation clinic in the rural, resource-constrained setting of Eldoret,

Kenya. J Thromb Haemost. 2011 Nov;9(11):2215-20. doi:

10.1111/j.1538-7836.2011.04503.x. PMID: 21914124.

551: Nkumbe HE, Kollmann KH, Gaeckle HC. Assessment of diabetic retinopathy in

newly diagnosed black Kenyan type 2 diabetics. East Afr Med J. 2010

Mar;87(3):109-14. doi: 10.4314/eamj.v87i3.62196. PMID: 23057306.

552: Ndege BW, Diero LO, Owiti MO, Anjichi G, Siika AM. PREVALENCE, TREATMENT

AND CONTROL OF HYPERTENSION AMONG TYPE 2 DIABETIC PATIENTS AT MOI TEACHING AND

REFERRAL HOSPITAL, ELDORET, KENYA. East Afr Med J. 2014 Aug;91(8):253-60. PMID:

26862649.

553: Swain JD, Pugliese DN, Mucumbitsi J, Rusingiza EK, Ruhamya N, Kagame A,

Ganza G, Come PC, Breakey S, Greenwood B, Muehlschlegel JD, Patton-Bolman C,

Binagwaho A, Morton Bolman R. Partnership for sustainability in cardiac surgery

to address critical rheumatic heart disease in sub-Saharan Africa: the

experience from Rwanda. World J Surg. 2014 Sep;38(9):2205-11. doi:

10.1007/s00268-014-2559-2. PMID: 24728579.

554: Bloomfield GS, DeLong AK, Akwanalo CO, Hogan JW, Carter EJ, Aswa DF,

Binanay C, Koech M, Kimaiyo S, Velazquez EJ. Markers of Atherosclerosis,

Clinical Characteristics, and Treatment Patterns in Heart Failure: A Case-

Control Study of Middle-Aged Adult Heart Failure Patients in Rural Kenya. Glob

Heart. 2016 Mar;11(1):97-107. doi: 10.1016/j.gheart.2015.12.014. PMID: 27102027;

PMCID: PMC4843836.

555: Bradley-Hewitt T, Dantin A, Ploutz M, Aliku T, Lwabi P, Sable C, Beaton A.

The Impact of Echocardiographic Screening for Rheumatic Heart Disease on Patient

Quality of Life. J Pediatr. 2016 Aug;175:123-9. doi:

10.1016/j.jpeds.2016.04.087. Epub 2016 Jun 4. PMID: 27268786.

556: Mwakyusa SD, Manji KP, Massawe AW. The hypoxic ischaemic encephalopathy

score in predicting neurodevelopmental outcomes among infants with birth

asphyxia at the Muhimbili National Hospital, Dar-es-Salaam, Tanzania. J Trop

Pediatr. 2009 Feb;55(1):8-14. doi: 10.1093/tropej/fmn061. Epub 2008 Jul 11.

PMID: 18621775.

557: Karinja M, Pillai G, Schlienger R, Tanner M, Ogutu B. Care-Seeking Dynamics

among Patients with Diabetes Mellitus and Hypertension in Selected Rural

Settings in Kenya. Int J Environ Res Public Health. 2019 Jun 6;16(11):2016. doi:

10.3390/ijerph16112016. PMID: 31174248; PMCID: PMC6603942.

558: Stanifer JW, Cleland CR, Makuka GJ, Egger JR, Maro V, Maro H, Karia F,

Patel UD, Burton MJ, Philippin H. Prevalence, Risk Factors, and Complications of

Diabetes in the Kilimanjaro Region: A Population-Based Study from Tanzania. PLoS

One. 2016 Oct 6;11(10):e0164428. doi: 10.1371/journal.pone.0164428. PMID:

27711179; PMCID: PMC5053499.

559: Negishi H, Ikeda K, Kuga S, Noguchi T, Kanda T, Njelekela M, Liu L, Miki T,

Nara Y, Sato T, Mashalla Y, Mtabaji J, Yamori Y. The relation of oxidative DNA

damage to hypertension and other cardiovascular risk factors in Tanzania. J

Hypertens. 2001 Mar;19(3 Pt 2):529-33. doi: 10.1097/00004872-200103001-00002.

PMID: 11327625.

560: Macharia AW, Mochamah G, Uyoga S, Ndila CM, Nyutu G, Makale J, Tendwa M,

Nyatichi E, Ojal J, Shebe M, Awuondo KO, Mturi N, Peshu N, Tsofa B, Scott JAG,

Maitland K, Williams TN. The clinical epidemiology of sickle cell anemia In

Africa. Am J Hematol. 2018 Mar;93(3):363-370. doi: 10.1002/ajh.24986. Epub 2017

Dec 18. PMID: 29168218; PMCID: PMC6175377.

561: Marshall SL, Edidin D, Sharma V, Ogle G, Arena VC, Orchard T. Current

clinical status, glucose control, and complication rates of children and youth

with type 1 diabetes in Rwanda. Pediatr Diabetes. 2013 May;14(3):217-26. doi:

10.1111/pedi.12007. Epub 2012 Dec 28. PMID: 23279222.

562: Namukwaya E, Grant L, Downing J, Leng M, Murray SA. Improving care for

people with heart failure in Uganda: serial in-depth interviews with patients'

and their health care professionals. BMC Res Notes. 2017 May 25;10(1):184. doi:

10.1186/s13104-017-2505-0. PMID: 28545502; PMCID: PMC5445313.

563: Graham SM, Chen J, Chung DW, Barker KR, Conroy AL, Hawkes MT, Namasopo S,

Kain KC, López JA, Liles WC. Endothelial activation, haemostasis and thrombosis

biomarkers in Ugandan children with severe malaria participating in a clinical

trial. Malar J. 2016 Feb 2;15:56. doi: 10.1186/s12936-016-1106-z. PMID:

26830467; PMCID: PMC4736470.

564: Muddu M, Tusubira AK, Sharma SK, Akiteng AR, Ssinabulya I, Schwartz JI.

Integrated Hypertension and HIV Care Cascades in an HIV Treatment Program in

Eastern Uganda: A Retrospective Cohort Study. J Acquir Immune Defic Syndr. 2019

Aug 15;81(5):552-561. doi: 10.1097/QAI.0000000000002067. PMID: 31045649; PMCID:

PMC6625912.

565: Ngonzi J, Tornes YF, Mukasa PK, Salongo W, Kabakyenga J, Sezalio M, Wouters

K, Jacqueym Y, Van Geertruyden JP. Puerperal sepsis, the leading cause of

maternal deaths at a Tertiary University Teaching Hospital in Uganda. BMC

Pregnancy Childbirth. 2016 Aug 5;16(1):207. doi: 10.1186/s12884-016-0986-9.

PMID: 27495904; PMCID: PMC4974713.

566: Nonterah EA, Boua PR, Klipstein-Grobusch K, Asiki G, Micklesfield LK,

Agongo G, Ali SA, Mashinya F, Sorgho H, Nakanabo-Diallo S, Debpuur C, Kyobutungi

C, Alberts M, Norris S, Tollman S, Tinto H, Soo CC, Mukomana F, Hazelhurst S,

Wade AN, Kahn K, Oduro AR, Grobbee DE, Sankoh O, Ramsay M, Bots ML, Crowther NJ;

as members and collaborators of AWI‐Gen and the H3Africa Consortium. Classical

Cardiovascular Risk Factors and HIV are Associated With Carotid Intima-Media

Thickness in Adults From Sub-Saharan Africa: Findings From H3Africa AWI-Gen

Study. J Am Heart Assoc. 2019 Jul 16;8(14):e011506. doi:

10.1161/JAHA.118.011506. Epub 2019 Jul 13. PMID: 31304842; PMCID: PMC6662137.

567: Khabala KB, Edwards JK, Baruani B, Sirengo M, Musembi P, Kosgei RJ, Walter

K, Kibachio JM, Tondoi M, Ritter H, Wilkinson E, Reid T. Medication Adherence

Clubs: a potential solution to managing large numbers of stable patients with

multiple chronic diseases in informal settlements. Trop Med Int Health. 2015

Oct;20(10):1265-70. doi: 10.1111/tmi.12539. Epub 2015 Jun 15. PMID: 25962952;

PMCID: PMC4744994.

568: Asiki G, Murphy GA, Baisley K, Nsubuga RN, Karabarinde A, Newton R, Seeley

J, Young EH, Kamali A, Sandhu MS. Prevalence of dyslipidaemia and associated

risk factors in a rural population in South-Western Uganda: a community based

survey. PLoS One. 2015 May 14;10(5):e0126166. doi: 10.1371/journal.pone.0126166.

Erratum in: PLoS One. 2017 Feb 24;12 (2):e0173133. PMID: 25974077; PMCID:

PMC4431752.

569: Rockers PC, Laing RO, Ashigbie PG, Onyango MA, Mukiira CK, Wirtz VJ. Effect

of Novartis Access on availability and price of non-communicable disease

medicines in Kenya: a cluster-randomised controlled trial. Lancet Glob Health.

2019 Apr;7(4):e492-e502. doi: 10.1016/S2214-109X(18)30563-1. Epub 2019 Feb 21.

PMID: 30799142.

570: Otieno CF, Vaghela V, Ogola EN, Amayo EO. Patterns of homocysteine in

Kenyans with type 2 diabetes without overt cardiovascular disease at Kenyatta

National Hospital, Nairobi. East Afr Med J. 2005 Dec;82(12 Suppl):S180-3. doi:

10.4314/eamj.v82i12.9379. PMID: 16619730.

571: Rachlis B, Naanyu V, Wachira J, Genberg B, Koech B, Kamene R, Akinyi J,

Braitstein P. Identifying common barriers and facilitators to linkage and

retention in chronic disease care in western Kenya. BMC Public Health. 2016 Aug

8;16:741. doi: 10.1186/s12889-016-3462-6. Erratum in: BMC Public Health. 2018

Aug 10;18(1):1003. PMID: 27503191; PMCID: PMC4977618.

572: Robberstad B, Norheim OF. Incorporating concerns for equal lifetime health

in evaluations of public health programs. Soc Sci Med. 2011 May;72(10):1711-6.

doi: 10.1016/j.socscimed.2011.03.032. Epub 2011 Apr 13. PMID: 21531492.

573: Mumba M, Hall A, Lewallen S. Compliance with eye screening examinations

among diabetic patients at a Tanzanian referral hospital. Ophthalmic Epidemiol.

2007 Sep-Oct;14(5):306-10. doi: 10.1080/09286580701272079. PMID: 17994440.

574: Njelekela M, Ikeda K, Mtabaji J, Yamori Y. Dietary habits, plasma

polyunsaturated fatty acids and selected coronary disease risk factors in

Tanzania. East Afr Med J. 2005 Nov;82(11):572-8. doi: 10.4314/eamj.v82i11.9412.

PMID: 16463751.

575: Baker T, Schell CO, Lugazia E, Blixt J, Mulungu M, Castegren M, Eriksen J,

Konrad D. Vital Signs Directed Therapy: Improving Care in an Intensive Care Unit

in a Low-Income Country. PLoS One. 2015 Dec 22;10(12):e0144801. doi:

10.1371/journal.pone.0144801. PMID: 26693728; PMCID: PMC4687915.

576: Ince P, Abbas ZG, Lutale JK, Basit A, Ali SM, Chohan F, Morbach S,

Möllenberg J, Game FL, Jeffcoate WJ. Use of the SINBAD classification system and

score in comparing outcome of foot ulcer management on three continents.

Diabetes Care. 2008 May;31(5):964-7. doi: 10.2337/dc07-2367. Epub 2008 Feb 25.

PMID: 18299441.

577: Yuko-Jowi CA, Okello CA, Mutai L. COMPLEMENTARY TECHNIQUES OF PERCUTANEOUS

CLOSURE OF DUCTUS ARTERIOSUS USING DETACHABLE COOK COILS AND AMPLATZER DEVICES.

East Afr Med J. 2013 Jul;90(7):214-21. PMID: 26862619.

578: Waweru P, Gatimu SM. Mortality and functional outcomes after a spontaneous

subarachnoid haemorrhage: A retrospective multicentre cross-sectional study in

Kenya. PLoS One. 2019 Jun 12;14(6):e0217832. doi: 10.1371/journal.pone.0217832.

PMID: 31188844; PMCID: PMC6561561.

579: Temu TM, Kirui N, Wanjalla C, Ndungu AM, Kamano JH, Inui TS, Bloomfield GS.

Cardiovascular health knowledge and preventive practices in people living with

HIV in Kenya. BMC Infect Dis. 2015 Oct 14;15:421. doi:

10.1186/s12879-015-1157-8. PMID: 26466584; PMCID: PMC4607097.

580: Winnicki M, Puato M, Somers VK, Zambon A, Marcovina SM, Rattazzi M,

Phillips BG, Pauletto P. Leptin is associated with the size of the

apolipoprotein(a) particle in African tribal populations living on fish or

vegetarian diet. Atherosclerosis. 2010 Jul;211(1):303-7. doi:

10.1016/j.atherosclerosis.2010.01.041. Epub 2010 Feb 4. PMID: 20197188.

581: Etyang AO, Sigilai A, Odipo E, Oyando R, Ong'ayo G, Muthami L, Munge K,

Kirui F, Mbui J, Bukania Z, Mwai J, Obala A, Barasa E. Diagnostic Accuracy of

Unattended Automated Office Blood Pressure Measurement in Screening for

Hypertension in Kenya. Hypertension. 2019 Dec;74(6):1490-1498. doi:

10.1161/HYPERTENSIONAHA.119.13574. Epub 2019 Oct 7. PMID: 31587589; PMCID:

PMC7069390.

582: Wirtz VJ, Turpin K, Laing RO, Mukiira CK, Rockers PC. Access to medicines

for asthma, diabetes and hypertension in eight counties of Kenya. Trop Med Int

Health. 2018 Aug;23(8):879-885. doi: 10.1111/tmi.13081. Epub 2018 Jun 14. PMID:

29808960.

583: Kiondo P, Wamuyu-Maina G, Bimenya GS, Tumwesigye NM, Wandabwa J, Okong P.

Risk factors for pre-eclampsia in Mulago Hospital, Kampala, Uganda. Trop Med Int

Health. 2012 Apr;17(4):480-7. doi: 10.1111/j.1365-3156.2011.02926.x. Epub 2011

Dec 13. PMID: 22151898.

584: Matuja W, Janabi M, Kazema R, Mashuke D. Stroke subtypes in Black

Tanzanians: a retrospective study of computerized tomography scan diagnoses at

Muhimbili National Hospital, Dar es Salaam. Trop Doct. 2004 Jul;34(3):144-6.

doi: 10.1177/004947550403400305. PMID: 15267040.

585: Agarwal A, Kirwa K, Eliot MN, Alenezi F, Menya D, Mitter SS, Velazquez EJ,

Vedanthan R, Wellenius GA, Bloomfield GS. Household Air Pollution Is Associated

with Altered Cardiac Function among Women in Kenya. Am J Respir Crit Care Med.

2018 Apr 1;197(7):958-961. doi: 10.1164/rccm.201704-0832LE. PMID: 28925740;

PMCID: PMC6020413.

586: Bovet P, Gervasoni JP, Mkamba M, Balampama M, Lengeler C, Paccaud F. Low

utilization of health care services following screening for hypertension in Dar

es Salaam (Tanzania): a prospective population-based study. BMC Public Health.

2008 Dec 16;8:407. doi: 10.1186/1471-2458-8-407. PMID: 19087300; PMCID:

PMC2615777.

587: Grimaldi A, Ammirati E, Karam N, Vermi AC, de Concilio A, Trucco G, Aloi F,

Arioli F, Figini F, Ferrarello S, Sacco FM, Grottola R, D'Arbela PG, Alfieri O,

Marijon E, Freers J, Mirabel M. Cardiac surgery for patients with heart failure

due to structural heart disease in Uganda: access to surgery and outcomes.

Cardiovasc J Afr. 2014 Sep-Oct;25(5):204-11. doi: 10.5830/CVJA-2014-034. Epub

2014 Jul 29. PMID: 25073490; PMCID: PMC4241599.

588: Jenkins JM, Fife A, Baghai M, Dworakowski R. Neisseria elongata subsp

elongata infective endocarditis following endurance exercise. BMJ Case Rep. 2015

Dec 11;2015:bcr2015212415. doi: 10.1136/bcr-2015-212415. PMID: 26655669; PMCID:

PMC4680243.

589: Zack RM, Irema K, Kazonda P, Leyna GH, Liu E, Spiegelman D, Fawzi W,

Njelekela M, Killewo J, Danaei G. Determinants of high blood pressure and

barriers to diagnosis and treatment in Dar es Salaam, Tanzania. J Hypertens.

2016 Dec;34(12):2353-2364. doi: 10.1097/HJH.0000000000001117. PMID: 27648720;

PMCID: PMC5675737.

590: Muthuuri JM. Male infertility in a private Kenyan hospital. East Afr Med J.

2005 Jul;82(7):362-6. PMID: 16167710.

591: Gonick HC, Behari JR. Is lead exposure the principal cause of essential

hypertension? Med Hypotheses. 2002 Sep;59(3):239-46. doi:

10.1016/s0306-9877(02)00207-4. PMID: 12208146.

592: Leak SA, Mmbaga LG, Mkwizu EW, Mapendo PJ, Henke O. Hematological

malignancies in East Africa-Which cancers to expect and how to provide services.

PLoS One. 2020 May 6;15(5):e0232848. doi: 10.1371/journal.pone.0232848. PMID:

32374771; PMCID: PMC7202623.

593: Lalitha R, Opio CK. A missed diagnosis or a masquerading disease: back to

the basics. Pan Afr Med J. 2013 May 21;15:29. doi:

10.11604/pamj.2013.15.29.2039. PMID: 24009805; PMCID: PMC3758842.

594: Blank E, Tuikong N, Misoi L, Kamano J, Hutchinson C, Kimaiyo S, Fustera V,

Were M, Vedanthan R. Usability of implementing a tablet-based decision support

and integrated record- keeping (DESIRE) tool in the nurse management of

hypertension in rural Kenya. Stud Health Technol Inform. 2013;192:1002. PMID:

23920776; PMCID: PMC4074025.

595: Ogendo SW. Warfarin-related bleeding following open heart surgery in

Nairobi. East Afr Med J. 2001 Mar;78(3):139-43. doi: 10.4314/eamj.v78i3.9080.

PMID: 12002054.

596: Fredrick F, Ruggajo PJ, Makanga G, Shija CK, Amdemariam M, Rugwizagonga B,

Kitinya JN. Thirty years old lady with nephrotic syndrome: a case of biopsy

proven lupus nephritis in Tanzania. Tanzan J Health Res. 2014 Apr;16(2):127-30.

doi: 10.4314/thrb.v16i2.9. PMID: 26875307.

597: Pariyo GW, Greenleaf AR, Gibson DG, Ali J, Selig H, Labrique AB, Al Kibria

GM, Khan IA, Masanja H, Flora MS, Ahmed S, Hyder AA. Does mobile phone survey

method matter? Reliability of computer-assisted telephone interviews and

interactive voice response non-communicable diseases risk factor surveys in low

and middle income countries. PLoS One. 2019 Apr 10;14(4):e0214450. doi:

10.1371/journal.pone.0214450. PMID: 30969975; PMCID: PMC6457489.

598: Shayo FK, Lutale J. Albuminuria in patients with chronic obstructive

pulmonary disease: a cross-sectional study in an African patient cohort. BMC

Pulm Med. 2018 Jul 31;18(1):125. doi: 10.1186/s12890-018-0694-5. PMID: 30064397;

PMCID: PMC6066916.

599: Kato I, Tumaini B, Pallangyo K. Prevalence of non-communicable diseases

among individuals with HIV infection by antiretroviral therapy status in Dar es

Salaam, Tanzania. PLoS One. 2020 Jul 9;15(7):e0235542. doi:

10.1371/journal.pone.0235542. PMID: 32645054; PMCID: PMC7347196.

600: Sobry A, Kizito W, Van den Bergh R, Tayler-Smith K, Isaakidis P, Cheti E,

Kosgei RJ, Vandenbulcke A, Ndegwa Z, Reid T. Caseload, management and treatment

outcomes of patients with hypertension and/or diabetes mellitus in a primary

health care programme in an informal setting. Trop Med Int Health. 2014

Jan;19(1):47-57. doi: 10.1111/tmi.12210. PMID: 24851259.

601: Kotwani P, Balzer L, Kwarisiima D, Clark TD, Kabami J, Byonanebye D,

Bainomujuni B, Black D, Chamie G, Jain V, Thirumurthy H, Kamya MR, Geng EH,

Petersen ML, Havlir DV, Charlebois ED; SEARCH Collaboration. Evaluating linkage

to care for hypertension after community-based screening in rural Uganda. Trop

Med Int Health. 2014 Apr;19(4):459-68. doi: 10.1111/tmi.12273. Epub 2014 Feb 3.

PMID: 24495307; PMCID: PMC4118739.

602: Schell CO, Castegren M, Lugazia E, Blixt J, Mulungu M, Konrad D, Baker T.

Severely deranged vital signs as triggers for acute treatment modifications on

an intensive care unit in a low-income country. BMC Res Notes. 2015 Jul

25;8:313. doi: 10.1186/s13104-015-1275-9. Erratum in: BMC Res Notes. 2017 Aug

2;10 (1):365. PMID: 26205670; PMCID: PMC5501369.

603: Dahlén GH, Ekstedt B. The importance of the relation between lipoprotein(a)

and lipids for development of atherosclerosis and cardiovascular disease. J

Intern Med. 2001 Sep;250(3):265-7. doi: 10.1046/j.1365-2796.2001.00889.x. PMID:

11555135.

604: John CC, Opoka RO, Latham TS, Hume HA, Nabaggala C, Kasirye P, Ndugwa CM,

Lane A, Ware RE. Hydroxyurea Dose Escalation for Sickle Cell Anemia in Sub-

Saharan Africa. N Engl J Med. 2020 Jun 25;382(26):2524-2533. doi:

10.1056/NEJMoa2000146. PMID: 32579813.

605: Njelekela MA, Liu E, Mpembeni R, Muhihi A, Mligiliche N, Spiegelman D,

Finkelstein JL, Fawzi WW, Willett WC, Mtabaji J. Socio-economic status,

urbanization, and cardiometabolic risk factors among middle-aged adults in

Tanzania. East Afr J Public Health. 2011 Sep;8(3):216-23. PMID: 23120960.

606: Kagaruki GB, Mayige MT, Ngadaya ES, Kimaro GD, Kalinga AK, Kilale AM, Kahwa

AM, Materu GS, Mfinanga SG. Magnitude and risk factors of non-communicable

diseases among people living with HIV in Tanzania: a cross sectional study from

Mbeya and Dar es Salaam regions. BMC Public Health. 2014 Sep 2;14:904. doi:

10.1186/1471-2458-14-904. PMID: 25183300; PMCID: PMC4161834.

607: Godown J, Lu JC, Beaton A, Sable C, Mirembe G, Sanya R, Aliku T, Yu S,

Lwabi P, Webb CL, Ensing GJ. Handheld echocardiography versus auscultation for

detection of rheumatic heart disease. Pediatrics. 2015 Apr;135(4):e939-44. doi:

10.1542/peds.2014-2774. Epub 2015 Mar 16. PMID: 25780068.

608: Nakibuuka J, Sajatovic M, Nankabirwa J, Furlan AJ, Kayima J, Ddumba E,

Katabira E, Byakika-Tusiime J. Stroke-Risk Factors Differ between Rural and

Urban Communities: Population Survey in Central Uganda. Neuroepidemiology.

2015;44(3):156-65. doi: 10.1159/000381453. Epub 2015 May 7. PMID: 25967045;

PMCID: PMC4458230.

609: Ahimbisibwe C, Kwizera R, Ndyetukira JF, Kugonza F, Sadiq A, Hullsiek KH,

Williams DA, Rhein J, Boulware DR, Meya DB. Management of amphotericin-induced

phlebitis among HIV patients with cryptococcal meningitis in a resource-limited

setting: a prospective cohort study. BMC Infect Dis. 2019 Jun 26;19(1):558. doi:

10.1186/s12879-019-4209-7. PMID: 31242860; PMCID: PMC6595678.

610: Edwards JK, Bygrave H, Van den Bergh R, Kizito W, Cheti E, Kosgei RJ, Sobry

A, Vandenbulcke A, Vakil SN, Reid T. HIV with non-communicable diseases in

primary care in Kibera, Nairobi, Kenya: characteristics and outcomes 2010-2013.

Trans R Soc Trop Med Hyg. 2015 Jul;109(7):440-6. doi: 10.1093/trstmh/trv038.

Epub 2015 May 21. PMID: 25997923.

611: Mugendi GA, Nyamu DG, Okalebo FA, Nyamweya NN, Ndwiga S, Mwangi M.

COMPARISON OF LOSARTAN AND ENALAPRIL EFFECTS ON RENAL FUNCTION IN HYPERTENSIVE

ADULTS WITH CHRONIC KIDNEY DISEASE AT A KENYAN REFERRAL HOSPITAL. East Afr Med

J. 2014 Apr;91(4):125-32. PMID: 26859031.

612: Merchant AT, Msamanga G, Villamor E, Saathoff E, O'brien M, Hertzmark E,

Hunter DJ, Fawzi WW. Multivitamin supplementation of HIV-positive women during

pregnancy reduces hypertension. J Nutr. 2005 Jul;135(7):1776-81. doi:

10.1093/jn/135.7.1776. PMID: 15987864.

613: Were FH, Moturi MC, Gottesfeld P, Wafula GA, Kamau GN, Shiundu PM. Lead

exposure and blood pressure among workers in diverse industrial plants in Kenya.

J Occup Environ Hyg. 2014;11(11):706-15. doi: 10.1080/15459624.2014.908258.

PMID: 24690073.

614: Naburi H, Assenga E, Patel S, Massawe A, Manji K. Class II pentalogy of

Cantrell. BMC Res Notes. 2015 Jul 29;8:318. doi: 10.1186/s13104-015-1293-7.

PMID: 26219662; PMCID: PMC4517504.

615: Bower JH, Mwendo E, Walker R, Maro V, Enquosellasie F, Ali S. Validity of a

screening instrument for neurologic disability in resource-poor African

communities. J Neurol Sci. 2012 Sep 15;320(1-2):52-5. doi:

10.1016/j.jns.2012.06.007. Epub 2012 Jul 11. PMID: 22795389; PMCID: PMC3414652.

616: Bahiru E, Temu T, Gitura B, Farquhar C, Huffman MD, Bukachi F.

Presentation, management and outcomes of acute coronary syndrome: a registry

study from Kenyatta National Hospital in Nairobi, Kenya. Cardiovasc J Afr. 2018

Jul/Aug 23;29(4):225-230. doi: 10.5830/CVJA-2018-017. Epub 2018 May 24. PMID:

29878033.

617: Mbui JM, Oluka MN, Guantai EM, Sinei KA, Achieng L, Baker A, Jande M,

Massele A, Godman B. Prescription patterns and adequacy of blood pressure

control among adult hypertensive patients in Kenya; findings and implications.

Expert Rev Clin Pharmacol. 2017 Nov;10(11):1263-1271. doi:

10.1080/17512433.2017.1371590. Epub 2017 Sep 5. PMID: 28831829.

618: Mtuya C, Cleland CR, Philippin H, Paulo K, Njau B, Makupa WU, Hall C, Hall

A, Courtright P, Mushi D. Reasons for poor follow-up of diabetic retinopathy

patients after screening in Tanzania: a cross-sectional study. BMC Ophthalmol.

2016 Jul 19;16:115. doi: 10.1186/s12886-016-0288-z. PMID: 27435362; PMCID:

PMC4950081.

619: Bergmark R, Bergmark B, Blander J, Fataki M, Janabi M. Burden of disease

and barriers to the diagnosis and treatment of group a beta-hemolytic

streptococcal pharyngitis for the prevention of rheumatic heart disease in Dar

Es Salaam, Tanzania. Pediatr Infect Dis J. 2010 Dec;29(12):1135-7. doi:

10.1097/inf.0b013e3181edf475. PMID: 21155123.

620: Kimeu R, Kariuki C. Assessment of the management of acute myocardial

infarction patients and their outcomes at the Nairobi Hospital from January 2007

to June 2009. Cardiovasc J Afr. 2016 Jul/Aug;27(4):218-221. doi:

10.5830/CVJA-2015-091. PMID: 27841909; PMCID: PMC5340900.

621: Robertson NJ, Hagmann CF, Acolet D, Allen E, Nyombi N, Elbourne D, Costello

A, Jacobs I, Nakakeeto M, Cowan F. Pilot randomized trial of therapeutic

hypothermia with serial cranial ultrasound and 18-22 month follow-up for

neonatal encephalopathy in a low resource hospital setting in Uganda: study

protocol. Trials. 2011 Jun 4;12:138. doi: 10.1186/1745-6215-12-138. PMID:

21639927; PMCID: PMC3127769.

622: Pönnighaus M, Grosser S, Baum HP, Mischke D, Kowalzick L. Sporotrichose als

Ursache eines Ulcus cruris [Sporotrichosis as the cause of a leg ulcer].

Hautarzt. 2003 Jan;54(1):64-6. German. doi: 10.1007/s00105-002-0400-6. Epub 2002

Dec 20. PMID: 12567260.

623: Asiki G, Stockdale L, Kasamba I, Vudriko T, Tumwekwase G, Johnston T,

Kaleebu P, Kamali A, Seeley J, Newton R. Pilot study of antibodies against

varicella zoster virus and human immunodeficiency virus in relation to the risk

of developing stroke, nested within a rural cohort in Uganda. Trop Med Int

Health. 2015 Oct;20(10):1306-10. doi: 10.1111/tmi.12556. Epub 2015 Jul 23. PMID:

26083834.

624: Wanjohi FW, Otieno FC, Ogola EN, Amayo EO. Nephropathy in patients with

recently diagnosed type 2 diabetes mellitus in black Africans. East Afr Med J.

2002 Aug;79(8):399-404. doi: 10.4314/eamj.v79i8.8824. PMID: 12638839.

625: Ploutz M, Lu JC, Scheel J, Webb C, Ensing GJ, Aliku T, Lwabi P, Sable C,

Beaton A. Handheld echocardiographic screening for rheumatic heart disease by

non-experts. Heart. 2016 Jan;102(1):35-9. doi: 10.1136/heartjnl-2015-308236.

Epub 2015 Oct 5. PMID: 26438784.

626: Chalya PL, Kayange NM, Rambau PF, Manyama M, Gilyoma JM. Surgical

management of vascular anomalies in children at a tertiary care hospital in a

resource-limited setting: a Tanzanian experience with 134 patients. BMC Res

Notes. 2015 Nov 30;8:732. doi: 10.1186/s13104-015-1718-3. PMID: 26621505; PMCID:

PMC4666117.

627: Bustinduy AL, Luzinda K, Mpoya S, Gothard P, Stone N, Wright S, Stothard

JR. Endomyocardial fibrosis (EMF) in a Ugandan child with advanced hepatosplenic

schistosomiasis: coincidence or connection? Am J Trop Med Hyg. 2014

Oct;91(4):798-800. doi: 10.4269/ajtmh.14-0156. Epub 2014 Jul 7. PMID: 25002295;

PMCID: PMC4183407.

628: Lutale JJ, Thordarson H, Gulam-Abbas Z, Vetvik K, Gerdts E. Prevalence and

covariates of electrocardiographic left ventricular hypertrophy in diabetic

patients in Tanzania. Cardiovasc J Afr. 2008 Jan-Feb;19(1):8-14. PMID: 18320079;

PMCID: PMC3975314.

629: Kagaruki GB, Mayige MT, Ngadaya ES, Kilale AM, Kahwa A, Shao AF, Kimaro GD,

Manga CM, Mbata D, Materu GS, Masumo RM, Mfinanga SG. Knowledge and perception

on type2 diabetes and hypertension among HIV clients utilizing care and

treatment services: a cross sectional study from Mbeya and Dar es Salaam regions

in Tanzania. BMC Public Health. 2018 Jul 28;18(1):928. doi:

10.1186/s12889-018-5639-7. PMID: 30055591; PMCID: PMC6064130.

630: Chillo P, Lwakatare J, Lutale J, Gerdts E. Increased relative wall

thickness is a marker of subclinical cardiac target-organ damage in African

diabetic patients. Cardiovasc J Afr. 2012 Sep;23(8):435-41. doi:

10.5830/CVJA-2012-023. Epub 2012 Mar 20. PMID: 22447437; PMCID: PMC3721891.

631: Beaton A, Okello E, Engelman D, Grobler A, Scheel A, DeWyer A, Sarnacki R,

Omara IO, Rwebembera J, Sable C, Steer A. Determining the impact of Benzathine

penicillin G prophylaxis in children with latent rheumatic heart disease (GOAL

trial): Study protocol for a randomized controlled trial. Am Heart J. 2019

Sep;215:95-105. doi: 10.1016/j.ahj.2019.06.001. Epub 2019 Jun 8. PMID: 31301533.

632: Ssinabulya I, Kayima J, Longenecker C, Luwedde M, Semitala F, Kambugu A,

Ameda F, Bugeza S, McComsey G, Freers J, Nakanjako D. Subclinical

atherosclerosis among HIV-infected adults attending HIV/AIDS care at two large

ambulatory HIV clinics in Uganda. PLoS One. 2014 Feb 28;9(2):e89537. doi:

10.1371/journal.pone.0089537. PMID: 24586854; PMCID: PMC3938501.

633: Akan G, Kisenge P, Sanga TS, Mbugi E, Adolf I, Turkcan MK, Janabi M, Atalar

F. Common SNP-based haplotype analysis of the 9p21.3 gene locus as predictor

coronary artery disease in Tanzanian population. Cell Mol Biol (Noisy-le-grand).

2019 Jul 31;65(6):33-43. PMID: 31472045.

634: Gitau AM, Ng'ang'a ZW, Sigilai W, Bii C, Mwangi M. Fungal infections among

diabetic foot ulcer- patients attending diabetic clinic in Kenyatta National

Hospital, Kenya. East Afr Med J. 2011 Jan;88(1):9-17. PMID: 24968597.

635: Young JR, Sawe HR, Mfinanga JA, Nshom E, Helm E, Moore CG, Runyon MS,

Reynolds SL. Subdissociative intranasal ketamine plus standard pain therapy

versus standard pain therapy in the treatment of paediatric sickle cell disease

vaso-occlusive crises in resource-limited settings: study protocol for a

randomised controlled trial. BMJ Open. 2017 Jul 10;7(7):e017190. doi:

10.1136/bmjopen-2017-017190. PMID: 28698351; PMCID: PMC5541700.

636: Chillo P, Rieck AE, Lwakatare J, Lutale J, Gerdts E. Left atrial volume

index as a marker of left ventricular diastolic dysfunction in asymptomatic

Tanzanian diabetic patients. Blood Press. 2013 Apr;22(2):86-93. doi:

10.3109/08037051.2012.707351. Epub 2012 Aug 2. PMID: 22853716.

637: Gaziano TA, Abrahams-Gessel S, Alam S, Alam D, Ali M, Bloomfield G,

Carrillo-Larco RM, Dorairaj P, Gutierrez L, Irazola V, Levitt NS, Miranda JJ,

Bernabe-Ortiz A, Pandya A, Rubinstein A, Steyn K, Xavier D, Yan LL. Comparison

of Nonblood-Based and Blood-Based Total CV Risk Scores in Global Populations.

Glob Heart. 2016 Mar;11(1):37-46.e2. doi: 10.1016/j.gheart.2015.12.003. PMID:

27102021.

638: Yang IT, Hemphill LC, Kim JH, Bibangambah P, Sentongo R, Kakuhire B,

Plutzky J, Boum Y 2nd, Tsai AC, Okello S, Siedner MJ. To fast or not to fast:

Lipid measurement and cardiovascular disease risk estimation in rural sub-

Saharan Africa. J Glob Health. 2020 Jun;10(1):010407. doi:

10.7189/jogh.10.010407. PMID: 32257155; PMCID: PMC7101029.

639: Grandière-Pérez L, Caumes E. Corticosteroids for watershed infarction in

acute schistosomiasis. Clin Infect Dis. 2013 Sep;57(6):918-9. doi:

10.1093/cid/cit385. Epub 2013 Jun 11. PMID: 23759344.

640: Gowda R, Cartwright K, Bremner JA, Green ST. Yellow fever vaccine: a

successful vaccination of an immunocompromised patient. Eur J Haematol. 2004

Apr;72(4):299-301. doi: 10.1111/j.1600-0609.2004.00218.x. PMID: 15089771.

641: Yacoub S, Lang HJ, Shebbe M, Timbwa M, Ohuma E, Tulloh R, Maitland K.

Cardiac function and hemodynamics in Kenyan children with severe malaria. Crit

Care Med. 2010 Mar;38(3):940-5. doi: 10.1097/CCM.0b013e3181cd114a. PMID:

20068463.

642: Ngalesoni FN, Ruhago GM, Mori AT, Robberstad B, Norheim OF. Cost-

effectiveness of medical primary prevention strategies to reduce absolute risk

of cardiovascular disease in Tanzania: a Markov modelling study. BMC Health Serv

Res. 2016 May 17;16:185. doi: 10.1186/s12913-016-1409-3. PMID: 27184802; PMCID:

PMC4869389.

643: Kanyi JK, Ogada TV, Oloo MJ, Parker RK. Burr-Hole Craniostomy for Chronic

Subdural Hematomas by General Surgeons in Rural Kenya. World J Surg. 2018

Jan;42(1):40-45. doi: 10.1007/s00268-017-4143-z. PMID: 28741193.

644: Buggey J, Yun L, Hung CL, Kityo C, Mirembe G, Erem G, Truong T, Ssinabulya

I, Tang WHW, Hoit BD, McComsey GA, Longenecker CT. HIV and pericardial fat are

associated with abnormal cardiac structure and function among Ugandans. Heart.

2020 Jan;106(2):147-153. doi: 10.1136/heartjnl-2019-315346. Epub 2019 Sep 19.

PMID: 31537637.

645: Jones ES, Spence JD, Mcintyre AD, Nondi J, Gogo K, Akintunde A, Hackam DG,

Rayner BL. High Frequency of Variants of Candidate Genes in Black Africans with

Low Renin-Resistant Hypertension. Am J Hypertens. 2017 May 1;30(5):478-483. doi:

10.1093/ajh/hpw167. PMID: 28052878.

646: Vos AG, Chersich MF, Klipstein-Grobusch K, Zuithoff P, Moorhouse MA, Lalla-

Edward ST, Kambugu A, Kumarasamy N, Grobbee DE, Barth RE, Venter WD. Lipid

levels, insulin resistance and cardiovascular risk over 96 weeks of

antiretroviral therapy: a randomised controlled trial comparing low-dose

stavudine and tenofovir. Retrovirology. 2018 Dec 14;15(1):77. doi:

10.1186/s12977-018-0460-z. PMID: 30547820; PMCID: PMC6295103.

647: Teriö M, Eriksson G, Kamwesiga JT, Guidetti S. What's in it for me? A

process evaluation of the implementation of a mobile phone-supported

intervention after stroke in Uganda. BMC Public Health. 2019 May 14;19(1):562.

doi: 10.1186/s12889-019-6849-3. PMID: 31088411; PMCID: PMC6518972.

648: Warf BC. Comparison of endoscopic third ventriculostomy alone and combined

with choroid plexus cauterization in infants younger than 1 year of age: a

prospective study in 550 African children. J Neurosurg. 2005 Dec;103(6

Suppl):475-81. doi: 10.3171/ped.2005.103.6.0475. PMID: 16383244.

649: Okello E, Beaton A, Mondo CK, Kruszka P, Kiwanuka N, Odoi-Adome R, Freers

J. Rheumatic heart disease in Uganda: the association between MHC class II HLA

DR alleles and disease: a case control study. BMC Cardiovasc Disord. 2014 Feb

28;14:28. doi: 10.1186/1471-2261-14-28. PMID: 24581333; PMCID: PMC3943278.

650: Kotlyar S, Olupot-Olupot P, Nteziyaremye J, Akech SO, Uyoga S, Muhindo R,

Moore CL, Maitland K. Assessment of Myocardial Function and Injury by

Echocardiography and Cardiac Biomarkers in African Children With Severe

Plasmodium falciparum Malaria. Pediatr Crit Care Med. 2018 Mar;19(3):179-185.

doi: 10.1097/PCC.0000000000001411. PMID: 29206727; PMCID: PMC5835359.

651: Mayito J, Mungoma M, Kakande B, Okello DC, Wanzira H, Kayima J, Mondo CK.

Angiotensin II status and sympathetic activation among hypertensive patients in

Uganda: a cross-sectional study. BMC Res Notes. 2015 Oct 20;8:586. doi:

10.1186/s13104-015-1544-7. PMID: 26486596; PMCID: PMC4612413.

652: Nthumba P, Cavadas PC, Landin L. The tropical diabetic hand syndrome: a

surgical perspective. Ann Plast Surg. 2013 Jan;70(1):42-6. doi:

10.1097/SAP.0b013e3182305e96. PMID: 22156888.

653: Regenhardt RW, Biseko MR, Shayo AF, Mmbando TN, Grundy SJ, Xu A, Saadi A,

Wibecan L, Kharal GA, Parker R, Klein JP, Mateen FJ, Okeng'o K. Opportunities

for intervention: stroke treatments, disability and mortality in urban Tanzania.

Int J Qual Health Care. 2019 Jun 1;31(5):385-392. doi: 10.1093/intqhc/mzy188.

PMID: 30165650; PMCID: PMC6528701.

654: Karau PB, Kirna B, Amayo E, Joshi M, Ngare S, Muriira G. The prevalence of

vitamin D deficiency among patients with type 2 diabetes seen at a referral

hospital in Kenya. Pan Afr Med J. 2019 Sep 17;34:38. doi:

10.11604/pamj.2019.34.38.18936. PMID: 31762905; PMCID: PMC6859033.

655: Evans GF, Shirk A, Muturi P, Soliman EZ. Feasibility of Using Mobile ECG

Recording Technology to Detect Atrial Fibrillation in Low-Resource Settings.

Glob Heart. 2017 Dec;12(4):285-289. doi: 10.1016/j.gheart.2016.12.003. Epub 2017

Mar 13. PMID: 28302547.

656: Ogendo SW. Pattern of anticoagulation control after heart valve surgery at

the Kenyatta National Hospital, Nairobi. East Afr Med J. 2000 Jul;77(7):354-8.

doi: 10.4314/eamj.v77i7.46667. PMID: 12862152.

657: Batte A, Lwabi P, Lubega S, Kiguli S, Otwombe K, Chimoyi L, Nabatte V,

Karamagi C. Wasting, underweight and stunting among children with congenital

heart disease presenting at Mulago hospital, Uganda. BMC Pediatr. 2017 Jan

11;17(1):10. doi: 10.1186/s12887-017-0779-y. PMID: 28077108; PMCID: PMC5225644.

658: Mbalilaki JA, Hellènius ML, Masesa Z, Høstmark AT, Sundquist J, Strømme SB.

Physical activity and blood lipids in rural and urban Tanzanians. Nutr Metab

Cardiovasc Dis. 2007 Jun;17(5):344-8. doi: 10.1016/j.numecd.2006.03.003. Epub

2006 Jul 7. PMID: 17134959.

659: Kiyeng JC, Siika A, Koech C, Bloomfield GS. Definite hereditary hemorrhagic

telangiectasia in a 60-year-old black Kenyan woman: a case report. J Med Case

Rep. 2016 May 25;10(1):126. doi: 10.1186/s13256-016-0909-4. PMID: 27225226;

PMCID: PMC4880959.

660: Beaton A, Aliku T, Dewyer A, Jacobs M, Jiang J, Longenecker CT, Lubega S,

McCarter R, Mirabel M, Mirembe G, Namuyonga J, Okello E, Scheel A, Tenywa E,

Sable C, Lwabi P. Latent Rheumatic Heart Disease: Identifying the Children at

Highest Risk of Unfavorable Outcome. Circulation. 2017 Dec 5;136(23):2233-2244.

doi: 10.1161/CIRCULATIONAHA.117.029936. Epub 2017 Sep 27. PMID: 28972003; PMCID:

PMC5716883.

661: Kaddumukasa MN, Katabira E, Sajatovic M, Pundik S, Kaddumukasa M, Goldstein

LB. Influence of Dietary Salt Knowledge, Perceptions, and Beliefs on Consumption

Choices after Stroke in Uganda. J Stroke Cerebrovasc Dis. 2017

Dec;26(12):2935-2942. doi: 10.1016/j.jstrokecerebrovasdis.2017.07.016. Epub 2017

Aug 12. PMID: 28807485; PMCID: PMC5698090.

662: Kabesha TB, Kabesha D, Maloba V, Mwamba C, Chenge B, Mukalay A. Résultats

de la trabéculectomie associée aux anti-VGEF dans le traitement du glaucome

néovasculaire compliquant les occlusions veineuses rétiniennes (à propos de

21 cas, suivis à la clinique ophtalmologique de Bukavu du 1er janvier au

31 décembre 2015) [Results of trabeculectomy with anti-VGEF therapy in the

treatment of neovascular glaucoma secondary to retinal vein occlusion (report of

21 cases followed at Bukavu Eye Clinic from January 1 to December 31th, 2015)].

J Fr Ophtalmol. 2017 Jan;40(1):17-21. French. doi: 10.1016/j.jfo.2016.08.009.

Epub 2016 Nov 22. PMID: 27884546.

663: Ogeng'o JA, Obimbo MM, Olabu BO, Gatonga PM, Ong'era D. Pulmonary

thromboembolism in an East African tertiary referral hospital. J Thromb

Thrombolysis. 2011 Oct;32(3):386-91. doi: 10.1007/s11239-011-0607-4. PMID:

21674133.

664: Negishi H, Njelekela M, Ikeda K, Sagara M, Noguchi T, Kuga S, Kanda T, Liu

L, Nara Y, Tagami M, Yamori Y. Assessment of in vivo oxidative stress in

hypertensive rats and hypertensive subjects in Tanzania, Africa. Hypertens Res.

2000 May;23(3):285-9. doi: 10.1291/hypres.23.285. PMID: 10821140.

665: Jowi JO, Mativo PM, Musoke SS. Clinical and laboratory characteristics of

hospitalised patients with neurological manifestations of HIV/AIDS at the

Nairobi hospital. East Afr Med J. 2007 Feb;84(2):67-76. doi:

10.4314/eamj.v84i2.9506. PMID: 17598667.

666: Nabunnya YB, Kayima J, Longenecker CT, Josephson RA, Freers J. The safety

and efficacy of prednisolone in preventing reaccumulation of ascites among

endomyocardial fibrosis patients in Uganda: a randomized clinical trial. BMC Res

Notes. 2015 Dec 15;8:783. doi: 10.1186/s13104-015-1761-0. PMID: 26666319; PMCID:

PMC4678569.

667: Kanyi J, Karwa R, Pastakia SD, Manji I, Manyara S, Saina C. Venous

Thromboembolism Requiring Extended Anticoagulation Among HIV-Infected Patients

in a Rural, Resource-Constrained Setting in Western Kenya. Ann Pharmacother.

2017 May;51(5):380-387. doi: 10.1177/1060028016686106. Epub 2017 Jan 6. PMID:

28056524.

668: Okello MO, Mung'ayi V, Adam R, Kabugi J. A comparison of risk of

hypotension using standard doses of remifentanil versus dexmedetomidine

infusions in adult patients undergoing surgery under general anaesthesia at the

Aga Khan University Hospital, Nairobi. Afr Health Sci. 2018 Dec;18(4):1267-1282.

doi: 10.4314/ahs.v18i4.48. PMID: 30766593; PMCID: PMC6354876.

669: Ekwom PE. Systemic lupus erythematosus (SLE) at the Kenyatta National

Hospital. Clin Rheumatol. 2013 Aug;32(8):1215-7. doi: 10.1007/s10067-013-2217-3.

Epub 2013 Mar 3. PMID: 23456415.

670: Musoke C, Mondo CK, Okello E, Zhang W, Kakande B, Nyakoojo W, Freers J.

Benzathine penicillin adherence for secondary prophylaxis among patients

affected with rheumatic heart disease attending Mulago Hospital. Cardiovasc J

Afr. 2013 May;24(4):124-9. doi: 10.5830/CVJA-2013-022. PMID: 24217043; PMCID:

PMC3721822.

671: Uwineza A, Hitayezu J, Jamar M, Caberg JH, Murorunkwere S, Janvier N, Bours

V, Mutesa L. Cytogenetic Studies of Rwandan Pediatric Patients Presenting with

Global Developmental Delay, Intellectual Disability and/or Multiple Congenital

Anomalies. J Trop Pediatr. 2016 Feb;62(1):38-45. doi: 10.1093/tropej/fmv065.

Epub 2015 Oct 27. PMID: 26507407; PMCID: PMC4935782.

672: Varwani MH, Jeilan M, Ngunga M, Barasa A. Outcomes in patients with acute

coronary syndrome in a referral hospital in sub-Saharan Africa. Cardiovasc J

Afr. 2019 Jan/Feb 23;30(1):29-33. doi: 10.5830/CVJA-2018-066. Epub 2018 Dec 4.

PMID: 30534849.

673: Makani J, Kirkham FJ, Komba A, Ajala-Agbo T, Otieno G, Fegan G, Williams

TN, Marsh K, Newton CR. Risk factors for high cerebral blood flow velocity and

death in Kenyan children with Sickle Cell Anaemia: role of haemoglobin oxygen

saturation and febrile illness. Br J Haematol. 2009 May;145(4):529-32. doi:

10.1111/j.1365-2141.2009.07660.x. Epub 2009 Mar 27. PMID: 19344425; PMCID:

PMC3001030.

674: Bamrah S, Mbithi A, Mermin JH, Boo T, Bunnell RE, Sharif S, Cookson ST. The

impact of post-election violence on HIV and other clinical services and on

mental health-Kenya, 2008. Prehosp Disaster Med. 2013 Feb;28(1):43-51. doi:

10.1017/S1049023X12001665. Epub 2012 Nov 26. PMID: 23177022.

675: Kabanywanyi AM, Baiden R, Ali AM, Mahende MK, Ogutu BR, Oduro A, Tinto H,

Gyapong M, Sie A, Sevene E, Macete E, Owusu-Agyei S, Adjei A, Compaoré G, Valea

I, Osei I, Yawson A, Adjuik M, Akparibo R, Kakolwa MA, Abdulla S, Binka F.

Multi-Country Evaluation of Safety of Dihydroartemisinin/Piperaquine Post-

Licensure in African Public Hospitals with Electrocardiograms. PLoS One. 2016

Oct 20;11(10):e0164851. doi: 10.1371/journal.pone.0164851. PMID: 27764178;

PMCID: PMC5072600.

676: Matthes T, Tullen E, Poole J, Banks J, Nagy M, Stelling MJ, Boehlen F,

Michel M, Beris P, Hustinx H, Crew V, Daniels G. Acquired and transient RBC CD55

deficiency (Inab phenotype) and anti-IFC. Transfusion. 2002 Nov;42(11):1448-57.

doi: 10.1046/j.1537-2995.2002.00214.x. PMID: 12421218.

677: Smit MR, Ochomo EO, Waterhouse D, Kwambai TK, Abong'o BO, Bousema T, Bayoh

NM, Gimnig JE, Samuels AM, Desai MR, Phillips-Howard PA, Kariuki SK, Wang D, Ter

Kuile FO, Ward SA, Aljayyoussi G. Pharmacokinetics-Pharmacodynamics of High-Dose

Ivermectin with Dihydroartemisinin-Piperaquine on Mosquitocidal Activity and QT-

Prolongation (IVERMAL). Clin Pharmacol Ther. 2019 Feb;105(2):388-401. doi:

10.1002/cpt.1219. Epub 2018 Oct 9. PMID: 30125353; PMCID: PMC6585895.

678: Ayala-Ramirez M, Habra MA, Busaidy N, Cote G, Rich T, Waguespack S, Jimenez

C. A catecholamine crisis on Mount Kilimanjaro: a hypoxia effect? J Travel Med.

2010 Nov-Dec;17(6):424-6. doi: 10.1111/j.1708-8305.2010.00463.x. PMID: 21050326.

679: Mutonga DM, Mureithi MW, Ngugi NN, Otieno FCF. Bacterial isolation and

antibiotic susceptibility from diabetic foot ulcers in Kenya using

microbiological tests and comparison with RT-PCR in detection of S. aureus and

MRSA. BMC Res Notes. 2019 Apr 29;12(1):244. doi: 10.1186/s13104-019-4278-0.

PMID: 31036061; PMCID: PMC6489269.

680: Buchacz K, Weidle PJ, Moore D, Were W, Mermin J, Downing R, Kigozi A,

Borkowf CB, Ndazima V, Brooks JT. Changes in lipid profile over 24 months among

adults on first-line highly active antiretroviral therapy in the home-based AIDS

care program in rural Uganda. J Acquir Immune Defic Syndr. 2008 Mar

1;47(3):304-11. doi: 10.1097/qai.0b013e31815e7453. PMID: 18398971.

681: Walong E, Rogena E, Sabai D. Primary immunodeficiency diagnosed at autopsy:

a case report. BMC Res Notes. 2014 Jul 4;7:425. doi: 10.1186/1756-0500-7-425.

PMID: 24996427; PMCID: PMC4094674.

**Cardiovascular disease sub Saharan Africa** **= 122,000 results (Google Scholar)**

**Cardiovascular disease = 29 results (African Journal Online)**
